# Electrophilic substitution in poly(ethylene terephthalate) waste for upcycling to functional metal–organic frameworks

**DOI:** 10.1039/d5mh02440d

**Published:** 2026-09-02

**Authors:** Daniil Bryankin, Aleksey Ivanov, Natalia S. Soldatova, Mekhman Yusubov, Shaghayegh Naghdi, Dominik Eder, Markus Valtiner, Haritz Sardon, Pavel S. Postnikov, Olga Guselnikova

**Affiliations:** a Research School of Chemistry and Applied Biomedical Sciences, Tomsk Polytechnic University Lenin Avenue 30 Tomsk 634050 Russian Federation postnikov@tpu.ru olga.guselnikova@tuwien.ac.at; b Institute of Applied Physics, Vienna University of Technology 1040 Vienna Austria; c Institute of Materials Chemistry, Vienna University of Technology Vienna 1060 Austria; d POLYMAT, University of the Basque Country UPV/EHU, Joxe Mari Korta Center Avda. Tolosa 72 20018 Donostia-San Sebastian Spain; e N.N. Vorozhtsov Institute of Organic Chemistry Pr. Ak. Lavrentieva 9 Novosibirsk 630090 Russian Federation

## Abstract

In contrast to polymer-to-polymer recycling of waste polyethylene terephthalate (wPET), chemical upcycling into added-value molecules beyond terephthalic acid is a promising strategy. Among possible transformations, electrophilic substitution offers a direct path, yet has remained unrealized due to the electron-deficient nature of the aromatic ring. Here, we demonstrate that substitution and depolymerisation can be merged in a single step, enabling the direct electrophilic functionalisation of wPET – a transformation previously considered inaccessible. We report a one-step method for electrophilic functionalisation of various wPET sources, such as post-consumer food boxes, bottles, industrial blends, and textile residues – into 2-nitroterephthalic acid (TA-NO_2_) and 2-iodoterephthalic acid (TA-I). Mechanistic studies reveal that PET hydrolysis precedes nitration under the investigated conditions, alongside partial electrophilic functionalisation of the polymer surface. Upcycling optimisation was guided by the environmental energy impact factor, providing a quantitative metric to balance yield, resource efficiency, and sustainability. The developed protocols afford TA-NO_2_ and TA-I in >70% yields and >95% purity across various PET sources. The synthesised TA-NO_2_ and TA-I were subsequently applied as linkers in the synthesis of MOFs UiO-66-NO_2_ and UiO-66-I, which exhibited crystallinity, porosity, and thermal stability comparable to MOFs obtained from commercial linkers. Overall, this work establishes electrophilic substitution-assisted upcycling for wPET valorisation that broadens the chemical space of PET-derived building blocks and delivers clear added value through enhanced functionality and reduced cost.

## Abstract 2

One-step electrophilic upcycling transforms waste PET into 2-nitro and 2-iodoterephtalate linkers for crystalline, porous UiO-66-type MOFs, reducing linker costs by 7–80× while enabling 5–50× performance gains in adsorption and catalysis.

## Graphical abstract

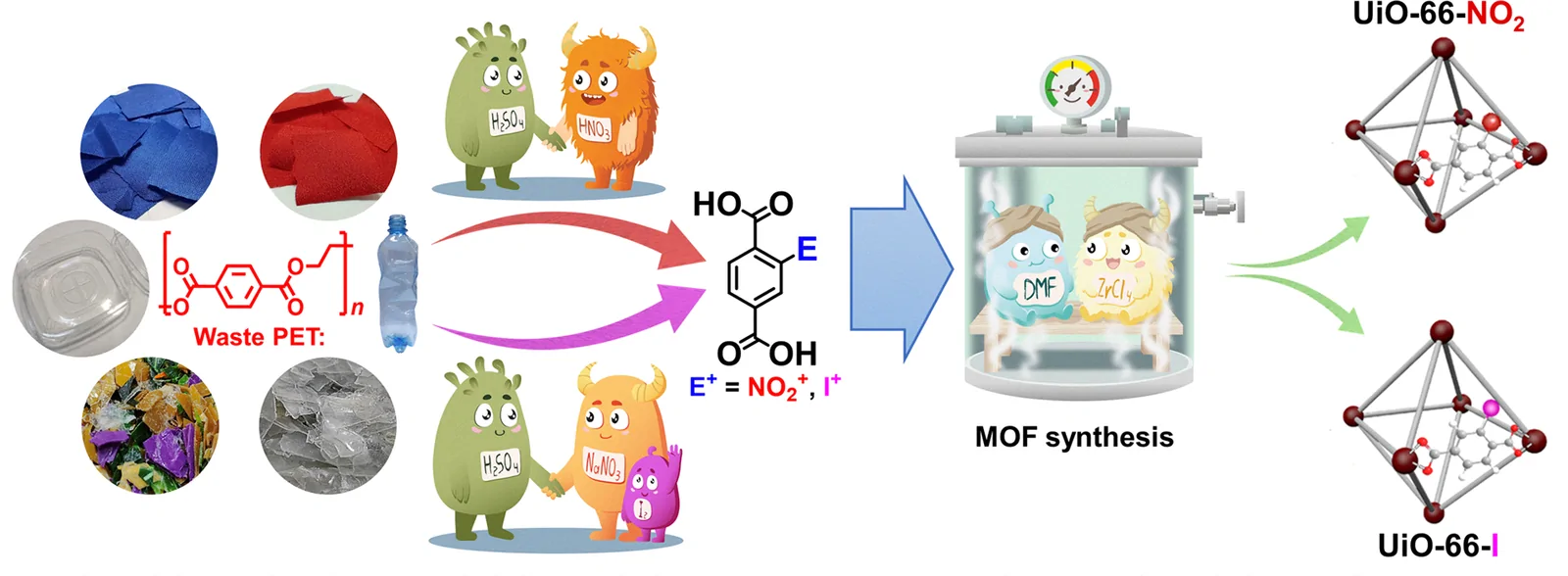

**New concepts**

This work introduces electrophilic substitution-assisted upcycling as a new concept for waste polyethylene terephthalate (wPET) valorisation, enabling access to function-encoded aromatic building blocks that are not reachable by conventional PET upcycling routes. Previously reported chemical upcycling strategies predominantly rely on nucleophilic processes such as aminolysis, hydrazinolysis, methanolysis, or hydrogenolysis, which cleave the polymer backbone to yield terephthalic acid, esters, or amides without modifying the aromatic core. In contrast, the present approach couples depolymerisation with electrophilic aromatic substitution, overcoming the long-assumed inertness of the electron-deficient PET-derived ring and enabling the direct formation of nitro- and iodo-functionalised terephthalic acids. Crucially, this strategy delivers clear added value through two complementary amplification pathways: (i) a performance-driven increase, where linker functionalisation enhances, *e.g.*, adsorption capacity and/or catalytic activity, and (ii) a cost-driven increase, enabled by up to ten times reduction in linker price when sourced from waste PET. Altogether, these effects allow UiO-66-X materials derived from chemical upcycling to outperform their virgin-feedstock analogues both technically and economically. More broadly, this concept establishes waste PET as a chemically addressable platform for materials design, expanding the chemical space of polymer upcycling beyond chain cleavage toward functional and added-value materials.

## 1. Introduction

Among the widely used plastics, polyethylene terephthalate (PET) stands out as one of the most recyclable polymers. However, only about 30% of waste PET (wPET) is recycled annually.^1,2^ Despite progress in mechanical recycling into bottles, construction and packaging materials,^3,4^ side-reactions lead to downcycling with the reduction in molecular weight and intrinsic viscosity (Fig. 1A). Chemical recycling is a promising option in which polymers are broken down into monomers, terephthalic acid (TA), for repolymerisation.^5^ Despite the potential of closed-loop recycling with polymer-to-polymer technologies (Fig. 1A), mechanical and chemical recycling offer limited economic benefit, as the production cost of recycled PET can exceed that of virgin PET, approximately $1.3–1.5 per kg PET compared to the virgin one ($1.1–1.3 per kg PET)^6,7^ (Fig. 1A).

**Fig. 1:**
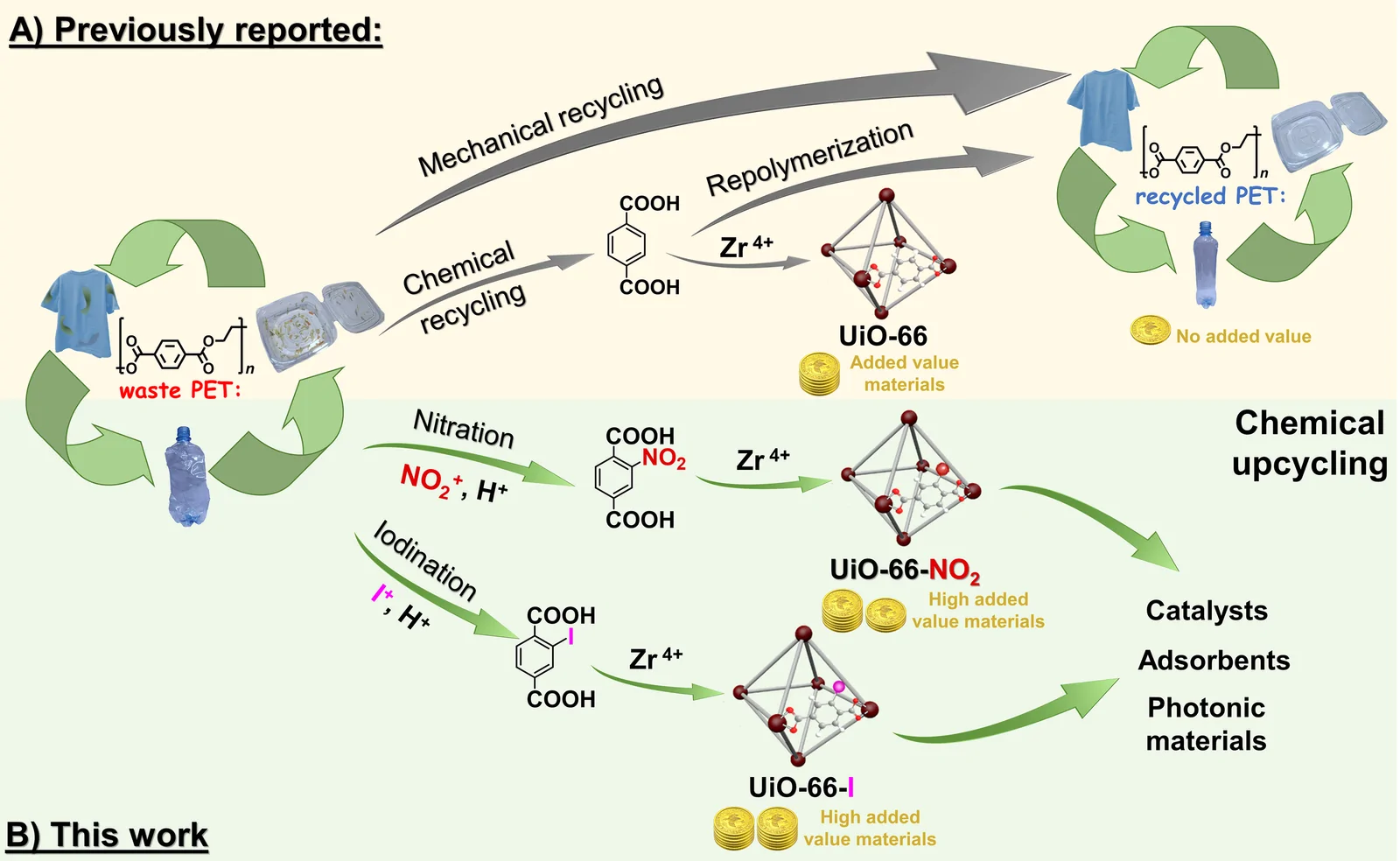
Approaches for wPET recycling and upcycling: (A) previously reported PET recycling followed by repolymerisation or preparation of TA-based MOFs as added value products; (B) PET chemical upcycling to UiO-66-NO_2_ and UiO-66-I with high added value.

In contrast to recycling strategies, upcycling technologies generate new value-added molecules, polymers, or materials through chemical transformations and/or surface functionalisation methods^8,9^ (Fig. 1B). Chemical upcycling of PET has been reported for the conversion to amides and hydrazides *via* aminolysis; it is converted to dimethyl terephthalate *via* methanolysis and hydrogenolysis (Fig. 2A).^10,11^ However, the main focus of the previously reported methods is directed towards the cleavage of the main polymer chain through interaction with appropriate nucleophilic derivatizing carboxylic groups.

**Fig. 2:**
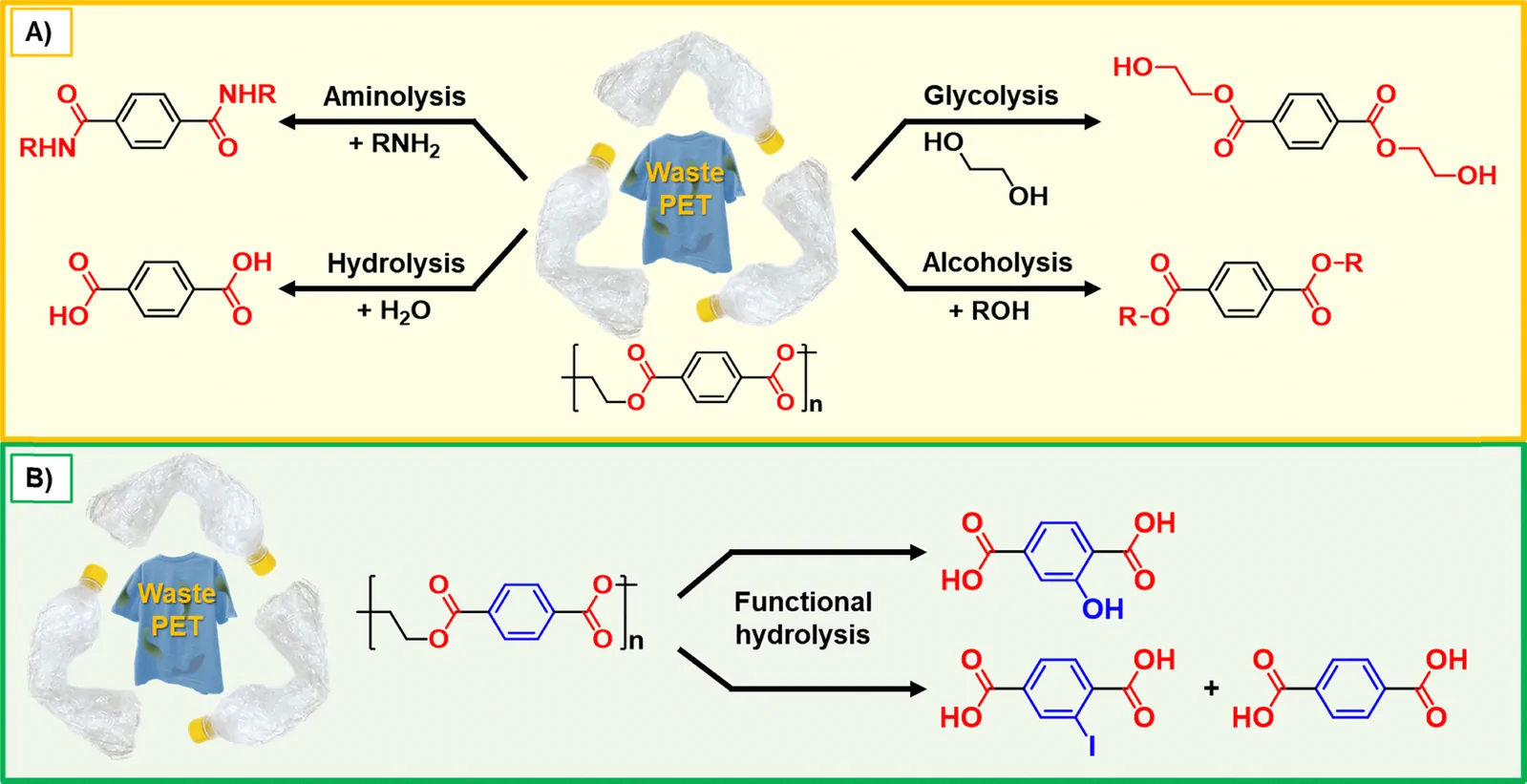
Comparison of classical and novel chemical approaches toward PET upcycling: (A) classical PET upcycling methods: aminolysis,^12^ hydrolysis,^13^ glycolysis,^14^ and alcoholysis.^15^ (B) Emerging PET upcycling methods: hydroxylation^16^ and iodination.^17^

In contrast, electrophilic functionalisation of PET remains largely unexplored, despite its potential to introduce diverse functional groups directly onto the aromatic backbone with new C–X bond formation. This limited application is explained by the electron-poor nature of PET's aromatic rings due to the presence of 2 electron-withdrawing COOR groups, which makes them less reactive toward electrophilic substitution reactions. Moreover, harsh conditions often required for electrophilic aromatic substitution^18,19^ can lead to uncontrolled degradation or low selectivity. To date, only a few examples of successful modification of aromatic rings in PET structure have been reported including our findings (Fig. 2B). Recently, Lim *et al.* demonstrated the first dedicated efforts towards rational functionalisation of the aromatic segment of PET *via* C–H hydroxylation of PET *via* Ru-catalysed oxidation.^16^

Overcoming these challenges would enable the development of novel PET upcycling pathways, expanding the range of high value products that can be derived. The diversification of chemical structures of PET upcycling products plays a pivotal role in the preparation of metal–organic frameworks (MOFs), consisting of TA and metal precursors. Originating from Rajagopal^20^ and De Vos^21^ works, wPET became a source for the preparation of MOFs (Fig. 1, path A), which are actively applied in the fields of energy storage,^22^ catalysis^23^ and environmental remediation.^24^ Nevertheless, the existing PET upcycling strategies generate only TA or TA-based MOFs.^25–27^ The functionality of MOFs can be widened by the utilization of substituted TAs, such as 2-aminoterephthalic acid (TA-NH_2_), 2-nitroterephthalic acid (TA-NO_2_),^28^ 2-halogenterephthalic acid^29,30^ and 2,5-dihalogenterephthalic acid^31,32^ (Fig. 1, path B). For example, the –NH_2_ group provides increased affinity to CO_2_,^33,34^ –the NO_2_ group increases oxygen adsorption^35^ and Lewis acidity,^28^ the –OH group affects gas storage and separation perfomance.^16,36^ Thus, it is opportune to proceed to chemical upcycling to produce costly functionalised TAs, which can provide a higher added value of derived materials due to diversified performance.

In this work, we develop an approach towards wPET upcycling into TA-NO_2_ and 2-iodoterephthalic acid (TA-I) through electrophilic aromatic functionalisation during depolymerisation. wPET from different sources is washed, mechanically shredded (<1 mm), and directly converted *via* one-pot electrophilic functionalisation into value-added terephthalate linkers. After product isolation, the functionalised acids are used directly for MOF synthesis, demonstrating a direct chemical upcycling route from plastic waste to advanced functional materials (Fig. 3, S1).

**Fig. 3:**
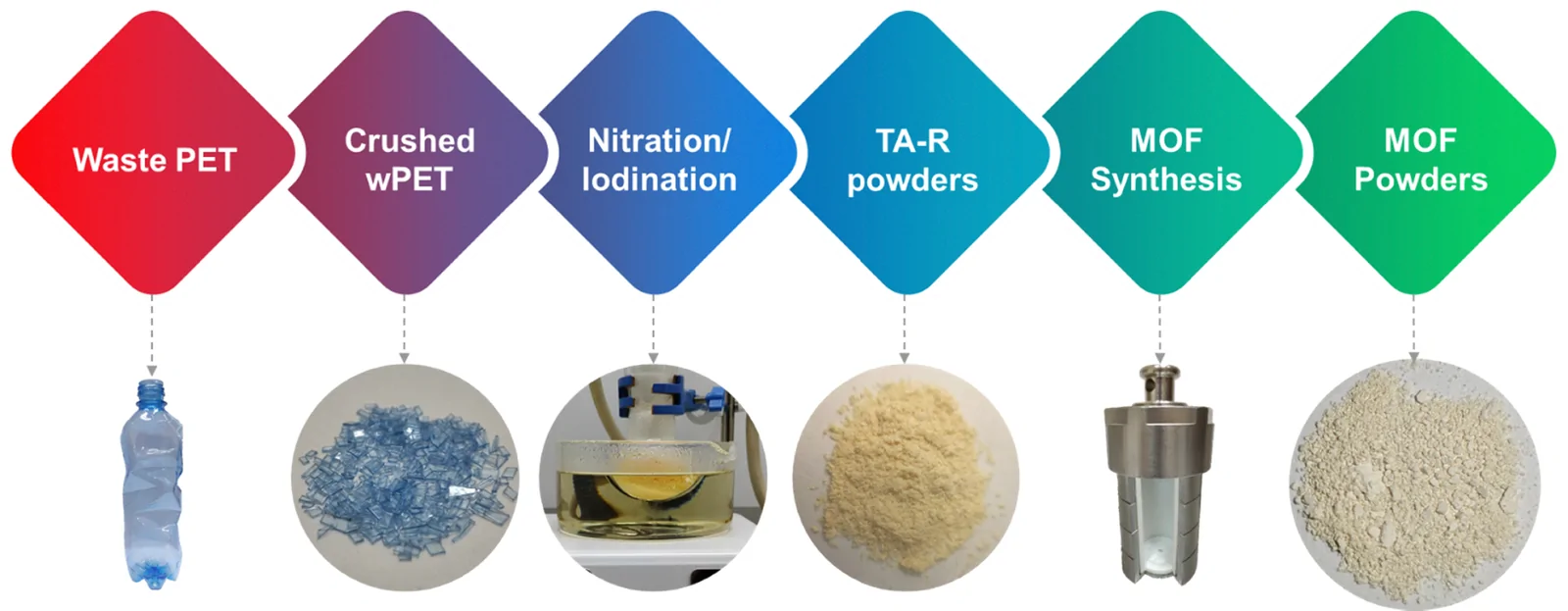
Comprehensive scheme of the PET upcycling process.

These two derivatives were selected because they represent functionally meaningful classes of MOF linkers commonly used in materials design.^37,38^ Nitro substituents employed in UiO-66-NO_2_,^39^ UiO-67-NO_2_^40^ or MIL-53-NO_2_,^41^ systematically adjust the electronic character, polarity, and coordination behaviour of terephthalate frameworks. Iodine substituents introduced in UiO-66-I,^42^ NH_2_-BDC-I,^43^ or halogenated MIL-series linkers^44^ provide a versatile platform for halogen functionalisation, influencing intermolecular interactions, heavy-atom effects, and adsorption of polarizable or halogenated molecules. Altogether, these two examples illustrate the broader design space of functionalised terephthalates, which also includes widely used substituents such as –NH_2_, –Br, –Cl, –F, –CF_3_, –OH, and –SO_3_H in MOF chemistry. These functionalised derivatives were prepared using a range of wPET sources (food containers, bottles, textile PET, and industrial blends), demonstrating the method's robustness and versatility. Furthermore, the synthesised acids were applied as ligands for the preparation of metal–organic frameworks (MOFs). The resulting MOFs exhibited physicochemical properties, such as crystallinity, porosity, and stability, comparable to those obtained from commercially available precursors, thereby confirming the viability of waste-derived reagents in advanced material synthesis.

## 2. Results and discussion

### 2.1. Optimisation of PET upcycling to TA-NO_2_

We commenced our investigations by optimising reaction conditions for the electrophilic functionalisation of wPET. As a model substrate, we chose post-consumer PET boxes (Fig. S2 and S3). The standard technique for the preparation of TA-NO_2_ acid includes the simple nitration of TA by the mixture of HNO_3_/H_2_SO_4_.^45–47^ Moreover, bis(2-hydroxyethyl)terephthalate (BHET) derived from wPET was converted to TA-NO_2_*via* a two-step procedure *via* hydrolysis and nitration.^48^ Therefore, as a starting point, we chose a mixture of HNO_3_/H_2_SO_4_ (1 vol/3 vol) as the commonly used nitration system.^46,47^

The optimisation aims at recovery of TA-NO_2_ from wPET with a high yield along with fulfilling sustainability criteria. Here, we implemented the environmental energy impact factor (EEIF, *ξ*), which directly incorporates the environmental and energetic cost per mass of the isolated product.^10,49^ Although many sustainability metrics exist, such as Sheldon's *E*-factor, PMI, atom economy, neutralisation demand, or NO_*x*_ emission factors,^50^ they mainly quantify waste or mass flows and do not capture the significant energy inputs inherent to PET upcycling. In the context of green chemistry, industrial sustainability, or energy efficiency, this is a more relevant benchmark parameter than commonly used yield or conversion in plastic waste upcycling. EEIF (*ξ*) allows the evaluation of the sustainability and overall resource efficiency of the process more accurately.

The optimisation has been controlled by high-performance liquid chromatography using the peak area of TA-NO_2_. To achieve the minimal EEIF, first, we varied reaction temperature in the 60–110 °C range (Table 1, entries 1–4) to get a balance between energy consumption and time. Second, we varied the molar ratio between HNO_3_/H_2_SO_4_ and wPET to minimise the reaction volume and prevent acid waste formation (Table 1, entries 5–7).

**Table 1:** Optimisation of reaction conditions for wPET to TA-NO_2_ conversion^a^

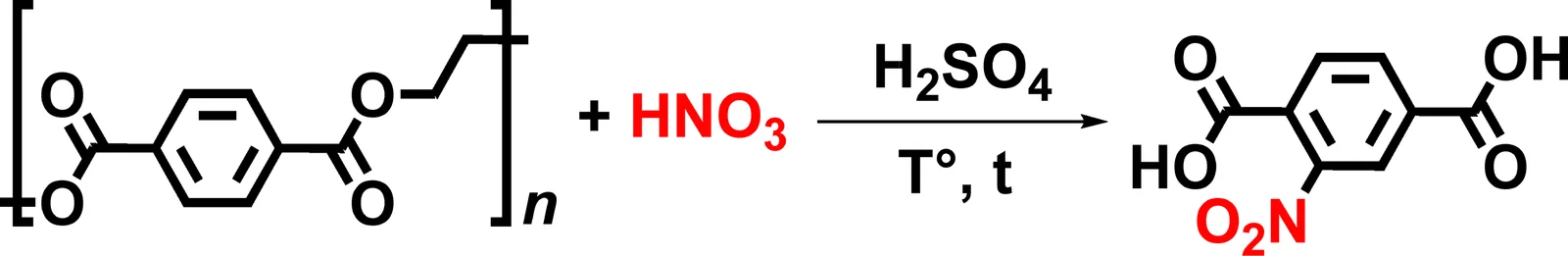					
Entry	Temperature (*T*), °C	Loading of HNO_3_/H_2_SO_4_ (vol/vol)	Time (*t*),^b^ min	Isolated yield, %	EEIF,^c^ × 10^−4^ °C min
1	60	2.5/7.5	4320	74	888.3
2	80	2.5/7.5	360	66	125.5
3	100	2.5/7.5	120	71	45.1
4	110	2.5/7.5	60	68	26.8
5	100	1.8/8.2	120	52	42.4
6	100	1.1/8.9	360	42	166.5
7^d^	100	1.8/5.4	120	87	23.8

a Reaction conditions: 2 g (10.4 mmol) reacted with the HNO_3_/H_2_SO_4_ mixture according to the experimental procedure.

b Reaction time has been determined by full conversion of PET according to HPLC data.

c EEIF incorporates the mass of reagents, temperature, and time per unit mass of product (see eqn (S1) in the SI).

d Optimal reaction conditions.

#### 2.1.1. Temperature

Firstly, we aim at variation of reaction temperature to minimise energy consumption and achieve high TA-NO_2_ yield (Table 1, entries 1–4). wPET nitration at 60 °C (Table 1, entry 1) reaches the full conversion of PET after 4320 minutes. Despite relatively low temperature, the reaction was time-consuming, therefore, giving high EEIF (*ξ* = 888.3 × 10^4^ °C min). Elevating the temperature to 80 °C led to a 7-fold decrease in EEIF to 125.5 × 10^4^ °C min. The further increase to 100 °C led to the apparent shortening of reaction time to 120 minutes with subsequent decrease in EEIF (*ξ* = 45.1 × 10^4^ °C min). Indeed, the following elevation of temperature to 110 °C allowed achieving the full conversion in 60 minutes resulting in the lowest EEIF (*ξ* = 26.8 × 10^4^ °C min).

Despite low EEIF, these conditions are considered to be not optimal for technological and safety reasons. The addition of crushed PET to the reaction mixture led to uncontrollable boiling and blowout of reaction media to the reflux condenser (Fig. S4 and the accompanying movie). Taking into account the potential realization in the real technological process, we decided to stop the experiments at temperatures higher than 110 °C due to the safety reasons. Therefore, 100 °C has been considered as an optimal regime for the further experiments (Fig. 4A).

**Fig. 4:**
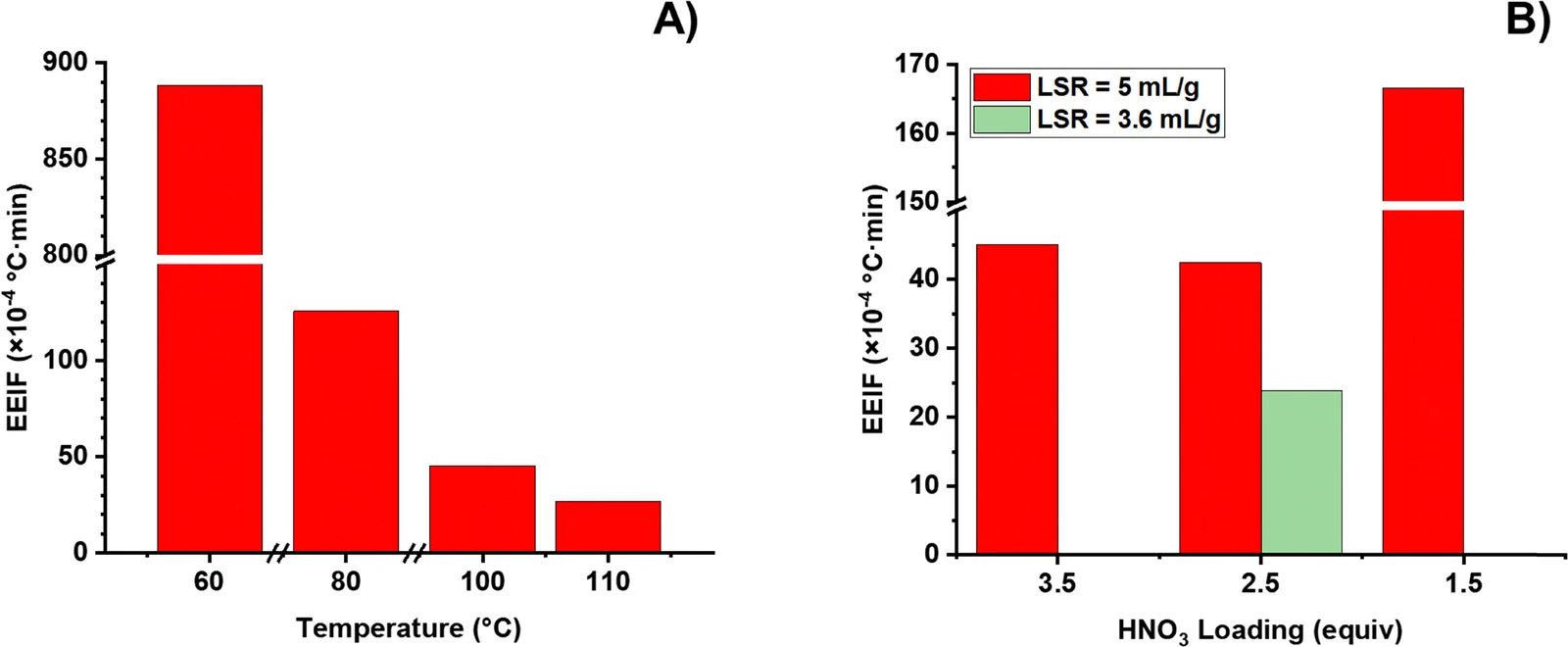
EEIF dependence on reaction conditions: (A) temperature; (B) acid loadings (LSR – liquid/solid ratio = HNO_3_/H_2_SO_4_ mixture/PET ratio, mL g^−1^), the mixture of 1.8 mL of HNO_3_ and 5.4 mL of H_2_SO_4_ has been taken for LSR 3.6.

#### 2.1.2. Loading of acids

After choosing the optimal temperature of 100 °C, we aimed at decreasing acid amount. Fig. 4B demonstrates the dependence of the EEIF on the equivalents of HNO_3_ with nitric mixture/wPET ratio (liquid/solid ratio = LSR) of 5 mL g^−1^ (orange bar) and 3.6 mL g^−1^ (green bar). The decrease of HNO_3_ loading to 1.8 mL (2.5 equiv.) instead of 2.5 mL (3.5 equiv.) used in previous experiments (Table 1, entry 5) led to quantitative conversion in 2 hours with slightly decreased EEIF (*ξ* = 42.4 × 10^4^ °C min) compared to 45.1 × 10^4^ °C min. The further decrease of HNO_3_ loading to 1.1 mL (1.5 equiv.) (Table 1, entry 6) led to the prolongation of reaction time up to 6 hours with a drastic increase in EEIF (*ξ* = 166.5 × 10^4^ °C min). Finally, the mixture of 1.8 mL of HNO_3_ and 5.4 mL of H_2_SO_4_ with LSR = 3.6 mL g^−1^ (Table 1, entry 7, Fig. 4B) afforded a minimum EEIF of 23.8 × 10^4^ °C min with full wPET conversion in 2 hours (Fig. 5A), which was chosen as the optimal reaction time. Thus, experimental conditions in Table 1, entry 7 have been considered as optimal, affording pure TA-NO_2_ after common work-up with 87% yield and >95% purity according to NMR and HPLC results (Fig. 5B, S5). These purity results are comparable with commercial TA-NO_2_ (Fig. S6). Unlike previously reported multistep routes that required prior depolymerisation to terephthalic acid or bis-(2-hydroxyethyl) terephthalate intermediates,^48^ our approach integrates depolymerisation and electrophilic substitution into a single step, thereby reducing reagent consumption, reaction time, and waste generation.

**Fig. 5:**
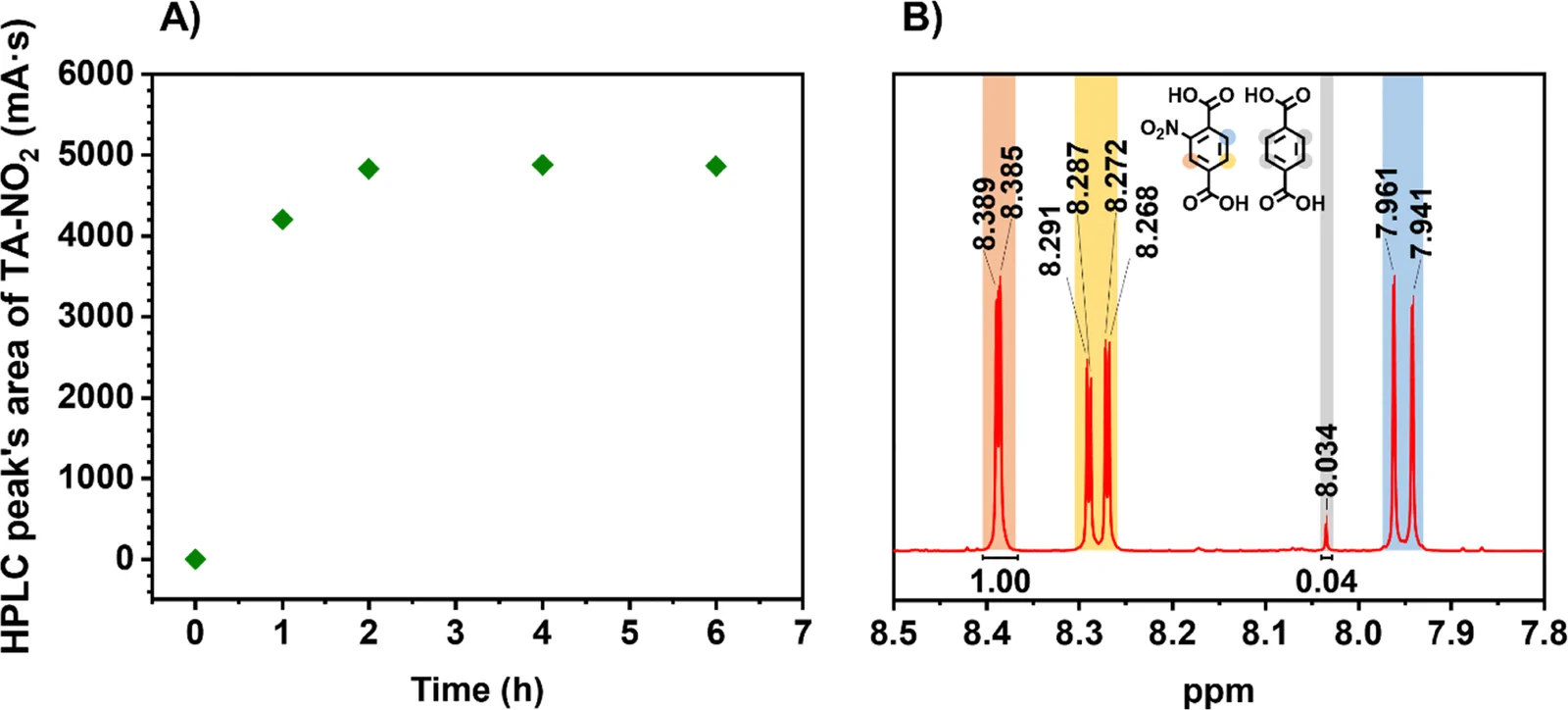
Optimisation of time of reaction: (A) TA-NO_2_ HPLC peak dynamic under optimal conditions (entry 7, 100 °C, LSR = 3.6, 2.5 HNO_3_ eq.); (B) NMR spectrum of isolated TA-NO_2_.

### 2.2. Upcycling of different wPET sources into TA-NO_2_

After optimisation of upcycling wPET from food containers (Fig. 6A), we probed this procedure on diverse sources of wPET. Thus, we tested the industrial mixture of coloured PET (Fig. 6B) containing approx. 5% of PE (see Section S1, eqn (S1)), colourless wPET from the GAIKER company (Fig. 6C), samples of polyester textile from dyed fabric – blue one (Fig. 6D) and red one (Fig. 6E). All these samples are specifically challenging because of the previously reported slower recycling rate of coloured or blended PET.^51,52^

**Fig. 6:**
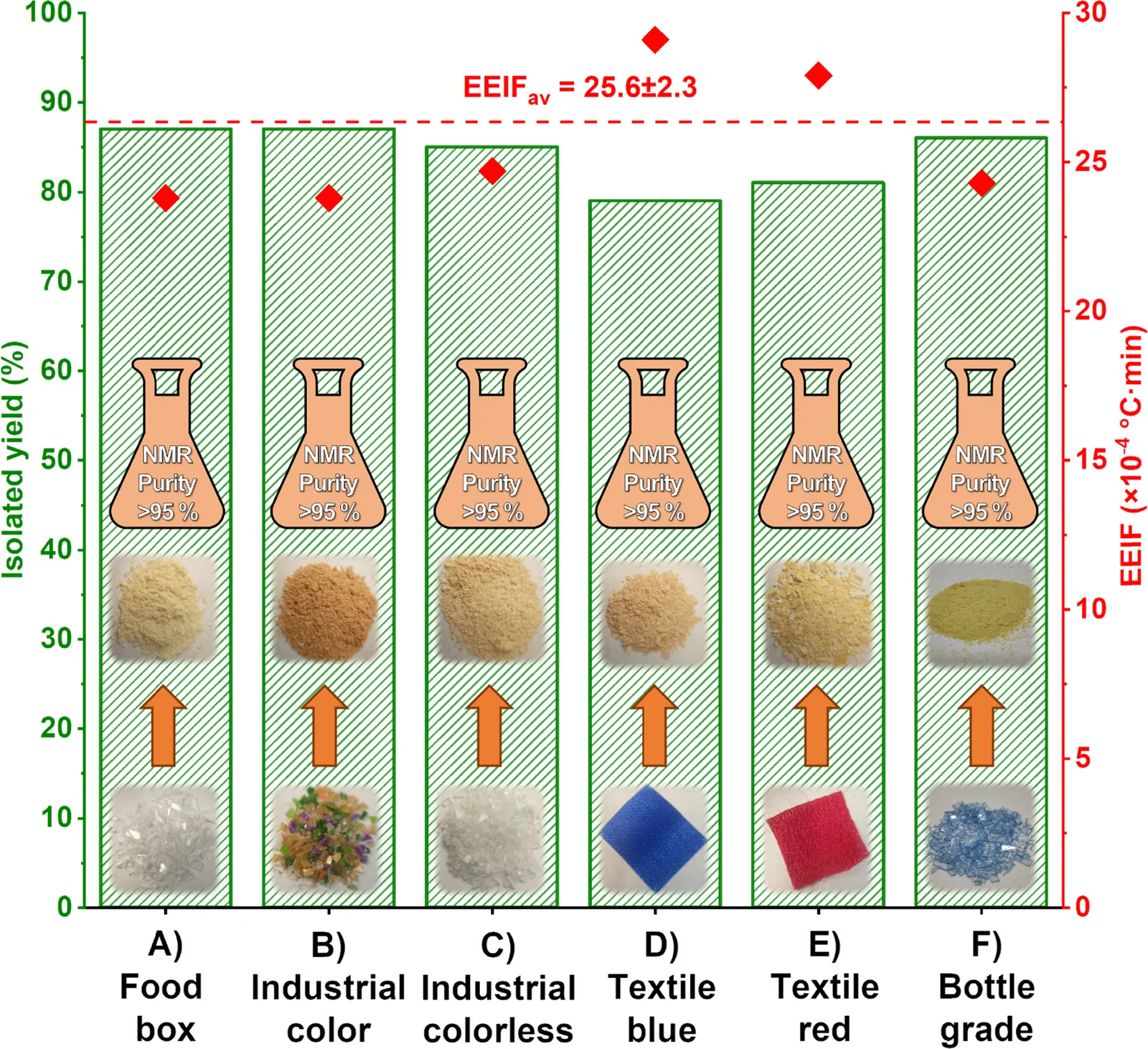
Chemical upcycling of (A) wPET food boxes, (B) coloured industrial wPET with PE (∼5%) impurities; (C) colourless industrial wPET; (D) textile wPET blue and (E) textile wPET red; (F) bottle grade wPET. The violet line shows the average value of the EEIF coefficient.

To validate the established optimal upcycling conditions for industrial and textile wPET, the EEIF and yield have been determined as benchmark parameters. Specifically, the optimal reaction time was identified as 2 hours for all samples based on HPLC data followed by the calculation of the EEIF. The developed approach demonstrated close-to-perfect reproducibility independently on waste sources (Fig. 6). Thus, for industrial coloured wPET, an EEIF of 23.8 × 10^4^ °C min was achieved with complete wPET conversion in 2 hours. Similar results of EEIF were also obtained for other samples (colourless industrial wPET *ξ* = 24.7° × 10^4^ °C min; blue textile wPET *ξ* = 29.1° × 10^4^ °C min; red textile wPET *ξ* = 27.9° × 10^4^ °C min).

However, different types of polymeric waste exhibit a broad range of molecular weights and characteristics, reflecting variations in feedstock composition, processing history, and extent of material degradation.^53^ Given that intrinsic viscosity correlates with average molecular weight and supramolecular organization, variations in intrinsic viscosity may significantly affect the accessibility of reactive sites to the nitrating agent and, consequently, the rate of the heterogeneous reaction. Polymers with higher intrinsic viscosity values are anticipated to require extended reaction times to achieve comparable product yields. To assess the limiting kinetic case, we intentionally included the sample with the highest characteristic viscosity—bottle grade PET waste (Fig. 6F), which was used in our previous study.^17^ The EEIF value for bottle grade wPET (*ξ* = 24.3 × 10^4^ °C min) remained in the same range. These minor fluctuations may arise due to slight differences in product yield; however, these variations are expected to be minimised upon further optimisation of the product isolation procedure.

To eliminate the influence of intrinsic viscosity of different wPET types on the reaction rate we performed kinetic experiments with all wPET sources (see Section S3.2, Fig. S7). The experimental results demonstrate that the initial rate of TA-NO_2_ formation for the investigated samples ranges from 0.0156 to 0.0188 mol L^−1^ s^−1^. Consistent with expectations, bottle grade PET exhibited the lowest initial reaction rate. Nevertheless, under optimised nitration conditions, all waste types, including relatively tough bottle grade material, afforded comparable final product yields of 86% (Fig. 6F). This finding confirms the robustness and applicability of the proposed methodology for the chemical upcycling of heterogeneous PET waste streams.

The non-polymeric impurities of wPET could affect the yield and quality of upcycled products.^51,52^ Applying the developed upcycling procedure to all sources, we isolated TA-NO_2_ with >95% purity based on ^1^H NMR spectra and HPLC chromatograms (Fig. S8 and S9) for all samples with 79–87% yield. The prepared samples have been used for the further preparation of MOFs without additional purification.

However, slight variations in the product colour raised concerns regarding the potential presence of residual dyes originating from the initial waste. To address this, additional spectroscopic analysis was performed in the UV and visible regions. The absorption spectra of the products were compared with those of pure TA-NO_2_ (Fig. S10), confirming the absence of coloured impurities.

For mixed waste sample B, the proposed upcycling provides the possibility to isolate PE impurities with quantitative yields. The purity of isolated PE proved by FTIR-spectroscopy confirms a close-to-perfect match to commercial high density PE samples (Fig. S11). In the case of upscaling developed PET/PE upcycling, isolated PE is applicable to further processing.^54,55^

Thus, optimal experimental conditions have been tested for upcycling of different wPET sources affording an average EEIF value of 25.6 ± 2.3 × 10^4^ °C min and isolation of pure TA-NO_2_ after common work-up with 79–87% yield and >95% purity according to NMR, HPLC and UV-vis spectroscopy.

We note, however, that the use of HNO_3_/H_2_SO_4_ in these electrophilic substitution reactions generates NO_*x*_. While these emissions are not fully captured by EEIF, the overall sustainability of the process is supported by several factors: (i) the stoichiometric excess of nitrate relative to product mass is low; (ii) the reactions are performed at controlled temperatures where NO_*x*_ evolution is minimised; and (iii) in the case of upscaling, NO_*x*_ management could rely on well-established industrial technologies. For industrial-scale application, NO_*x*_ emissions can be effectively managed using established scrubbers (*e.g.*, wet or alkaline) or oxidation/reduction methods, which convert nitrogen oxides into recyclable nitrate/nitrite streams.^56^ Integrating such NO_*x*_-capture units, together with acid recycling, would be straightforward in future scale-up and ensures compliance with industrial environmental and safety standards.

### 2.3. Optimisation of upcycling of industrial PET to TA-I

In addition to TA-NO_2_ optimisation, we aimed to recover another MOF-relevant linker – TA-I being 10 times more expensive compared to TA. TA-I is a promising linker for the preparation of metal–organic frameworks, especially those involving halogen bonding^17,57,58^ or coordination to heavy metals.^59,60^ A four-step synthesis of TA-I is commonly employed, consisting of nitration, reduction of NO_2_ group, diazotisation, and iodination.^61^ While this approach affords high yields and product purity, its multistep nature and use of expensive reagents significantly reduce its economic viability. Alternatively, direct iodination of TA using an iridium-based catalytic system has been reported, although this method is synthetically demanding and offers only moderate efficiency (52% yield).^62^ As a result, the overall production cost of TA-I significantly exceeds that of terephthalic acid, making the conversion of wPET into TA-I a highly attractive and valuable upcycling route.

As a starting point, we used a previously developed protocol affording a 1 : 1 mixture of TA and TA-I,^17^ which served as precursors for MOF synthesis. We aimed at optimisation of >95% NMR purity TA-I. The PET source used for these experiments was an industrial PET/PE blend (Fig. 6B) identical to that employed in the nitration experiments described in the SI (see Section S1.2.3). Similar to the nitration-based upcycling route, the optimisation was guided by the EEIF, *ξ*. To minimise *ξ*, we investigated the effects of reaction temperature, nitrate source, and molar ratio between PET and the NO_3_^−^ donor (Table 2, entries 1–7), while maintaining a constant reaction time of 24 hours across all experiments.

**Table 2:** Optimisation of reaction conditions for wPET to TA-I conversion^a^

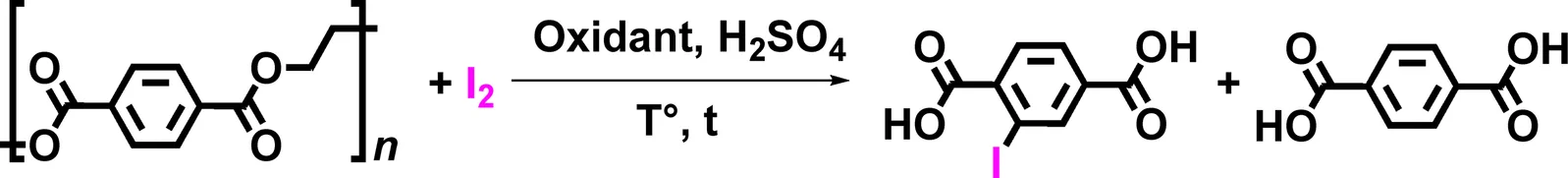						
Entry	Temperature, °C	Oxidant	Oxidant loading, equiv.	TA-I/TA ratio, mol%/mol%	Yield, %	EEIF,^b^ × 10^−4^ °C min
1	70	HNO_3_	5.5	76/24	37	1879.7
2	60		5.5	88/12	42	1237.1
3	70	NaNO_3_	5.5	81/19	64	713.3
4	60		5.5	93/7	56	753.9
5	50		5.5	94/6	56	609.3
6^c^			4	95/5	72	354.3
7			3	89/11	66	418.7

a Reaction conditions: 2 g (10.4 mmol) of crushed PET reacted with HNO_3_ (or NaNO_3_), H_2_SO_4_ 15.60 mL, (287 mmol) and 4 g (15.75 mmol) of I_2_ for 24 hours.

b EEIF.

c Optimal reaction conditions.

#### 2.3.1. Effects of temperature and oxidant

Initially, we examined the temperature effect on TA-I purity and yield using nitric acid (HNO_3_) as an oxidant for generation of electrophilic iodine “I^+^” species (Fig. 7, Table 2, entries 1 and 2). The reaction at 70 °C was distinguished by poor selectivity and low yield (Table 2, entry 1), resulting in a high EEIF (*ξ* = 1879.7 × 10^4^ °C min). Lowering the temperature to 60 °C increased product yield and selectivity of TA-I formation affording an EEIF of 1237.1 × 10^4^ °C min (Table 2, entry 2). Nevertheless, product yield of 42% and purity of 88% remained insufficient, likely due to enhanced hydrolysis and side nitration processes at elevated acid concentrations.

**Fig. 7:**
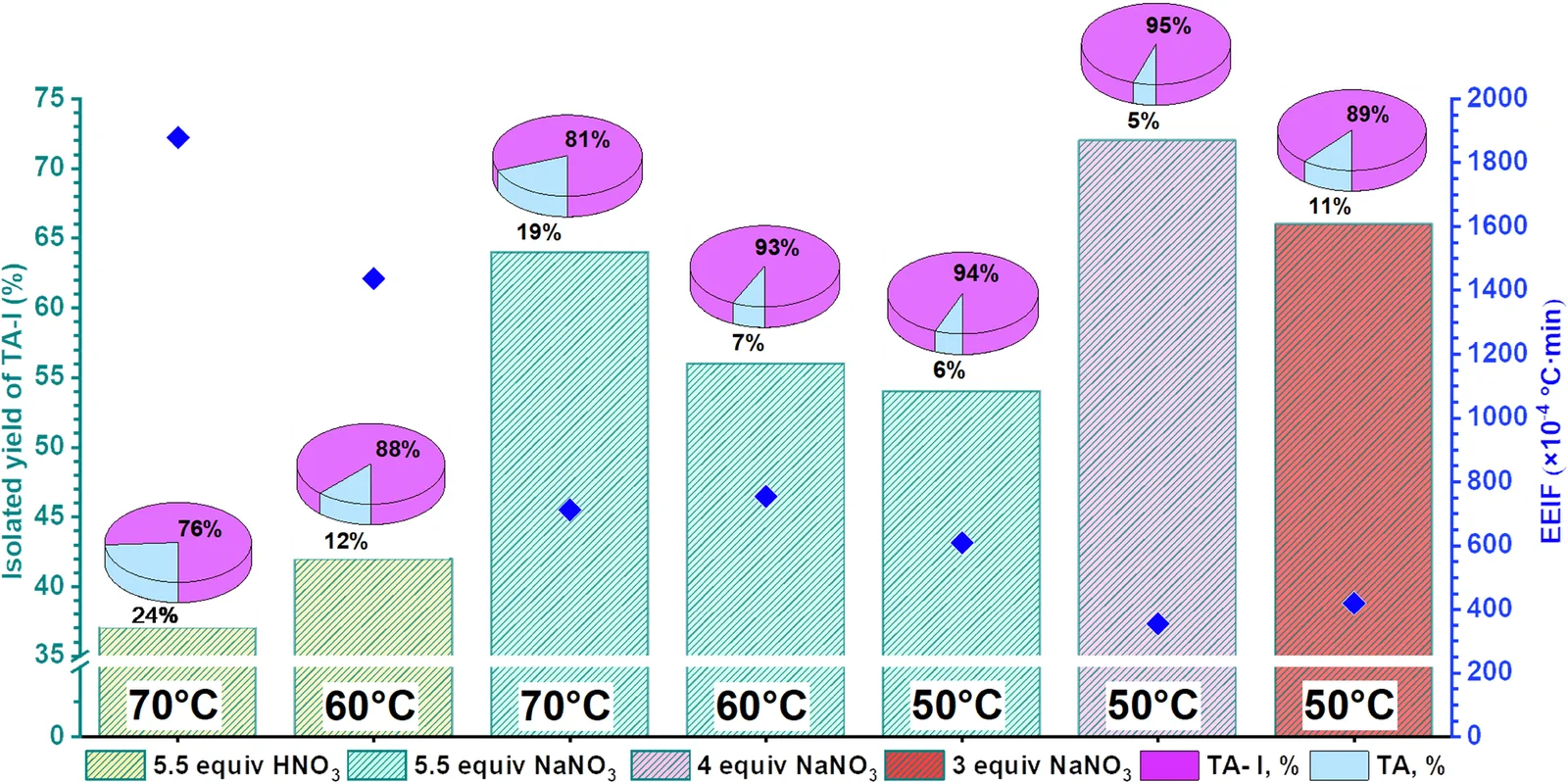
Optimisation of upcycling of wPET to 2-iodoterephthalic acid (TA-I). The bar plots represent isolated yield (%) of TA-I at different temperatures, oxidants (HNO_3_ or NaNO_3_), and oxidant loadings (3–5.5 equiv.); the blue diamonds indicate the corresponding EEIF, *ξ*; 10^−4^ °C min, the circle diagram presents the mol% of TA-I (purple) and TA (cyan) in products.

To mitigate these issues, HNO_3_ was replaced with sodium nitrate (NaNO_3_) as an oxidant (Fig. 7, Table 2, entries 3–5).^63^ Under otherwise identical conditions (Table 2, entries 3 and 4), this substitution led to a significant improvement in yield, selectivity, and purity, along with a 2.5-fold reduction in *ξ* values (713.3 × 10^4^ °C min at 70 °C; 753.9 × 10^4^ °C min at 60 °C). Further decrease of the temperature to 50 °C increased product purity and reduced the EEIF to 609.3 × 10^4^ °C min (Table 2, entry 5), although TA-I yield slightly decreased. Based on these results, the combination of NaNO_3_ and 50 °C was selected as the optimal condition for further experiments.

#### 2.3.2. Effect of NaNO_3_ loading

Following the identification of 50 °C as the optimal temperature and NaNO_3_ as an oxidant, we next investigated the effect of sodium nitrate loading on the reaction yield and selectivity (Table 2, entries 6 and 7). Lowering the NaNO_3_ amount from 5.5 equiv. (Table 2, entry 5) to 4.0 equiv. (Table 2, entry 6) resulted in a higher yield of TA-I, accompanied by a minor decrease in the TA-I/TA ratio. This adjustment significantly lowered the EEIF to 354.3 × 10^4^ °C min. The implementation of decreased NaNO_3_ amount leads to a lower EEIF, as the overall reagent mass is directly reduced (see equation (S1), Section S1.2.1). At the same time, a lower concentration of nitrate prevents backside nitration of the PET backbone, increasing the selectivity of reaction towards formation of TA-I. However, further reduction of NaNO_3_ to 3.0 equiv. (Table 2, entry 7) led to a pronounced decrease in product yield and TA-I selectivity resulting in an increased EEIF of 418.7 × 10^4^ °C min. Thus, the conditions corresponding to entry 6 were identified as optimal, affording 2-iodoterephthalic acid in 72% isolated yield and >95% purity according to NMR and HPLC after standard work-up (Fig. 7, S12–S14). The isolated product has been used for the further preparation of MOFs without additional purification.

### 2.4. Comparison with a conventional multi-step synthesis

To evaluate the technological advantage of direct electrophilic upcycling, we compared the proposed PET-to-TA-I process with a conventional multi-step synthetic pathway reported in the literature (Fig. 8).

**Fig. 8:**
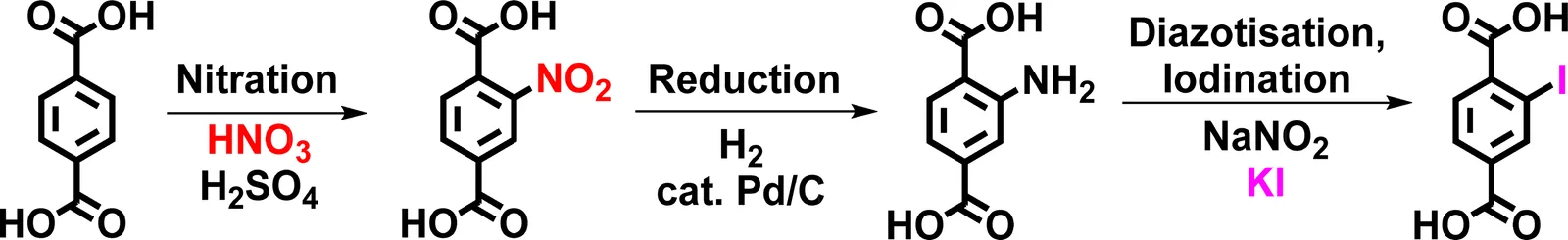
Multi-step preparation of TA-I from TA.^61,64,65^

To the best of our knowledge, there are no published patents of TA-I preparation, which the industry could utilize for large-scale production. However, from previous reports^61,66,67^ and general consideration, we hypothesize the following industrial pathways, namely, the nitration, reduction, diazotisation and iodination sequence. The industrial pathway originates from TA (Fig. 8), which can be synthesised either by the petroleum-based method (catalytic oxidation of *p*-xylene^68^) or chemical recycling of waste PET (Fig. 2A). The nitration of TA proceeded under heating at 90 °C in an oleum/HNO_3_ mixture.^64^ The prepared 2-nitroterephthalic acid was further reduced by H_2_ in alkaline solution in the presence of Pd/C to get 2-aminoterephthalic acid with quantitative yield.^65^ In the third stage, the amino group underwent diazotisation-iodination using sodium nitrite followed by the addition of KI to get target TA-I.^61^

Using the same EEIF definition employed throughout this work, the overall EEIF of the conventional route was estimated from the reported reaction conditions and stoichiometric reagent consumption (see Section S4, Tables S1–S3). The calculated EEIF value reached 357.2 × 10^4^ °C min, comparable to the value obtained for the direct PET recycling method under optimised conditions (354.3 × 10^4^ °C min). However, the advantage of our method is the reduction of the sequence of three synthetic transformations to one. It eliminates intermediate isolation and purification steps, avoids the use of potentially explosive H_2_, and substantially simplifies process design, reducing equipment requirements, operational costs, and overall manufacturing complexity.

### 2.5. Mechanistic consideration of PET nitration/iodination

Both proposed processes proceed according to the common electrophilic substitution pathway and differ only by the generated electrophilic species (Fig. 9A and B).

**Fig. 9:**
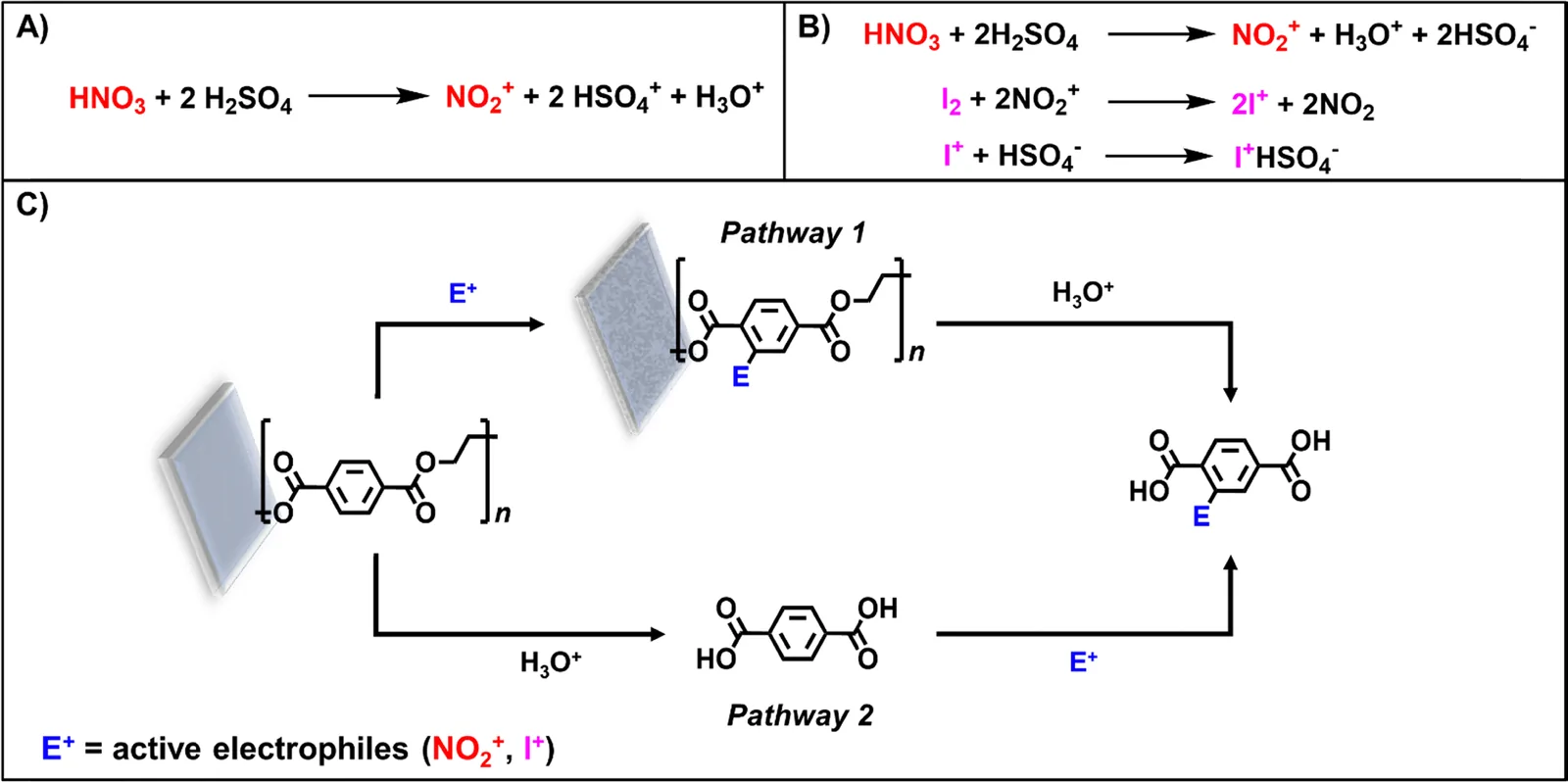
Generation of active electrophiles: (A) nitronium cation NO_2_^+^; (B) I^+^ species and (C) proposed reaction pathways for PET nitration/iodination.

In the case of nitration, the active nitronium cation NO_2_^+^ is formed *via* interaction of HNO_3_ with H_2_SO_4_ with subsequent attack of the benzene ring (Fig. 9A).^69^ The Tronov–Novikov iodination proceeds in a more complex way (Fig. 9B) as was well-documented previously.^70–73^ The generated nitronium cation as a strong oxidant interacts with iodine forming I^+^ species representing the mixture of polyiodide cations stabilized by sulfate.^74,75^ The resulting electrophilic species are able to interact with electron-poor aromatic rings including terephthalate moieties. Thus, the fundamental basis of functional upcycling is established previously.

The most exciting point is the heterogenous character of the process. Hypothetically, the reaction of PET in strong acids can proceed in two major pathways: pathway 1 – hydrolysis of PET with the formation of TA followed by interaction with electrophile, and pathway 2 – the electrophilic attack on the surface of PET followed by hydrolysis (Fig. 9C). The introduction of electron-withdrawing groups (such as –I or –NO_2_) is able to decrease the electron density on the carbon atom of the C=O group, facilitating further hydrolysis. Thus, the elucidation of the preferential pathway deserves attention.

To verify the fundamental chemical feasibility of both routes, preliminary control experiments were performed. Nitration of commercial TA under optimised conditions afforded mononitrated TA-NO_2_ in high purity (Fig. S15A), confirming the viability of pathway 2. In parallel, exposure of a solid PET film to the nitrating mixture resulted in partial surface-level nitration, as evidenced by the appearance of characteristic nitro-group bands in the FTIR spectrum ∼1540 cm^−1^ (*asym* NO_2_ str vib) and ∼1270 cm^−1^ (*sym* NO_2_ vib) without significant disruption of the polymer backbone (Fig. S15B). These results confirm that pathway 1 is also possible.

However, to conclusively discriminate between these two routes and identify the predominant mechanism, a comparative kinetic study in a competitive mode is required. For this purpose, an equimolar mixture of BHET (serving as a soluble molecular model for the PET unit) and pure TA was subjected to nitration. The reaction progress was monitored over time by HPLC analysis to construct kinetic profiles for the consumption of reactants and the formation of the corresponding TA-NO_2_ (Fig. 10A, S16).

**Fig. 10:**
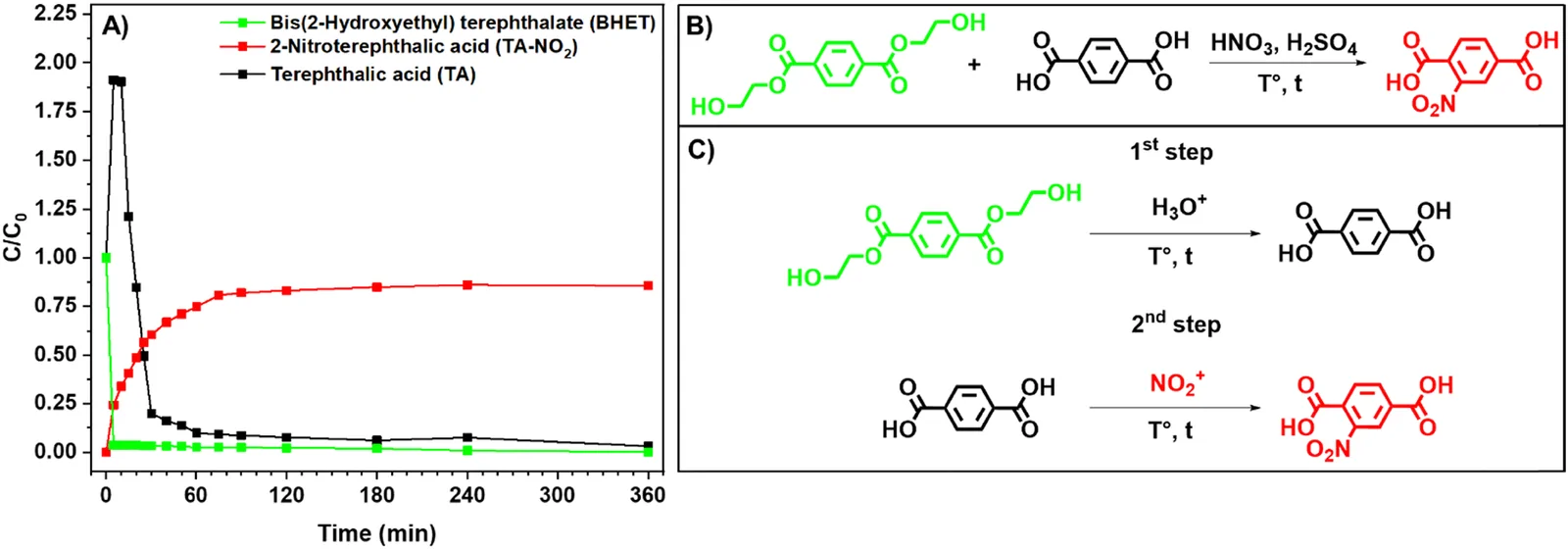
Nitration of an equimolar mixture of TA and BHET: (A) kinetic profiles of TA, BHET and TA-NO_2_; (B) overall reaction scheme, (C) stepwise reaction scheme.

Already within the first 5 minutes of the reaction, the concentration of BHET decreases by 98%, the concentration of TA increases by 90%, and the concentration of the product TA-NO_2_ increases to a value corresponding to the difference between the consumed BHET and the formed TA. These data allow several important conclusions. First, the nearly complete disappearance of BHET accompanied by a sharp increase in TA concentration indicates that the hydrolysis of BHET to TA proceeds significantly faster than its electrophilic nitration (Fig. 10B and C). Second, the fact that the TA content increase (90%) is slightly lower than the BHET consumption (98%) indicates that a portion of the newly formed TA has already reacted with NO_2_^+^ to yield TA-NO_2_.

Thus, the obtained kinetic data demonstrate that under nitration conditions, hydrolysis proceeds significantly faster than the nitration of free TA. This means that the overall process is limited by the rate of TA nitration in solution, *i.e.*, pathway 2 predominates: initial hydrolysis of PET to free TA, followed by its electrophilic nitration.

This conclusion does not contradict with literature data demonstrating that PET nitro-derivatives hydrolyse more readily than pristine PET.^76^ Even if direct surface nitration of PET occurs to some extent (pathway 1), the resulting nitrated polymer fragments, due to the increased lability of the ester bonds, are instantaneously hydrolysed to yield TA-NO_2_. Thus, both pathways led to the same TA-NO_2_, but the sequential “hydrolysis → nitration” scheme (pathway 2) provides the major contribution.

Generally, the mechanism of electrophilic iodination is similar to the nitration process. The main difference is in the generation of the active species attacking the benzene ring. The reaction is initiated by activation of molecular iodine in the strongly acidic HNO_3_/H_2_SO_4_ medium. According to previous studies of aromatic iodination, oxidation of I_2_ under strongly acidic conditions generates highly electrophilic iodine-containing species capable of electrophilic aromatic substitution. These activated iodine species are frequently represented as I^+^-equivalent electrophiles in mechanistic schemes.^73,77,78^ The formation of such electrophilic iodine species has been discussed previously (Fig. 9B).^77^

While positively charged iodine species have been proposed in strongly acidic media,^78^ direct spectroscopic identification of a discrete free I^+^ species remains challenging. Subsequent spectroscopic investigations demonstrated that several species previously assigned as I^+^ are more accurately described as polyiodine cations, including I^2+^, I^3+^, and I^5+^.^73^ Therefore, Fig. 9B should be regarded as a mechanistic representation of electrophilic iodine activation rather than direct evidence for a uniquely identified iodinating intermediate.

### 2.6. Synthesis and characterization of UiO-66-X

Isolated TA-R without additional purification was involved in the preparation of UiO-66-X according to the previously described procedure.^17,79^ UiO-66-I and UiO-66-NO_2_ have been prepared by mixing ZrCl_4_ and TA-R solutions in DMF followed by heating in a sealed autoclave at 80 °C for 24 h with subsequent removal of solvent and activation under vacuum. Prepared UiO-66-X was characterised by XRD, TGA, FTIR spectra, surface area measurements within BET theory and digested according to the reported procedure^80^ to determine the TA/TA-R ratio and presence of residual solvent after synthesis.

The FTIR spectrum of UiO-66-I and UiO-66-NO_2_ synthesised from the upcycled PET demonstrated characteristic vibration bands associated with COO^−^ (*asym* str ∼ 1583 cm^−1^, *sym* str ∼ 1387 cm^−1^), COO–Zr groups (∼1387 cm^−1^) and the C=C vibration of the substituted benzene ring at (∼1480 cm^−1^) for both MOFs (Fig. 11A). Vibrations at lower frequencies (∼658 cm^−1^ and ∼760 cm^−1^) assigned to Zr–O and C–I vibrations are also typical of UiO-66-I. The characteristic vibrations of UiO-66-NO_2_ are at ∼1250 and ∼1550 cm^−1^.

**Fig. 11:**
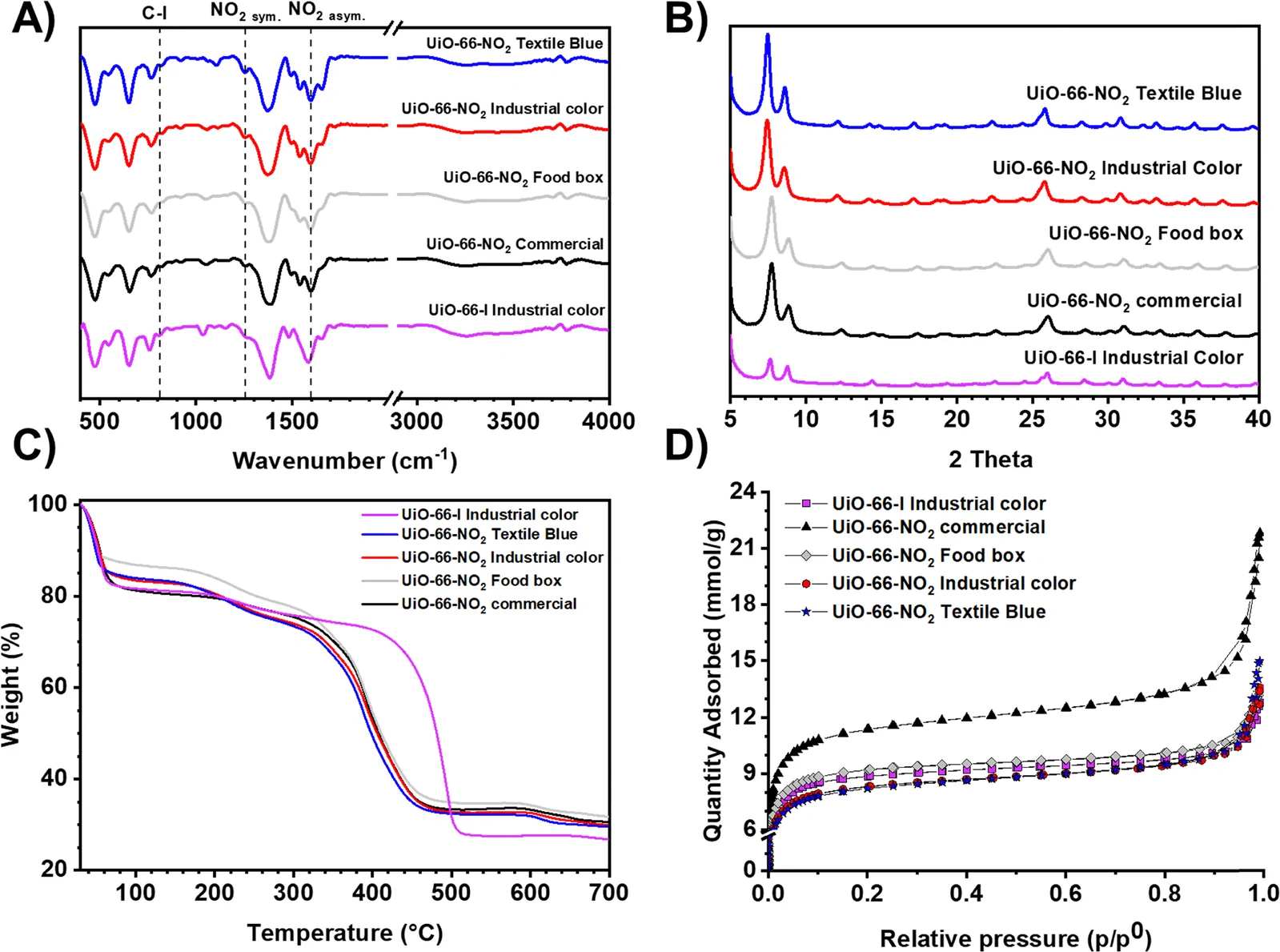
Characterization of UiO-66-R prepared from wPET. (A) FTIR spectra; (B) XRD pattern; (C) TGA curve and; (D) isotherms of N_2_ adsorption–desorption.

The crystallinity of UiO-66-I and UiO-66-NO_2_ was verified by XRD measurements demonstrating a similar diffraction pattern with previously reported cases being isostructural to the pristine UiO-66 (Fig. 11B). The powder XRD patterns of all five samples (UiO-66-NO_2_ from three wPET sources, UiO-66-NO_2_ from commercial TA-NO_2_, and UiO-66-I from Industrial coloured) are in good agreement with the simulated UiO-66-type **fcu** topology, showing the characteristic reflections at 7.4 (7.7)° and 8.8 (8.6)° (2*θ*).^38,66^ The PXRD patterns of all samples match the UiO-66 **fcu** topology, with no additional peaks from crystalline impurities, confirming high phase purity. Differences in crystallite order were evaluated from the width of the (111) reflection at 7.4° (2*θ*), since peak intensity alone is not a reliable measure of crystallinity. Scherrer analysis (Table S4) gives coherent domain sizes of ≈23.8 nm (textile blue), 18.9 nm (industrial coloured), and 25.3 nm (UiO-66-I), compared with smaller values of ≈13.5 nm for both food box and commercial UiO-66-NO_2_. These results show that recycled PET-derived MOFs can achieve crystallite sizes comparable to or larger than the commercial reference.

The TGA curves of UiO-type MOFs synthesised from different wPET sources show the characteristic three-step weight loss profile typical for this family of frameworks^81^ (Fig. 11C). An initial weight loss of ∼3–5% below 200 °C is assigned to the release of water and residual DMF molecules. This is followed by a gradual loss up to ∼330 °C corresponding to residual solvent removal and partial dehydroxylation of the Zr_6_ nodes. The main decomposition occurs between 330 and 500 °C with a mass loss of ∼65%, which is attributed to the combustion of the organic linkers (TA-NO_2_ or TA-I) and collapse of the framework.^81,82^ The final plateau above 500 °C corresponds to the formation of crystalline ZrO_2_, with residues of ∼32% for UiO-66-NO_2_ and ∼27% for UiO-66-I, in good agreement with theoretical values calculated for the respective ligands. The close overlap of decomposition temperatures and residues across PET-derived UiO-66-NO_2_ samples confirms their comparable purity and thermal stability, regardless of the wPET source.

Nitrogen sorption isotherms measured at 77 K are shown in Fig. 11D. All five samples exhibit the characteristic type-I profile of UiO-66 materials, with a sharp uptake at low relative pressure (*p*/*p*_0_ < 0.05) reflecting microporosity, accompanied by a weak type-IV tail and a small hysteresis loop near *p*/*p*_0_ ≈ 0.9–1.0, indicative of limited mesoporosity arising from interparticle condensation or framework defects. This confirms that all PET-derived samples retain the expected microporous character of UiO-66.

The UiO-66-I sample exhibits a surface area of 720 m^2^ g^−1^, in reasonable agreement with reported values (580–819 m^2^ g^−1^),^38,66^ and shows a mixed type-I/IV profile due to the presence of both micropores and minor mesoporosity. The commercial UiO-66-NO_2_ shows the steepest initial uptake and the highest overall capacity at *p*/*p*_0_ → 1, consistent with the largest BET surface area (Table S5) and micropore volume. In contrast, the food box, industrial, and textile blue PET-derived UiO-66-NO_2_ batches display a slightly reduced uptake at *p*/*p*_0_ < 0.05, corresponding to BET surface areas that were 10–20% lower. The reduced BET surface area is not attributable to loss of crystallinity, as confirmed by XRD analysis, but more likely arises from (i) a higher density of framework defects such as missing linkers or missing clusters, which reduce micropore volume while introducing small mesopores, (ii) residual modulators or occluded guests derived from PET, which can cap defects or block pores. NMR spectra of the MOF digestion product demonstrated the same TA/TA-R ratio as previously detected after isolation and also the presence of residual solvent in Fig. S17–S26. XRD-proved crystallinity with a similar particle size, similar TGA residual temperatures together evidence of slight high-pressure upturns and subtle hysteresis loops further support the presence of defect-related mesoporosity and/or interparticle voids.

The combined FTIR, XRD, TGA, and BET analyses demonstrate that UiO-66-I and UiO-66-NO_2_ prepared from upcycled PET possess the same structural integrity, thermal stability, and microporosity as their commercial counterparts. Although PET-derived UiO-66-NO_2_ shows slightly lower BET surface areas, this is linked to defect chemistry or occluded guests rather than loss of crystallinity. These findings confirm that wPET can serve as a viable alternative source of linkers for producing high quality UiO-type MOFs, enabling sustainable synthesis without compromising material performance.

### 2.7. Added-value of UiO-66-X derived from wPET

A central idea of functional upcycling is that polymer waste can be chemically transformed into materials whose performance and economic value exceed that of both the original polymer and analogous virgin-feedstock materials.^8^ This “added-value” concept, formalised in recent analyses of functional upcycling pathways, emphasises not only chemical transformation but value amplification per unit of waste plastic, a principle particularly relevant for PET-derived terephthalates.

Chemical upcycling (Fig. 1B) directly applies this concept by converting PET into TA-X, which are subsequently transformed into high-value UiO-66-X frameworks. Here, we demonstrated reagent-cost analysis (Tables S6 and S7), which shows that PET-derived TA-NO_2_ and TA-I are ∼5–7× and ∼75–80× cheaper, respectively, than the corresponding commercial linkers. As a result, the reagent cost of UiO-66-NO_2_ decreases from 2.2 to 1.6 € per g, while UiO-66-I drops drastically from 68.9 to 2.2 € per g when TA-I is sourced from PET (Tables S8 and S9).

Based on our previous report,^17^ UiO-66-I provides the strongest economic value for chlorinated contaminants removal. UiO-66 is of low-cost but exhibits limited affinity toward chlorinated aromatics (*Q*_e_ ≈ 0.7–1.1 g g^−1^), resulting in treatment costs of 8000–12 000 € per 100 m^3^ of 150 µM dichlorobenzene (DCB). UiO-66-I offers excellent capacities (*Q*_e_ = 2.4–2.9 g g^−1^) but is economically untenable because commercial TA-I (>59 € per g) drives purification costs to 66 000–164 000 € for the same scenario. However, PET-derived TA-I (∼0.78 € per g) reduces the UiO-66-I synthesis cost to 1.0–1.3 € per g, while the hybrid material containing UiO-66-I-50 retains high performance (*Q*_e_ = 1.88 g g^−1^). The resulting purification cost is ≈2531 € per 100 m^3^ DCB and it is 3–5× lower than UiO-66, making the wPET derived material an economically viable iodinated UiO-66 variant for large-scale treatment of chlorinated pollutants.

The UiO-66-NO_2_ case demonstrates that functionalisation *via* PET-derived linkers also amplifies catalytic value.^28^ UiO-66-NO_2_ increases the citronellal cyclisation rate constant from 0.012 to 0.673 h^−1^ (a 56-fold enhancement) and lowers the activation barrier by 19 kJ mol^−1^. However, commercial TA-NO_2_ (∼0.70 € per g) renders UiO-66-NO_2_ 2–3× more expensive than UiO-66, limiting its practical relevance. wPET-derived TA-NO_2_ (∼0.13 € per g) eliminates this barrier: it becomes nearly cost-equivalent to UiO-66 while offering ∼50× higher catalytic productivity per euro. This positions UiO-66-NO_2_ as a high performance, cost-competitive Lewis-acid catalyst representing a case where linker functionalisation improves both catalytic performance and economic accessibility.

Overall, the integration of PET-derived TA-X linkers generates two distinct and complementary added-value increase pathways: (i) performance-driven increase (higher *Q*_e_, higher catalytic rates) and (ii) cost-driven increase (linker price reduction of 7×–80×). Altogether, these effects enable UiO-66-X materials to be obtained *via* chemical upcycling, where waste PET is transformed into MOFs that outperform their virgin-feedstock analogues both technically and economically.

## 3. Conclusion

In this work, we have demonstrated a robust and broadly applicable method for the upcycling of post-consumer and industrial waste PET into value-added functionalised terephthalic acid derivatives. Through an electrophilic substitution strategy, we accessed TA-NO_2_ and TA-I under optimised conditions defined by the environmental energy impact factor (EEIF), achieving a favourable balance between yield, resource efficiency, and sustainability. The method proved universal across diverse PET feedstocks, including coloured and blended materials, affording high purity products with reproducibility. Furthermore, applying complementary sustainability metrics or full life-cycle assessment may offer an even more comprehensive evaluation of the broader environmental benefits of these upcycling routes.

Mechanistic studies demonstrate that PET hydrolysis precedes nitration under the investigated conditions, while partial electrophilic functionalisation also occurs directly on the polymer surface. This finding provides significant insight into electrophilic functionalisation of aromatic polyesters and broadens the scope of polymer upcycling beyond conventional depolymerisation-first strategies.

Importantly, the prepared linkers were successfully employed in the synthesis of UiO-type MOFs, which retained crystallinity, porosity, and thermal stability comparable to frameworks prepared from commercial linkers. Beyond NO_2_ and I, this strategy is compatible with a wide range of electrophilic substitutions commonly used in MOF linker design, including halogenation (–Cl and –Br), sulfonation (–SO_3_H), Friedel–Crafts acylation (–CO–R), and further nitration patterns. This enables the generation of PET-derived terephthalates bearing functional groups routinely employed to tune linker polarity, acidity, and adsorption behaviour. Overall, our findings establish upcycling as a viable platform for converting plastic waste into functional reagents, expanding the chemical space available for MOF synthesis, and contributing to the advancement of circular and sustainable chemical technologies.

## Conflicts of interest

There are no conflicts to declare.

## Supplementary Material

MH-013-D5MH02440D-s001

## Data Availability

The data supporting this article have been included as part of the supplementary information (SI), which contains detailed experimental procedures, kinetic and mechanistic studies, comprehensive characterisation (NMR, HPLC, FTIR, UV-Vis) and environmental/cost analyses for the upcycling of waste PET into functionalised linkers and UiO-66-X metal–organic frameworks. See DOI: https://doi.org/10.1039/d5mh02440d.
