# Supplementary material for: Electrophilic substitution in poly(ethylene terephthalate) waste for upcycling to functional metal–organic frameworks
Source: Mater Horiz. 2026 Sep 2. Online ahead of print. doi: 10.1039/d5mh02440d (PMC13637027; doi:10.1039/d5mh02440d)
Supplement: Supplementary file 1 [file MH-OLF-D5MH02440D-s001.pdf]

Supplementary Information (SI) for Materials Horizons.
This journal is © The Royal Society of Chemistry 2026

Electronic supplementary material

for

Electrophilic Substitution in Poly(ethylene terephthalate) Waste for Upcycling to Functional Metal–Organic Frameworks

Daniil Bryankin[1,2], Aleksey Ivanov[1], Natalia Soldatova[1], Mekhman Yusubov[1], Shaghayegh Naghdi[3], Dominik Eder[3], Markus Valtiner[2], Haritz Sardon[4], Pavel Postnikov[1,5*], Olga Guselnikova[1,2*]

[1] Research School of Chemistry and Applied Biomedical Sciences, Tomsk Polytechnic University, Lenin Avenue 30, Tomsk 634050, Russian Federation.

[2] Institute of Applied Physics, Vienna University of Technology, 1040 Vienna, Austria

[3] Institute of Materials Chemistry, Vienna University of Technology, Vienna 1060, Austria

[4] POLYMAT, University of the Basque Country UPV/EHU, Joxe Mari Korta Center, Avda. Tolosa 72, 20018 Donostia-San Sebastian, Spain

[5] N.N. Vorozhtsov Institute of Organic Chemistry, Pr. Ak. Lavrentieva 9, Novosibirsk 630090, Russian Federation

Table of Contents

Section 1. Experimental section 2

1.1 PET samples, chemicals and reagents 2

1.2 Experimental procedure for PET upcycling 3

1.2.1 wPET pretreatment 3

1.2.2 Upcycling to TA-NO_2 4

1.2.3 Upcycling to TA-I 5

1.2.4 Determination of predominant nitration mechanism 6

1.3 Green chemistry metrics 7

1.4 Metal-organic framework preparation 7

1.5 Characterization 7

1.6 Added value calculations based on costs estimation 9

Section 2. Feedstock PET wastes and processing 10

Section 3. Optimization of wPET upcycling to TA-NO_2 and TA-I 12

3.1 Optimization of wPET upcycling to TA-NO_2 12

3.2 Kinetic study of the correlation between initial reaction rate and intrinsic viscosity of wPET 14

3.3 Upcycling of different wPET into TA-NO_2 15

3.4 Optimization of wPET upcycling to TA-I 19

Section 4. EEIF Calculation for Conventional Preparation of 2-Iodoterephthalic Acid 21

Section 5. PET nitration mechanism 24

Section 6. Characterization of synthesized UiO-66-X 26

Section 7. Estimated reagent cost of PET-Derived TA-X linkers and UiO-66-X MOFs 32

7.1 Catalytic productivity per unit cost 33

References 35

Section 1. Experimental section

1.1 PET samples, chemicals and reagents

Transparent polyethylene terephthalate (PET) foodboxes and bottles were collected after consumption of food in our laboratory. The samples of industrial plastic wastes (~5-7 mm size) were provided by the company GAIKER (Basque Country) in two batches – colourless (PET) and coloured (PET/ polyethylene (PE) blend). Samples of the red and the blue polyesters were collected from Tomsk textile workshop waste (Figure S1, S2).

Dimethyl sulphoxide-d$_6$ (DMSO-d$_6$, anhydrous, 99.8 %) was purchased from ROTH (Bavaria, Germany), deuterium oxide (D_2O, 99.9 atom % D), zirconium tetrachloride ($ZrCl_4$, anhydrous, 98.0 %), terephthalic acid (TA, 98 %) 2-nitroterephthalic acid (TA-NO_2, 99.0 %), 2-iodoterephthalic acid (TA-I, 98.0 %), bis(2-hydroxyethyl) terephthalate (BHET, 99.0 %) N,N-Dimethylformamide (DMF, anhydrous, 99.8 %), ethanol (95.0 %), ethyl acetate (99.5 %), sulfuric acid (H_2SO_4, 98.0 %), nitric acid (HNO_3, 65.0 %), hydrochloric acid (HCl, 36.5 %), iodine (I_2, 99.5 %), sodium sulfite (Na_2SO_3, 99 %), sodium hydroxide (NaOH, 99 %) sodium nitrate ($NaNO_3$, 99 %) and sodium chloride (NaCl, 99.5 %) were purchased from Sigma-Aldrich and used without further purification.

1.2 Experimental procedure for PET upcycling

1.2.1 wPET pretreatment

Firstly, industrials (coloured, colourless) samples, food boxes and bottles were carefully washed by water using common domestic detergent and dried on air. The dried materials were crushed by domestic coffee grinder until formation of powder with an average size of around 0.5–1.5 mm. For the standardization, the resulting powders were additionally classified using laboratory sieves and the fraction less than 1 mm was used for further experiments (Figure S2). Textile wPET were cut into pieces with an average size of around 1.5± 0.7 cm and used without any other treatment (Figure S1).

For industrial plastic waste (coloured PET/PE blend) PE content was determined according to following procedure: Sodium hydroxide 4 g (100 mmol) were dissolved in 150 mL of distilled water, and 2 g (10.4 mmol) of industrial coloured blend wPET/PE were added to the resulting

solution and stirred at 100 °C for 48 h. Next, the reaction mixture was cooled to room temperature, the remaining pieces of PE were filtered off, washed with 100 mL of distilled H_2O, dried in vacuo for 24 h, and weighed for gravimetric determination of the PE content (Equation S1).

$$\text{PE, \%} = \frac{\text{mass PE}}{\text{mass industrial wPET/PE}} \times 100 \qquad \text{(S1)}$$

1.2.2 Upcycling to TA-NO_2

A typical procedure for upcycling PET to TA-NO_2 involves the following steps. Crushed PET (2 g, 10.4 mmol, particle size < 1 mm) was portion wise added to a preheated mixture of HNO_3 and H_2SO_4 (loadings and temperatures are provided in Table 1, Main text) under vigorous stirring. The reaction mixture was stirred until full consumption of PET. The reaction mixture was diluted by ice-cooled water followed by portion wise addition of solid NaOH to adjust the pH to 11–12. The resulting mixtures was filtered and solid NaCl was added to the filtrate. The resulting solution was acidified by concentrated HCl to pH 0–1 under cooling. The precipitated solid was collected by filtration. The aqueous phase was extracted three times with equal volumes of ethyl acetate. The combined organic extracts were dried, and the solvent was evaporated under reduced pressure. The combined solid product was further dried under high vacuum.

Under optimal conditions, 2 g (10.4 mmol) of crushed PET (particle size < 1 mm) was added to a mixture of 7.2 mL HNO_3/H_2SO_4, preheated to 100 °C, in a volume ratio of 1.8 mL (26.00 mmol) to 5.4 mL (99.36 mmol), respectively. The reaction mixture was stirred 2 h and diluted by ice-cooled water (80 mL) followed by addition of solid NaOH (approx. 9 g) to adjust the pH to 11–12. The resulting mixtures was filtered and solid NaCl (approx. 4 g) was added to the filtrate. The resulting solution was acidified by concentrated HCl (approx. 4 mL) to pH 0–1 under cooling. The work up of mixture was performed according to aforementioned procedure (Figure S1A).

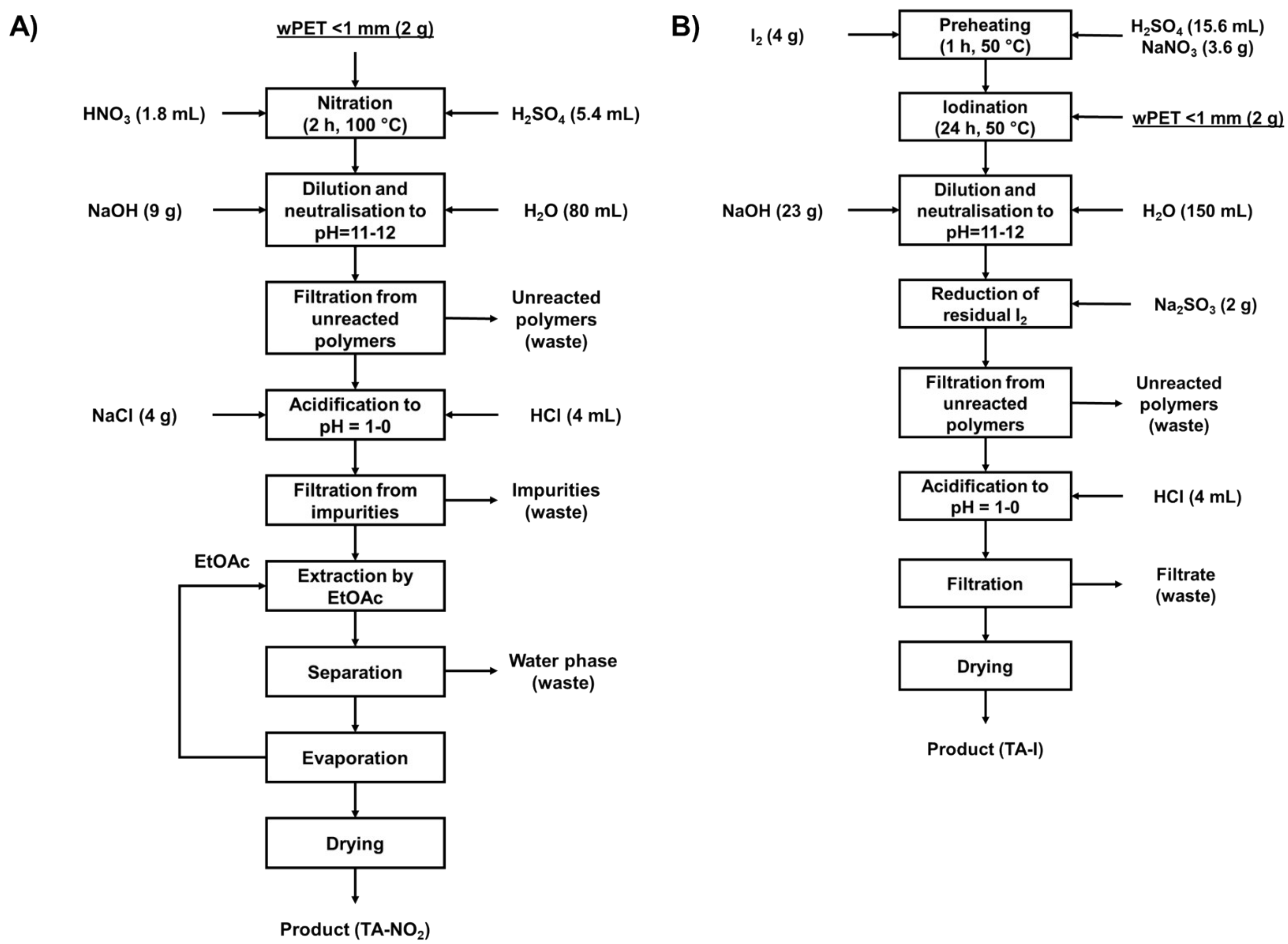

Figure S1 –Scheme of upcycling processes for $TA\text{-}NO_2$ (A) and TA-I (B) production

1.2.3 Upcycling to TA-I

A typical procedure for the upcycling of PET to TA-I involved the following steps. Crushed I_2 4.00 g (15.75 mmol) was added to a preheated mixture of $NaNO_3$ (or HNO_3) (quantities and temperatures are provided in Table 2, Main text) and H_2SO_4 15.60 mL, (287 mmol), under vigorous stirring. After 1 hour, 2.00 g (10.4 mmol) of crushed coloured industrial wPET (particle size < 1 mm) was added to the resulting mixture. The resulting mixture was heated during 24 h. The reaction mixture was diluted by ice-cooled water (150 mL) followed by addition of solid NaOH to adjust the pH to 11–12. Solid Na_2SO_3 was then added to ensure complete reduction of residual

I_2. The filtrate was subsequently acidified to pH 0–1. The precipitated solid was collected by filtration and dried under high vacuum.

Under optimal conditions, finely crushed I_2 powder 4.00 g (15.75 mmol) was added to a preheated to 50 °C mixture of 3.58 g (42.13 mmol) $NaNO_3$ and H_2SO_4 15.60 mL (287 mmol), under vigorous stirring. After 1 hour, 2.00 g (10.4 mmol) of crushed coloured industrial wPET (particle size < 1 mm) was added to the resulting mixture. The resulting mixture was heated during 24 h. The reaction mixture was diluted by ice-cooled water (150 mL) followed by addition of solid NaOH (approx. 23 g) to adjust the pH to 11–12. Solid Na_2SO_3 (approx. 2 g) was then added to ensure complete reduction of residual I_2. The filtrate was subsequently acidified by concentrated HCl (approx. 6 mL) to pH 0–1. The precipitated solid was collected by filtration and dried under high vacuum (Figure S1B).

1.2.4 Determination of predominant nitration mechanism

A typical procedure for the **nitration of commercial TA:** 1.7264 g (10.4 mmol) of the TA powder was added to 7.2 mL of nitrating mixture, preheated to 100 °C (a volume ratio of HNO_3/H_2SO_4 1.8/5.4 mL/mL, respectively). The work up of reaction mixture was performed according to aforementioned procedure (Section S1.2.2). The product was analysed by NMR spectroscopy.

A typical procedure for the **partial nitration of wPET film:** The polymer film (15*15 mm) was exposed to the nitrating mixture (~0.7 mL) at the optimal conditions (100 °C, 2.5 equiv of HNO_3 with LSR = 3.6). The sample was removed from the reaction medium prior to complete dissolution, allowing it to retain its macroscopic structure and avoid extensive depolymerization. It was then washed with distilled water and ethanol, dried, and analysed by FTIR spectroscopy.

A typical procedure for the **nitration of BHET/TA equimolar mixture:** Powders of TA and BHET (0.216 g (2.6 mmol) and 0.330 g (2.6 mmol), respectively) were added to a preheated (100 °C) mixture of nitric and sulfuric acids (10 mL total; HNO_3/H_2SO_4 = 2.5/7.5 mL, v/v) under vigorous magnetic stirring. The mixture was stirred continuously until full reaction completed. The reaction mixture was analysed by HPLC chromatography according to following procedure.

A typical procedure for **kinetic experiment for determination of initial reaction rate**: Crushed PET sample (0.5 g, 2.6 mmol, particle size < 1 mm) was added to a mixture of nitric and

sulfuric acids (10 mL total; HNO_3/H_2SO_4 = 2.5/7.5 mL, v/v) under vigorous magnetic stirring. The reaction mixture was stirred continuously until complete dissolution of the polymeric substrate.

At predetermined time intervals, a 100 μL aliquot of the reaction mixture was withdrawn and immediately diluted with 12.5 mL of acetonitrile (MeCN). The resulting solution was then brought to a final volume of 50 mL by sequential addition of 0.1 % trifluoroacetic acid (TFA) in water and 0.1 M NaOH solution, adjusting the pH of the final sample to 2.5 ± 0.1 to ensure analyte stability and reproducibility of chromatographic detection.

1.3 Green chemistry metrics

For the estimation of possible environmental and technological impact, the environmental energy impact factor (EEIF, ξ) was implied (Equation S2).

$$\xi = \frac{m_{reagents} \cdot \int_0^T T(t)dt}{\varphi_{product} \cdot m_{product}} \quad \text{(S2)}$$

where $m_{reagents}$ - mass of reagents; T - temperature in °C; t - time in minutes; $m_{product}$ - mass of isolated TA-R; $\varphi_{product}$ - yield of isolated TA-R.

For multi-step processes (three-step synthesis of 2-iodoterephthalic acid starting from TA EEIF was calculated as sum of EEIF of all steps (Equation S3).

$$\xi = \xi_{Nitration} + \xi_{Reduction} + \xi_{Diazotization} + \xi_{Iodination} \quad \text{(S3)}$$

1.4 Metal-organic framework preparation

UiO-66-X were prepared according to the procedure described by Katz et al.[1] The metal precursor solution was prepared by dissolving $ZrCl_4$ 126 mg (0.54 mmol) in a mixture of DMF (5 mL) and concentrated HCl (1 mL), followed by sonication for 10 min. The solution of TA-R (0.75 mmol) in DMF (5 mL) was prepared in separate vessel. The solutions of $ZrCl_4$ and corresponding ligands were mixed and transferred to a Teflon-lined autoclave. The mounted autoclave was maintained at 80 °C for 24 h. After, the autoclave was cooled to room temperature and the solid product was isolated by centrifugation and washed with methanol. The resulting MOF crystals were activated at 80 °C for 12 h under vacuum.

For digestion of MOF UiO-66-R were dispersed in 1M $NaHCO_3$ solution in D_2O, after which ultrasonic treatment was performed for 20 min.[2]

1.5 Characterization

NMR spectroscopy

The nuclear magnetic resonance (NMR) spectra were recorded on a BRUKER AVANCE III HD spectrometer (400 MHz), using DMSO-*d6* as the solvent for TA-R powders and 1M $NaHCO_3$ solution in D_2O as digested agent and solvent for UiO-66-R powders. ^1H NMR spectra were recorded at 400 MHz. Chemical shifts are reported in parts per million (ppm). ^1H chemical shifts are referenced relative to the residual solvent signal.

TA-R content was calculated according to equation (S4) and used for further calculations:

$$\text{TA - R, \%} = \frac{\text{TA - R}_{ia}}{\text{TA - R}_{ia} + (\text{TA}_{ia}/4)} \times 100 \qquad \text{(S4)}$$

Where $TA\text{-}R_{ia}$ is the integral area under the TA-I peak (8.41 ppm) or $TA\text{-}NO_2$ peak (8.39 ppm), and TA_{ia} is the integral area under the TA peak (8.05 ppm), respectively.

High-performance liquid chromatography (HPLC)

HPLC mobile phases were prepared using a Milli-Q IQ 7003 (Merck) water purification system. HPLC-grade acetonitrile was obtained from Panreac and trifluoroacetic acid was obtained from Sigma-Aldrich and used without further purification. HPLC was performed on an Agilent 1200 HPLC system with a diode array detector (1260 DAD VL) using a Zorbax (Agilent Technologies, U.S.A.) Eclipse Plus C18 column (4.6 mm · 250 mm, 5 μm) at 30 °C. Analysis was performed using 0.1% TFA in H_2O (phase A)–CH_3CN (phase B) as the mobile phase in isocratic mode with the ratio A to B equal to 75:25 for 10 min at a flow rate of 1 mL/min. The injection volume was 20 μL. UV-detector was operated on 240 nm wavelength. Before analysis, every sample was filtered through a 0.45 μm PTFE syringe filter. The commercially available terephthalic, 2-nitroterephthalic, 2-iodoterephthalic acids and bis(2-hydroxyethyl) terephthalate were used as a reference.

Ultraviolet-visible spectroscopy

The absorption spectra were recorded in the ultraviolet-visible (UV-vis) range (190-900 nm) using a Thermo Scientific Evolution 201 spectrometer (Thermo Fisher, USA).

Fourier transform infrared spectroscopy

The Fourier transform infrared (FT-IR) spectra were recorded on an Equinox 55 spectrometer (Bruker, USA) equipped with an attenuated total reflectance (ATR) accessory on a diamond crystal. Powder's spectra were collected over 100 scans with a resolution of 4 cm^{-1}. All spectra were corrected using a straight baseline in the 4000–400 cm^{-1} spectral region.

The same measurement conditions used for polymer films of wPET before and after partial nitration.

Powder X-ray diffraction measurements

The powder X-ray diffraction (PXRD) measurements studies were performed using a Malvern PANalytical XPert MPD Pro instrument (Malvern PANalytical, United Kingdom) over a scan range of 5–120°. The diffractometer was operated in a Bragg-Brentano configuration using a CuKα tube.

Physisorption measurement

N_2 physisorption measurements were conducted using a Micromeritics 3Flex surface characterization analyser. Prior to both measurements, the samples were degassed under vacuum at 120 °C for 10 hours to eliminate residual adsorbates. N_2 adsorption was performed at 77 K,) under equilibrium conditions. In the N_2 physisorption, the total pore volume was estimated from the amount of nitrogen adsorbed at a relative pressure (P/P_0) of 0.95, assuming that contributions from external surface adsorption were negligible compared to internal pore adsorption.[3] The apparent surface area was determined using the Brunauer–Emmett–Teller (BET) method following the recommended protocol for microporous materials.[4] Pore size distributions were obtained from the adsorption branch using nonlocal density functional theory (NLDFT) assuming a cylindrical pore model. All data processing and calculations were performed using the Flex Version 6.01 software (Micromeritics Instruments).

Thermogravimetry

Thermogravimetric (TG) studies were performed using a Perkin Elmer TGA8000 (PerkinElmer, Inc., USA). Each sample was placed in an alumina crucible and heated from 30 to 700 °C at a heating rate of 10 °C/min in air (20 mL/min).

1.6 Added value calculations based on costs estimation

Cost-based added-value calculations were performed using main-reagents-only considerations (Table S6-S9). The costs of stoichiometric reagents directly consumed in the reaction were included; solvents, work up, labour, energy, and equipment overheads were excluded because they affect UiO-66, UiO-66-NO_2, and UiO-66-I equally and do not change relative cost trends. Unit prices were taken from Carl Roth and Sigma-Aldrich (AT/DE region, 2025). Reaction stoichiometries and isolated yields correspond to the experimentally optimised procedures. No recycling of acids, iodine, or aqueous wash solutions was assumed.

Section 2. Feedstock PET wastes and processing

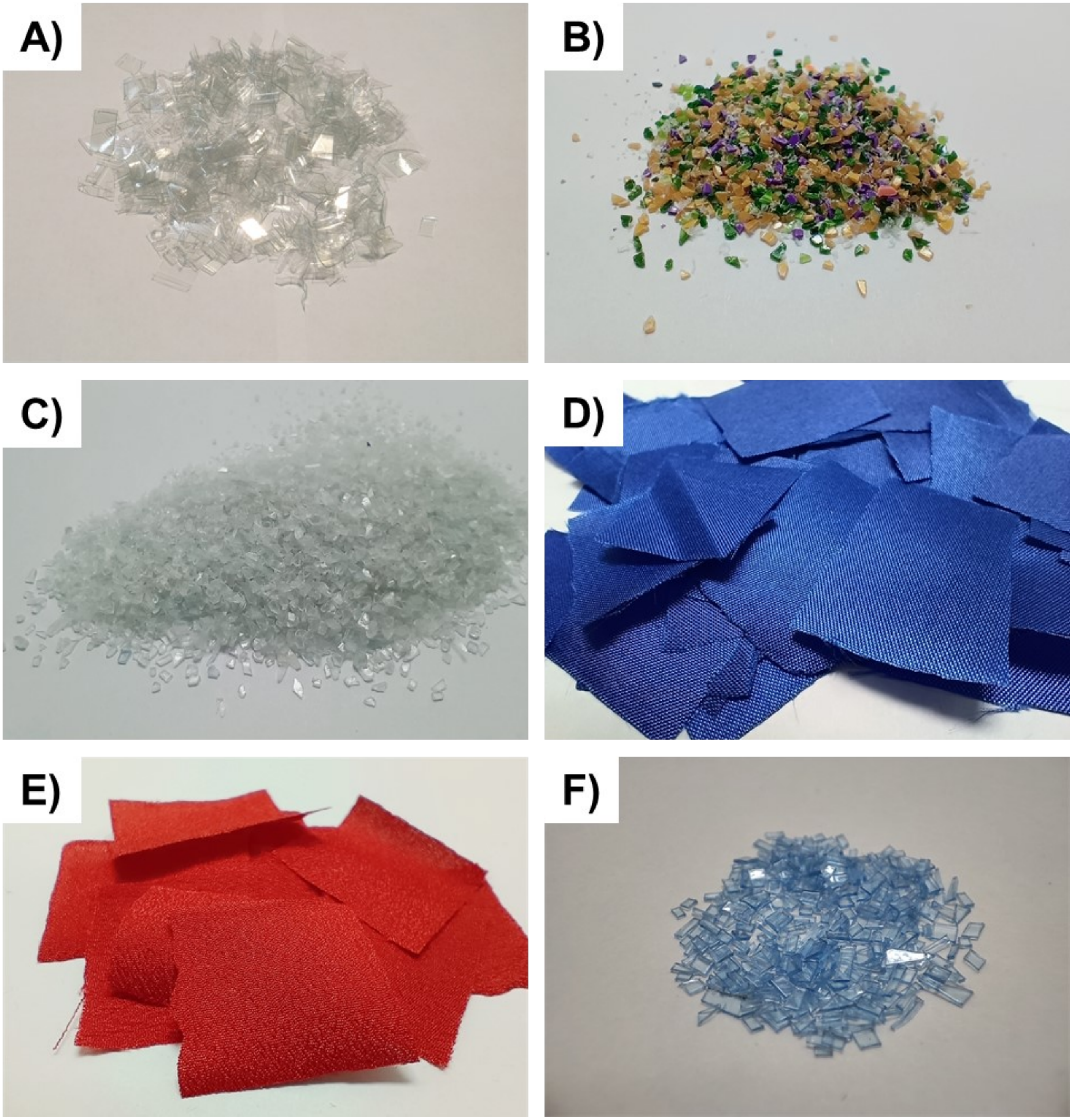

Figure S2 –Different PET wastes: A) post consumer food box; B) industrial colored (blend wPET/PE); C) industrial colorless; D) textile blue (polyester); E) textile red (polyester); F) bottle

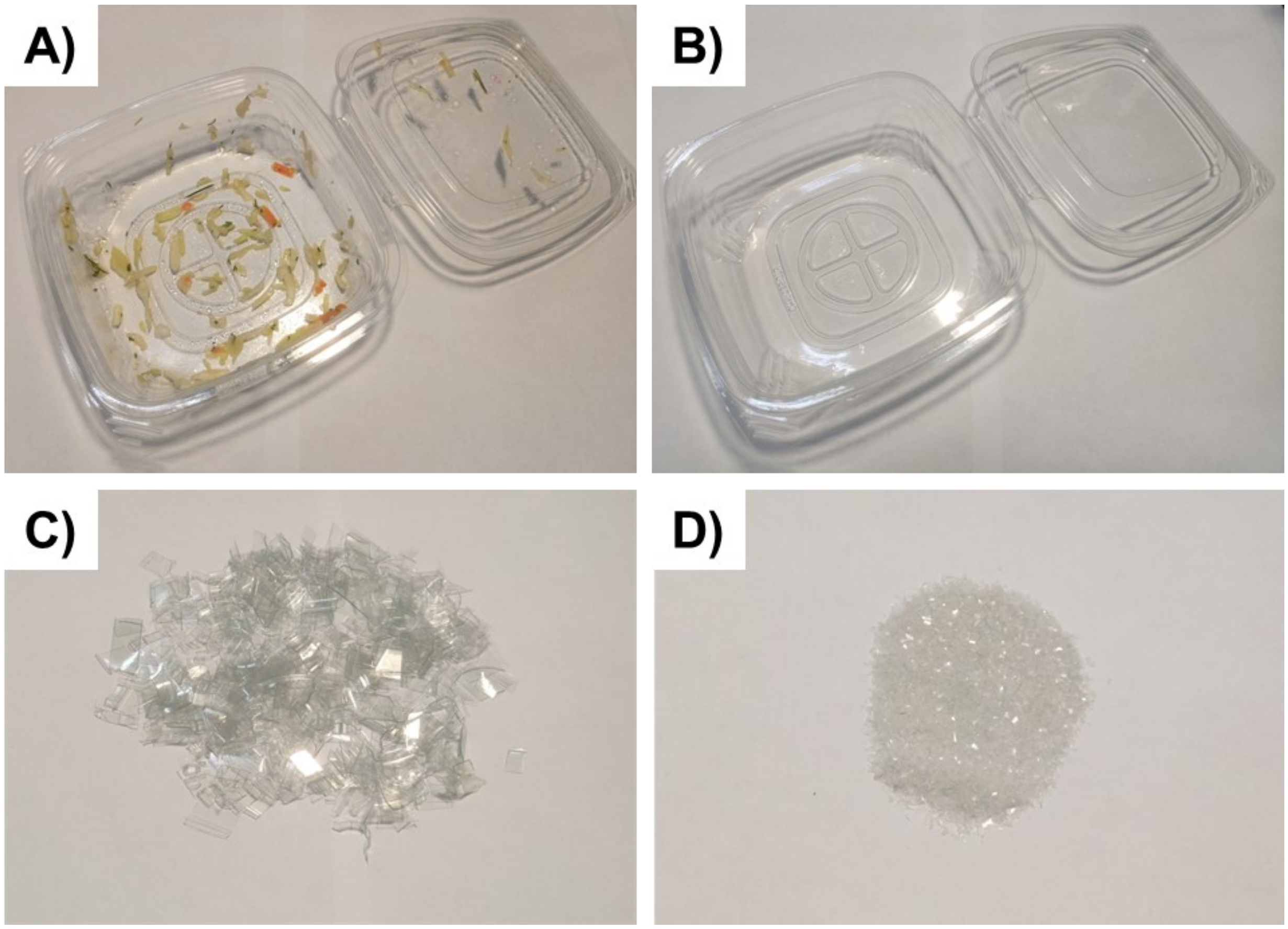

Figure S3 – PET waste preparation (using food container waste as an example) for the reaction: a) original one PET food box with food pieces; b) PET food box after washing and drying; c) PET after cut; d) crushed PET food boxes (particle size less than 1 mm)

Section 3. Optimization of wPET upcycling to TA-NO_2 and TA-I

3.1 Optimization of wPET upcycling to TA-NO_2

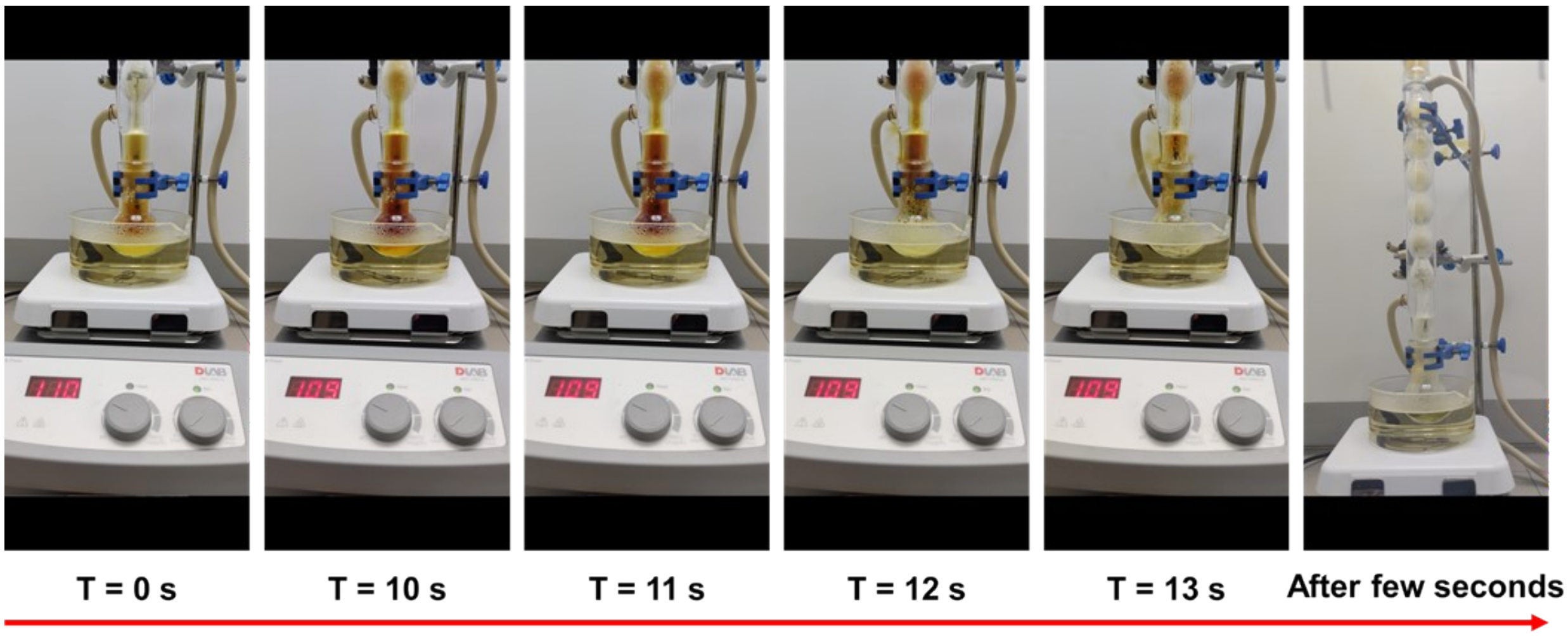

Figure S4 – Time-resolved frame sequence capturing the onset of rapid boiling of the reaction mixture, with significant carry-over into the condenser

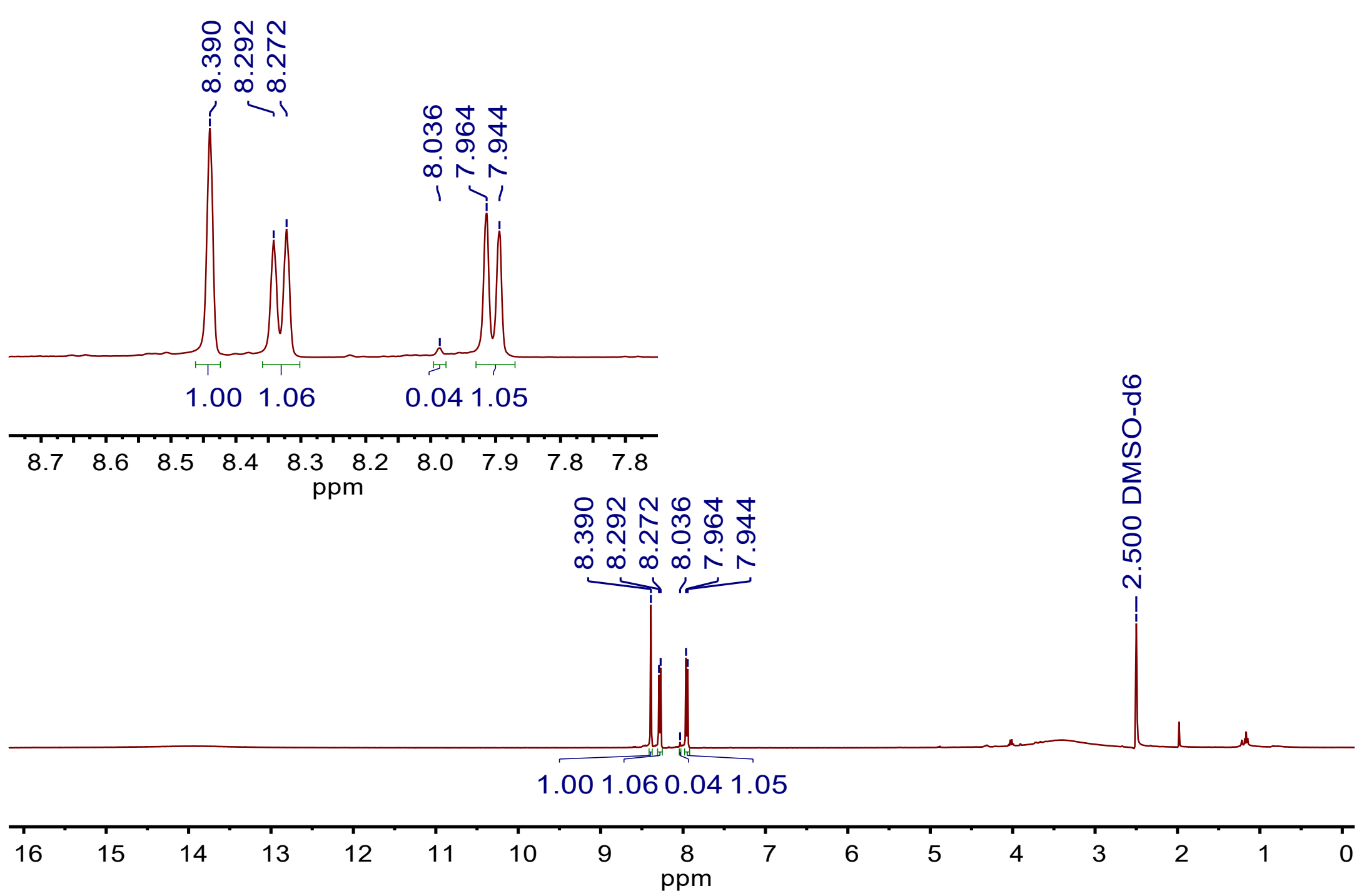

Figure S5 – ^1H NMR spectrum of TA-NO_2 isolated during the optimization procedure

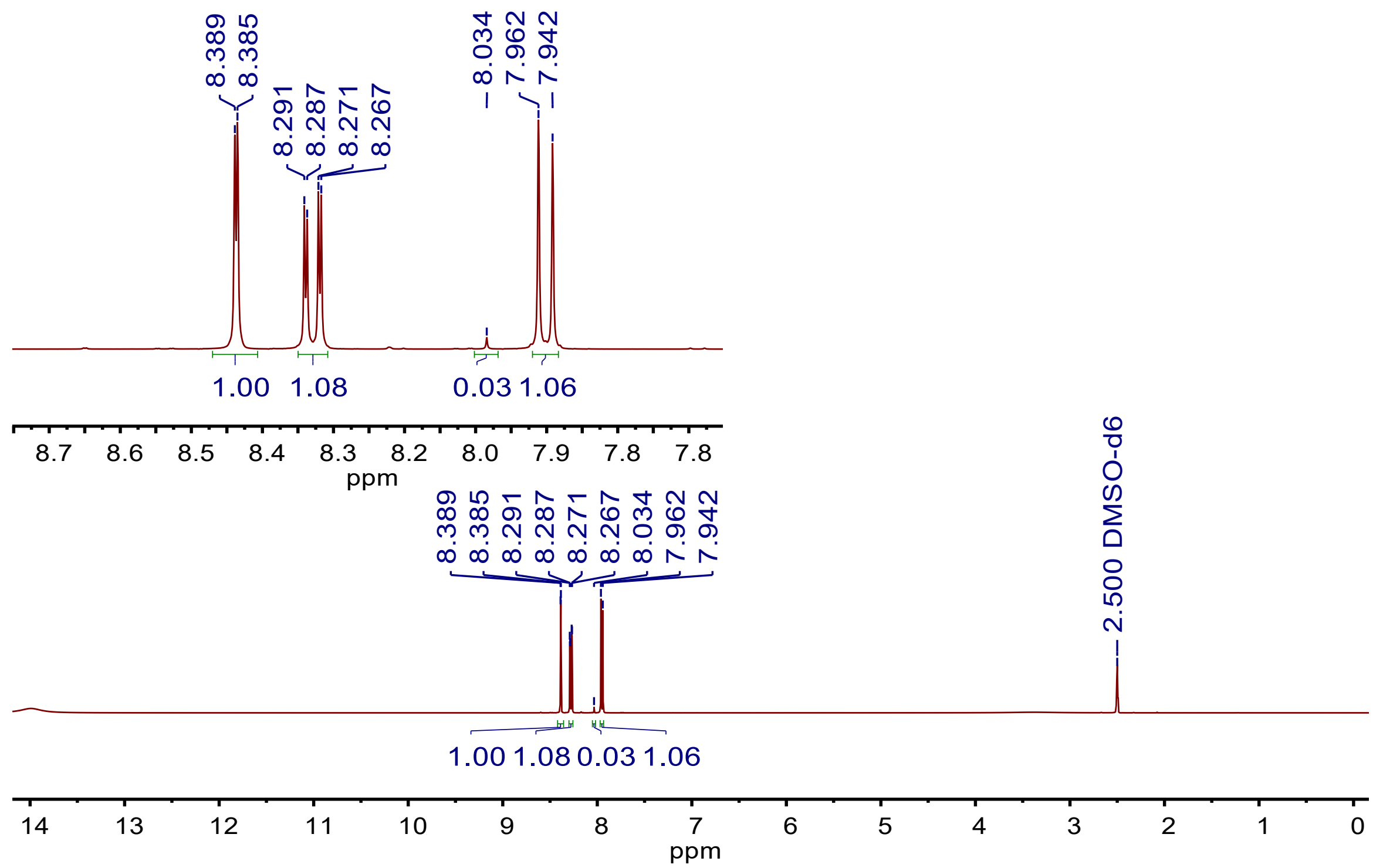

Figure S6 – ^{1}H NMR spectrum of commercially available TA-NO_2 [5]

3.2 Kinetic study of the correlation between initial reaction rate and intrinsic viscosity of wPET

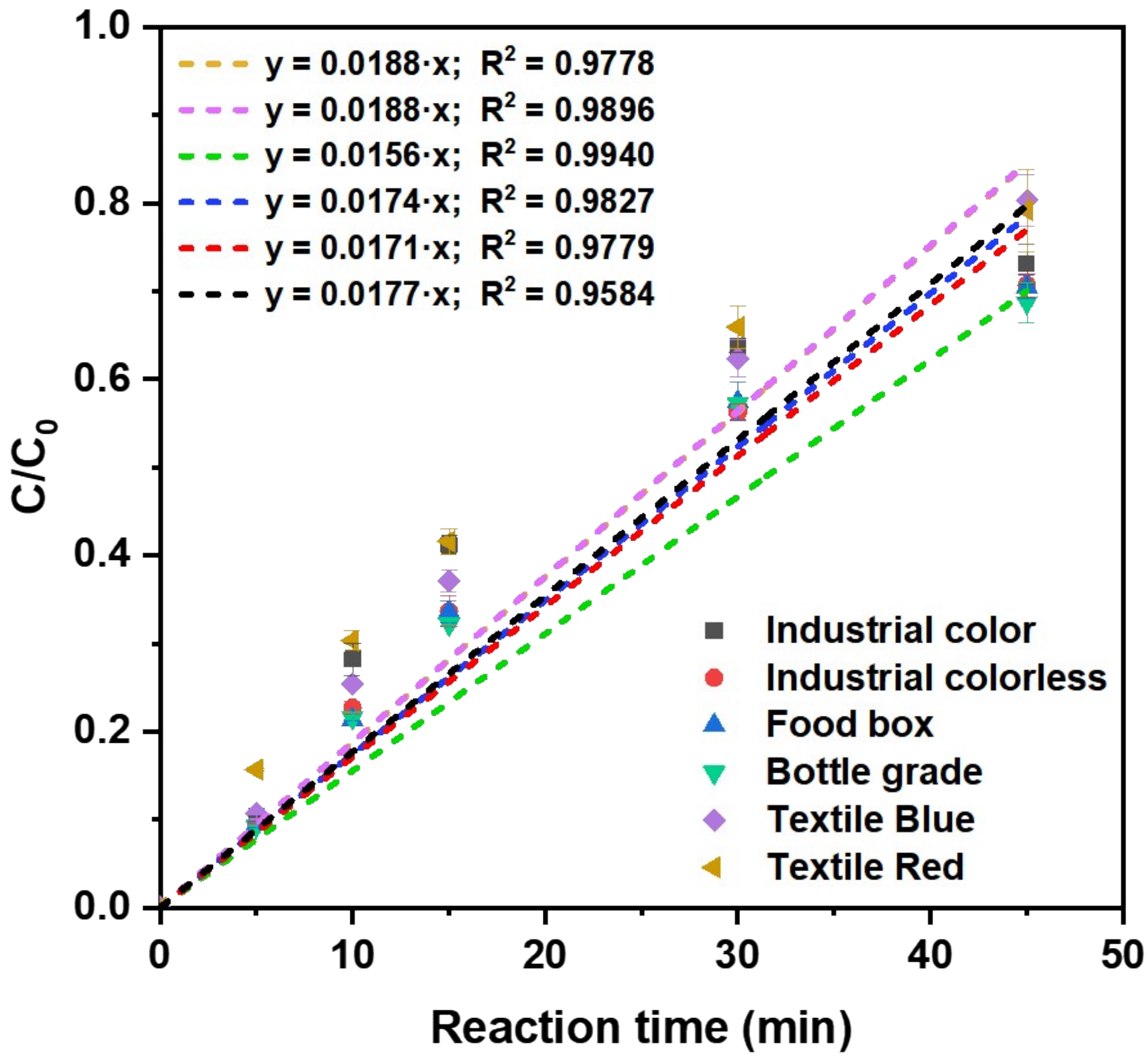

Figure S7 – Initial nitration rate calculation for different wPET sources

Dependencies of the relative peak area of the product (normalized to an external standard) versus reaction time were plotted (Figure S7). The initial linear region of the kinetic curve was identified and fitted by linear regression. The initial reaction rate was calculated as the slope of this linear segment, expressed in $mol·L^{-1}$ s^{-1} with account for the reaction volume and dilution factors. All experiments were performed in triplicate; uncertainty in rate determination is reported as standard deviation.

3.3 Upcycling of different wPET into TA-NO_2

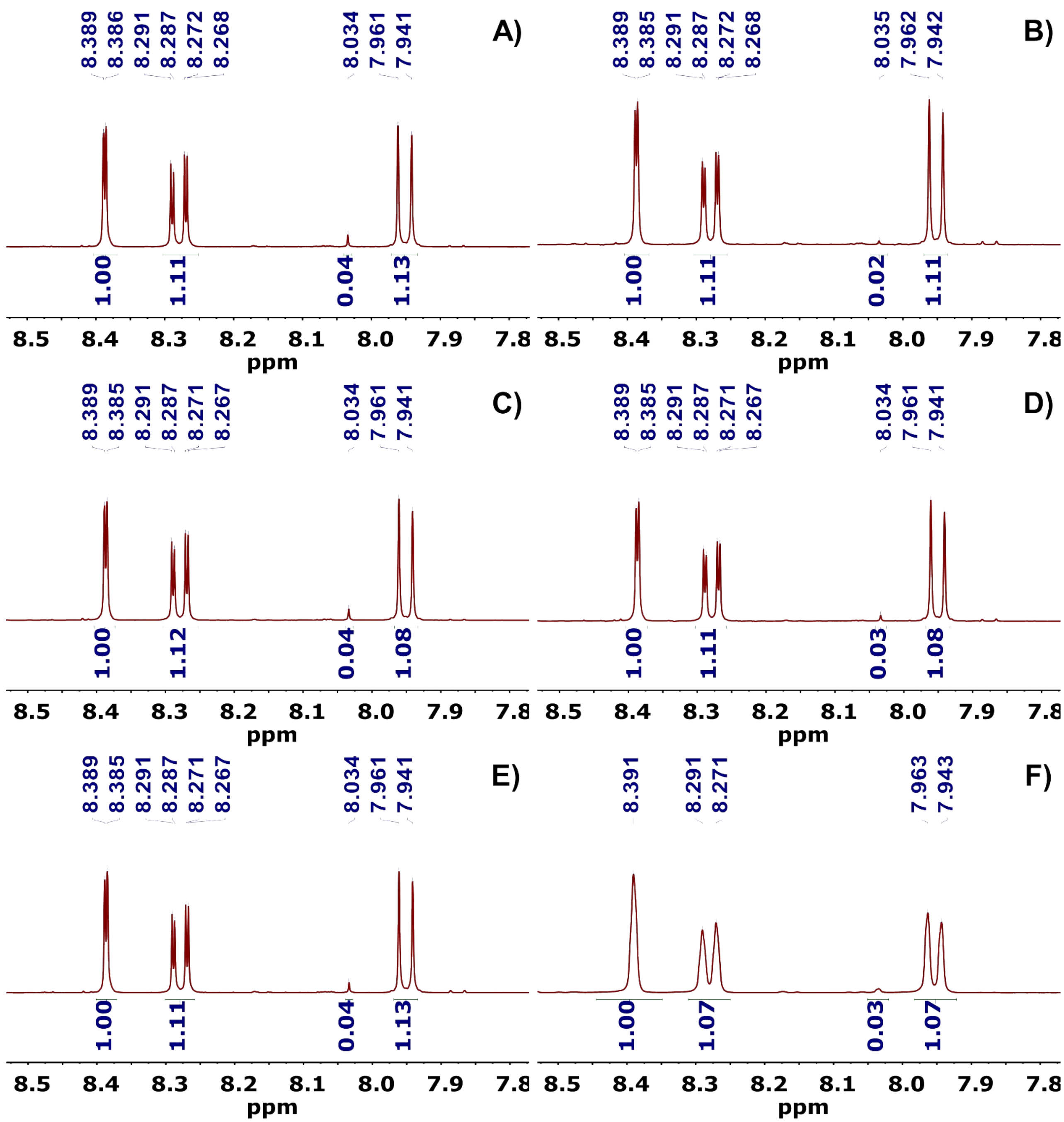

Figure S8 – Aromatic region of ^1H NMR spectra of isolated upcycled TA-NO_2 from different wPET sources: Food box (A), Industrial colored (B), Industrial colorless (C), Textile blue (D), Textile red (E), Bottle (F)

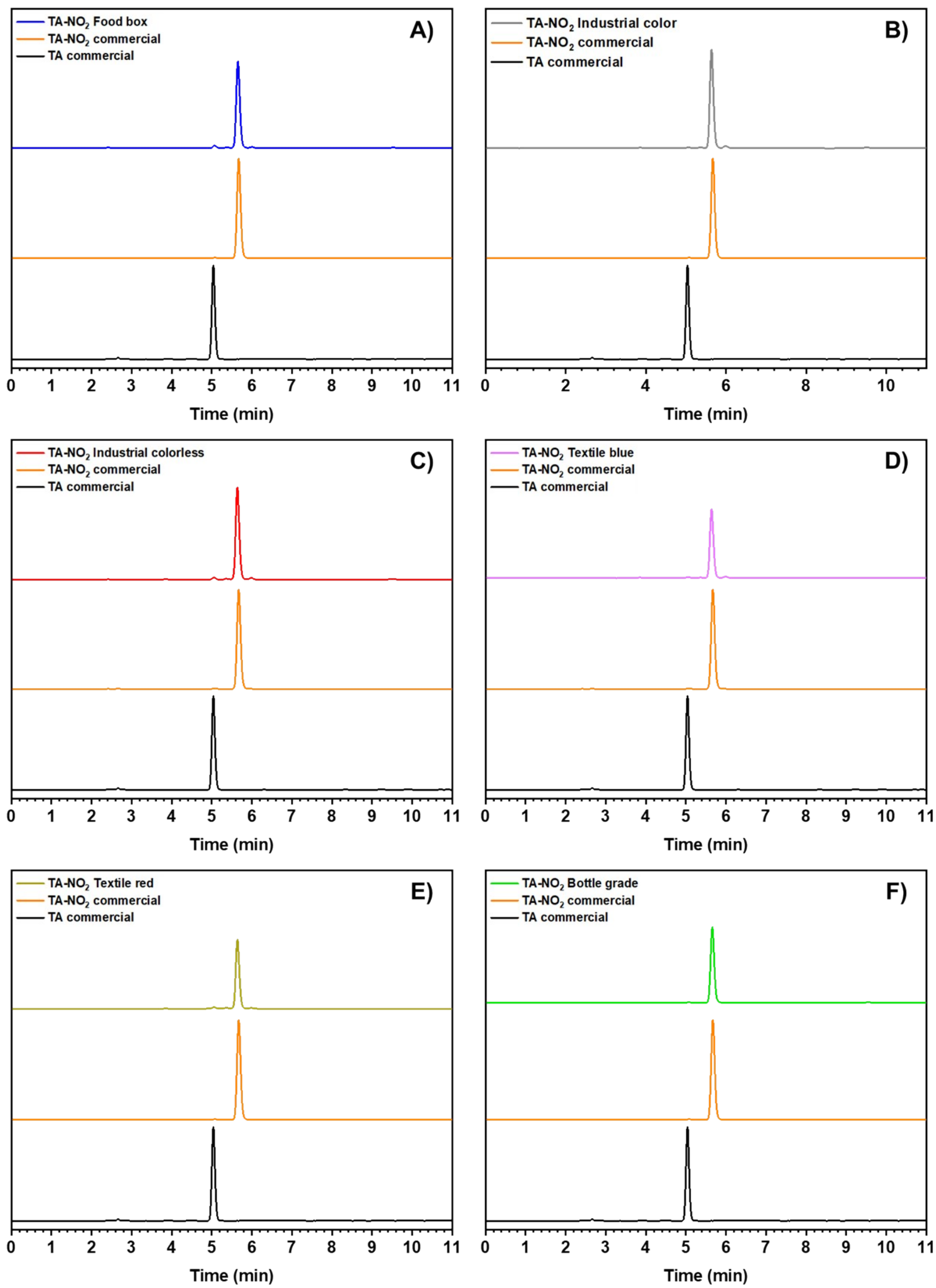

Figure S9 – Chromatograms of isolated TA-NO_2 after upcycling of Food box (A), Industrial coloured (B), Industrial colourless (C), Textile blue (D), Textile red (E), Bottle (F) waste PET and commercial TA and TA-NO_2

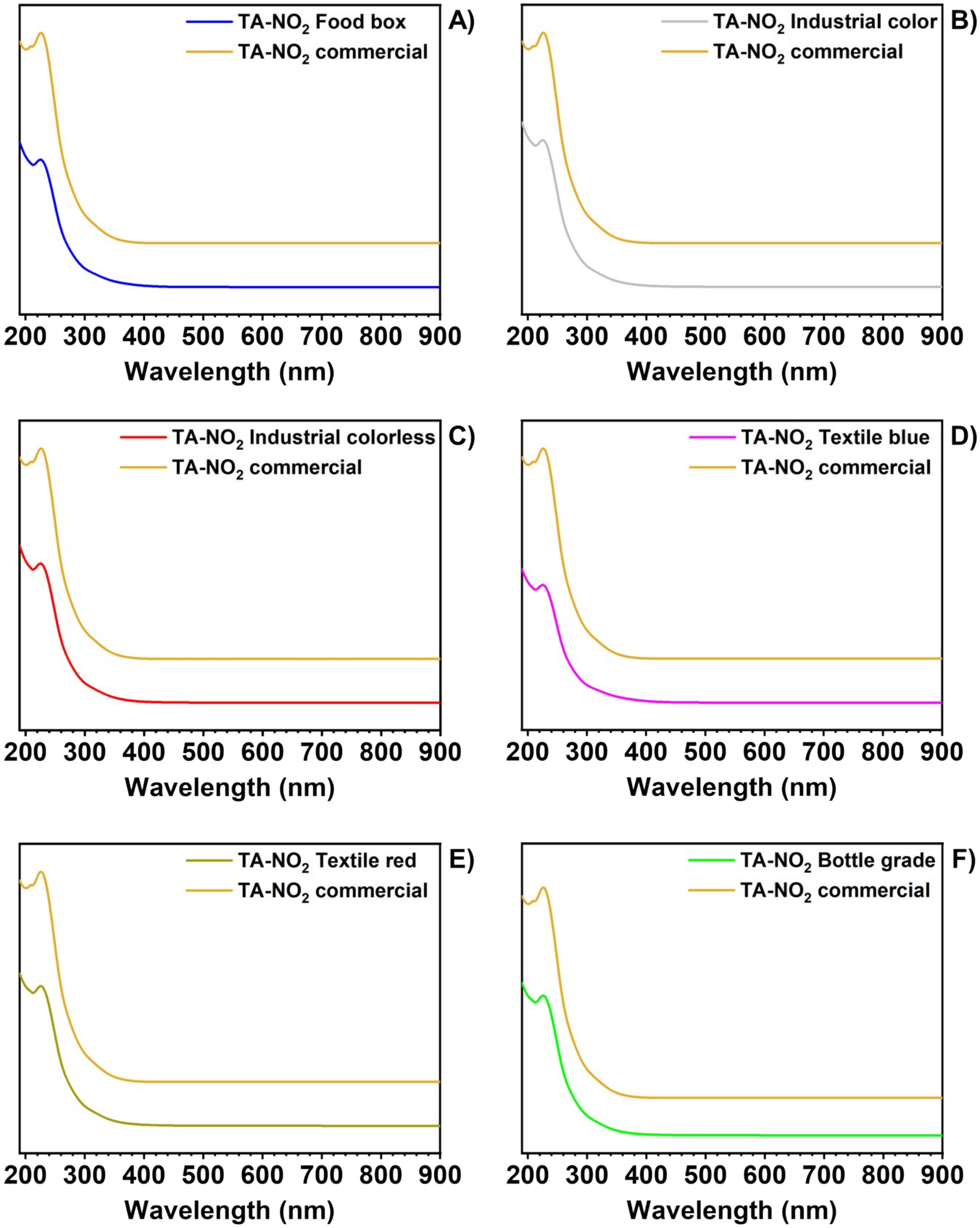

Figure S10 – UV-Vis spectra of isolated upcycled TA-NO_2 from different wPET sources: Food box (A), Industrial coloured (B), Industrial colourless (C), Textile blue (D), Textile red (E), Bottle (F) in comparison with commercial TA-NO_2

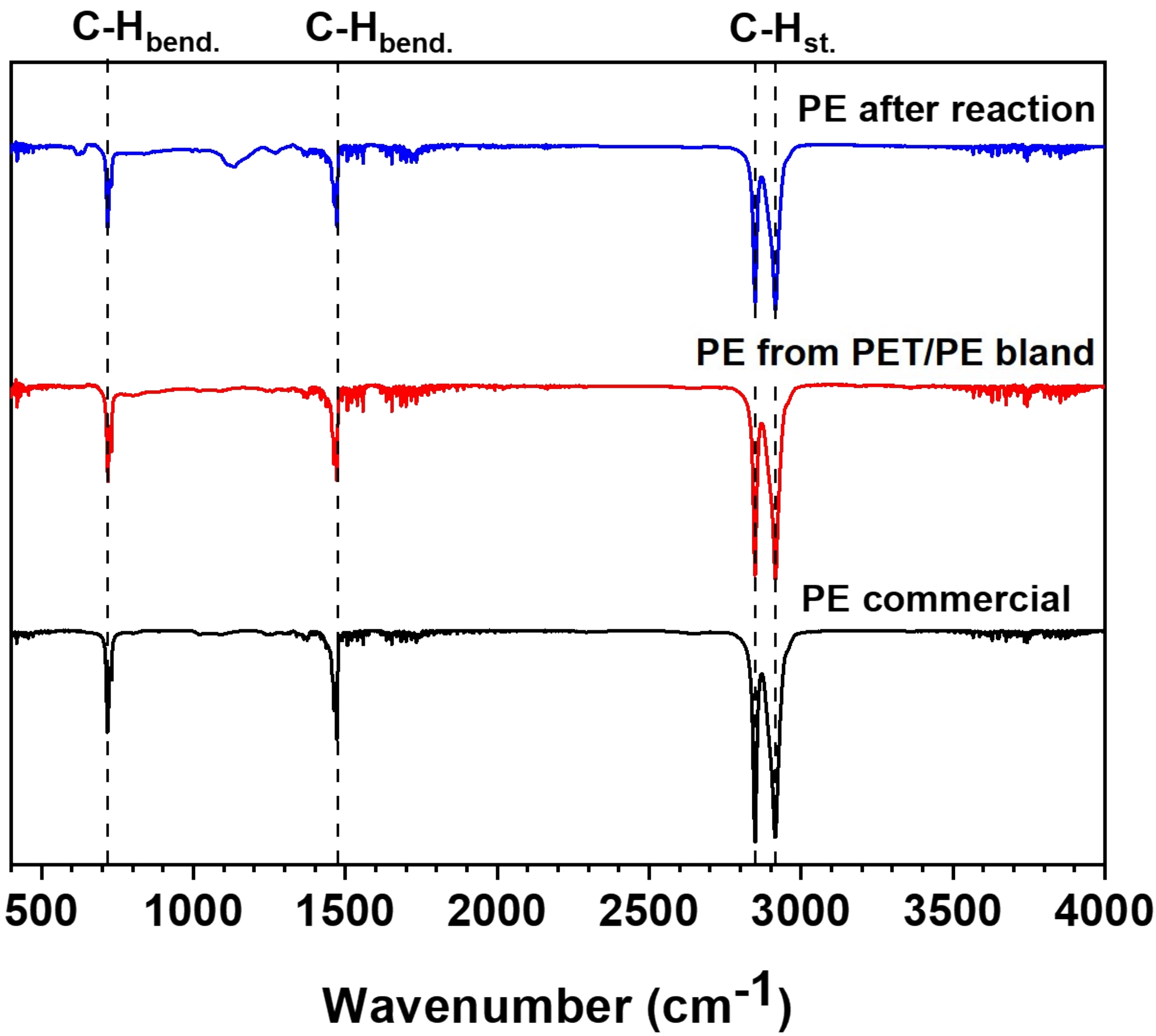

Figure S11 – FTIR-spectra of PE isolated after upcycling of coloured industrial wPET with PE impurities, PE before upcycling (mechanical separated) and commercial PE

3.4 Optimization of wPET upcycling to TA-I

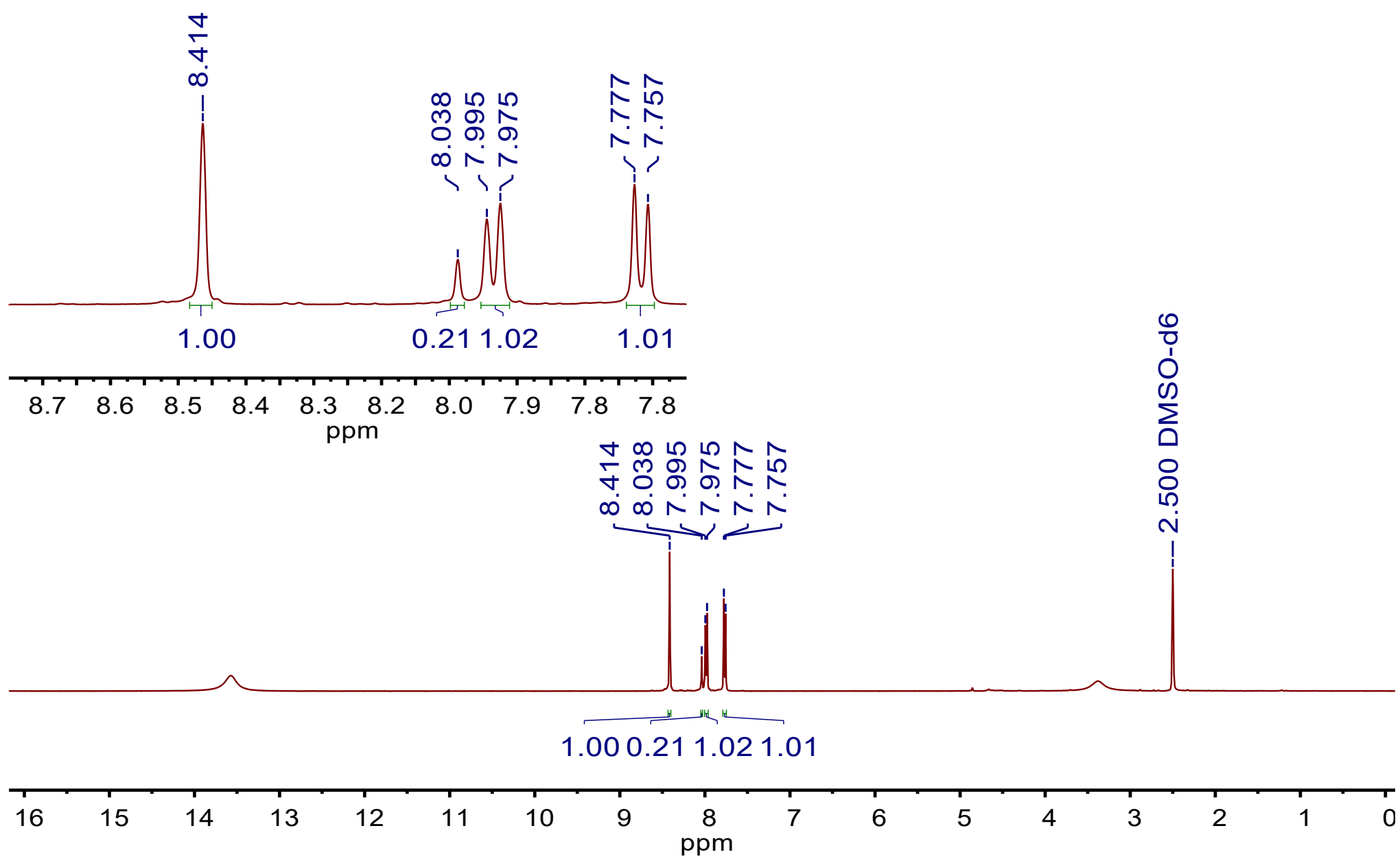

Figure S12 – ^{1}H NMR spectra of TA-I isolated at best conditions (50 °C, 24 h, 4 equiv of $NaNO_3$)

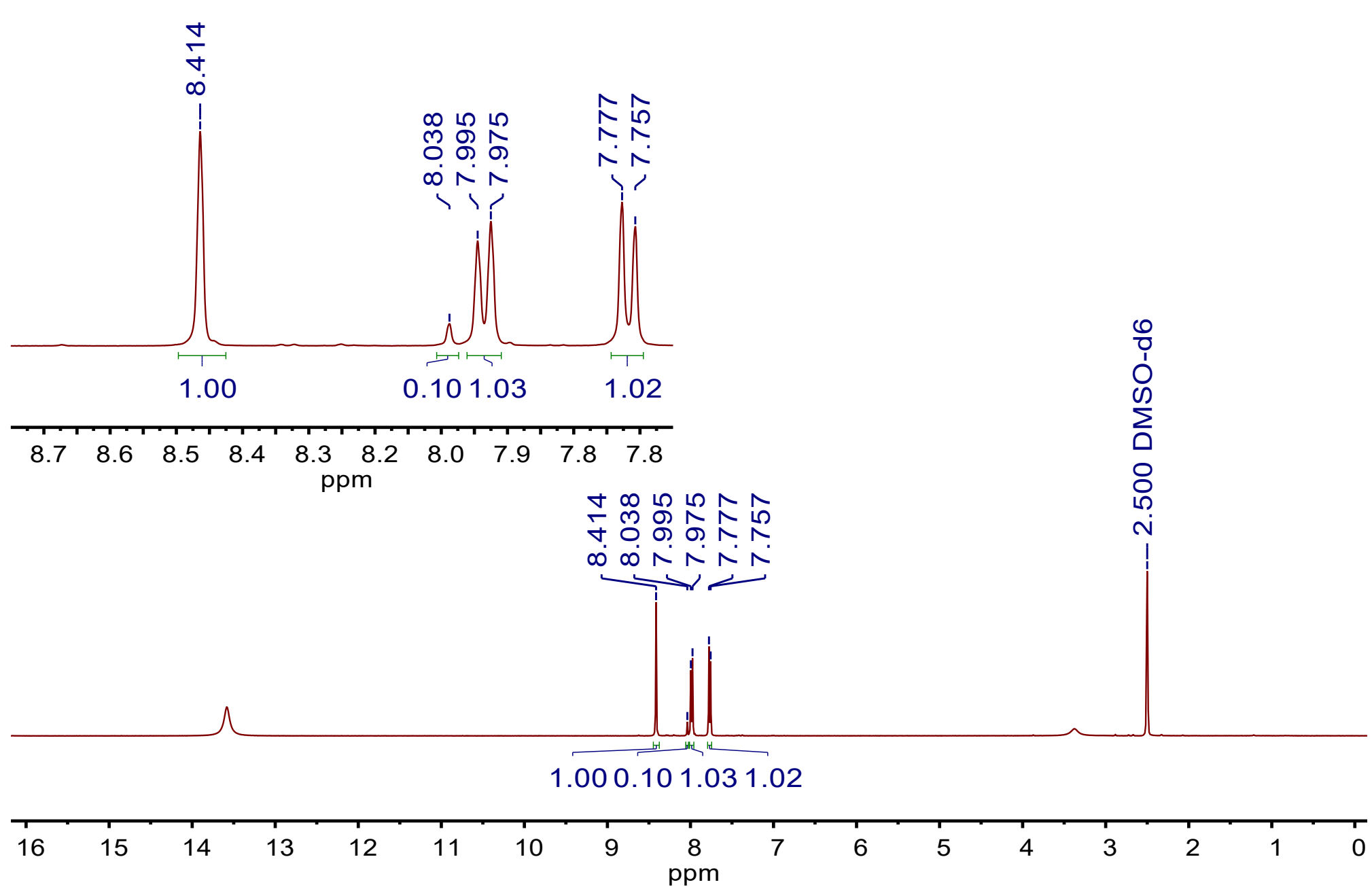

Figure S13 – ^{1}H NMR spectra of commercially available TA-I

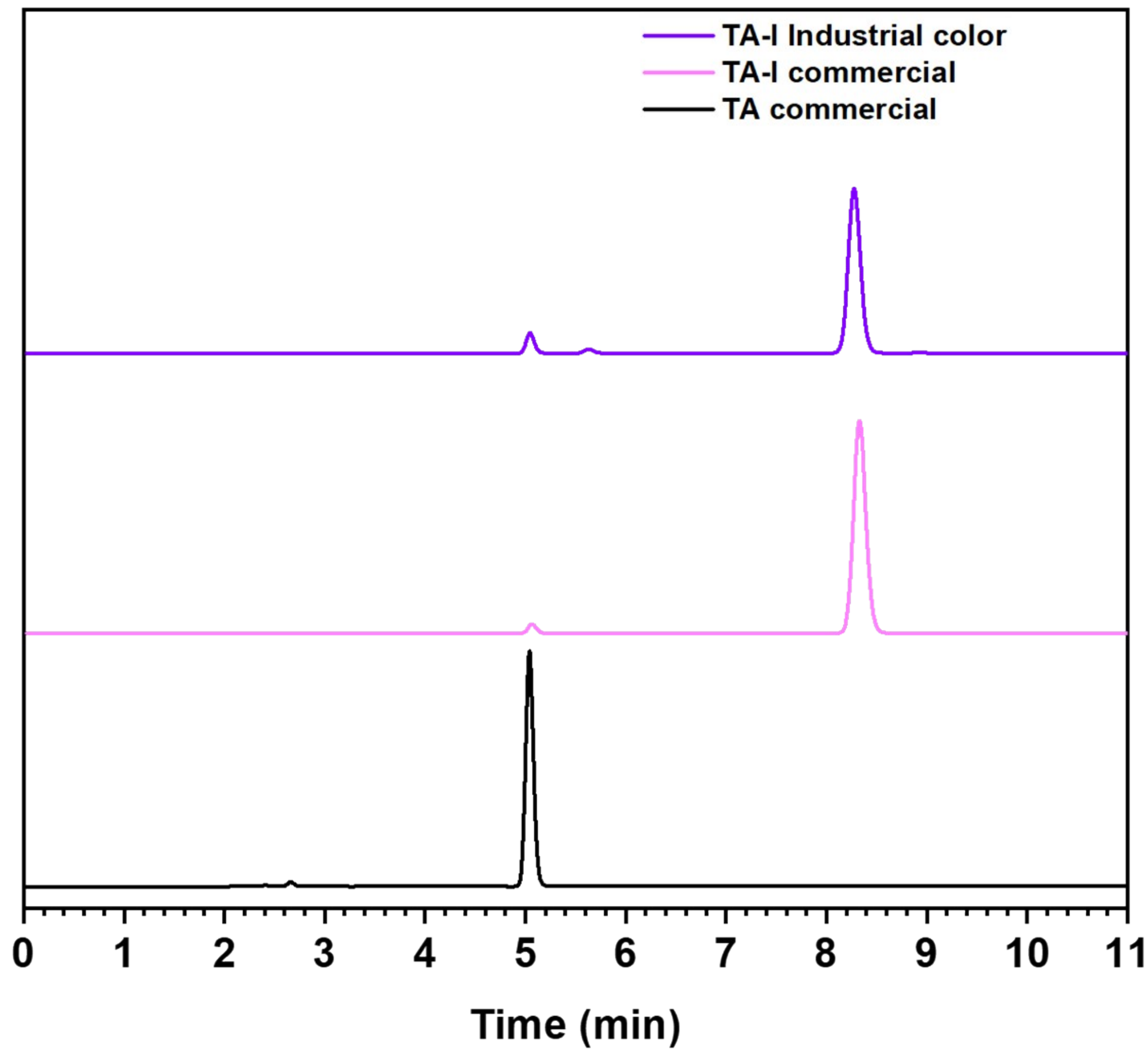

Figure S14 – HPLC chromatograms of isolated TA-I after upcycling of Industrial coloured wPET in comparison with commercial TA and TA-I

Section 4. EEIF Calculation for Conventional Preparation of 2-Iodoterephthalic Acid

For comparison with the direct PET upcycling route, the EEIF was estimated for a conventional three-step synthesis of 2-iodoterephthalic acid starting from TA. Total EEIF for this route and was 357.2 × 10^4 °C·min.

Process 1. Nitration

TA was converted to 2-nitroterephthalic acid using HNO_3/oleum mixture at 90 °C for 4 h.[6]

Table S1 – Calculation of EEIF for the first (Nitration) step

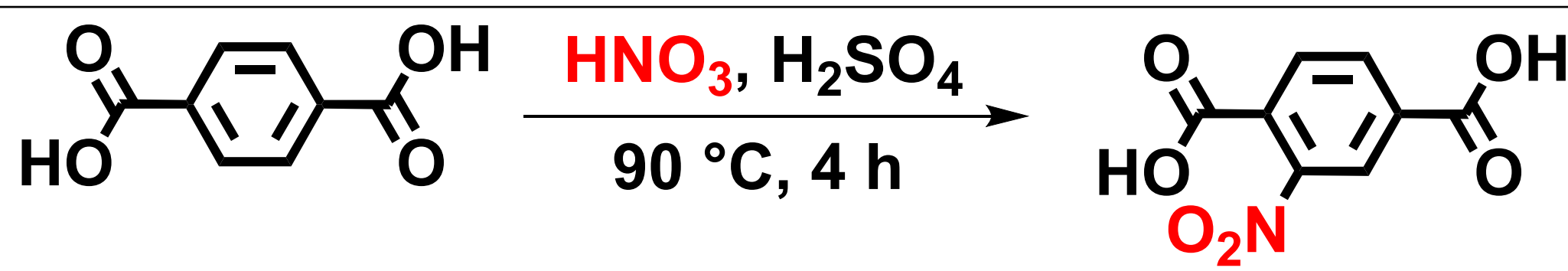

**Reagent/ product**	**Amount, g**	**Temperature, °C**	**Time, min**	**Yield, %**	**EEIF, $\times 10^{-4}$ °C·min**
TA	2				
HNO_3	9.1				
H_2SO_4	15.35	90	240	92	64.6
H_2O (Ice)	39.9				
TA-NO_2	2.34				

Process 2. Reduction

2-Nitroterephthalic acid was reduced to 2-aminoterephthalic acid using H2/Pd-C in alkaline medium.[7]

Table S2 – Calculation of EEIF for the second (Reduction) step

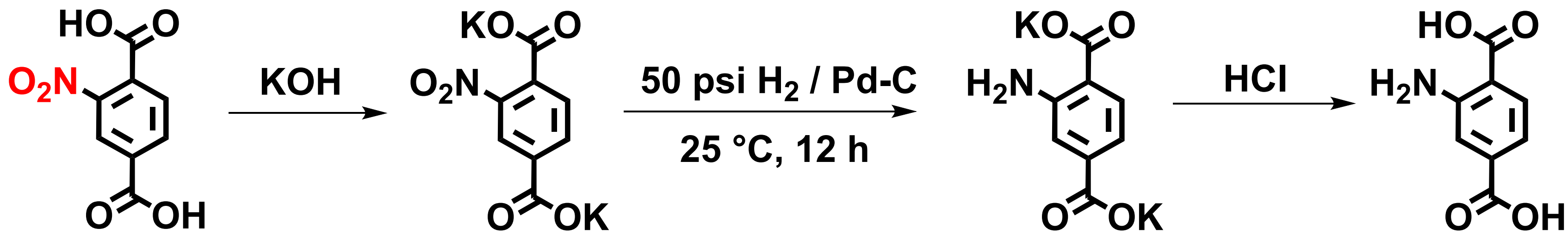

Reagent/ product	Amount, g	Temperature, °C	Time, min	Yield, %	EEIF, × 10^{-4} °C·min
TA-NO_2	2.34				
H_2O	18.71				
KOH	1.21				
Pd-C	0.05	25	720	90	22.5
H_2	0.07				
HCl	0.79				
TA-NH_2	1.81				

Process 3. Diazotization–iodination

2-Aminoterephthalic acid was converted to TA-I through diazotization with $NaNO_2$ followed by iodination using KI.[8]

Table S3 – Calculation of EEIF for the third (Diazotisation[a]/iodination) step

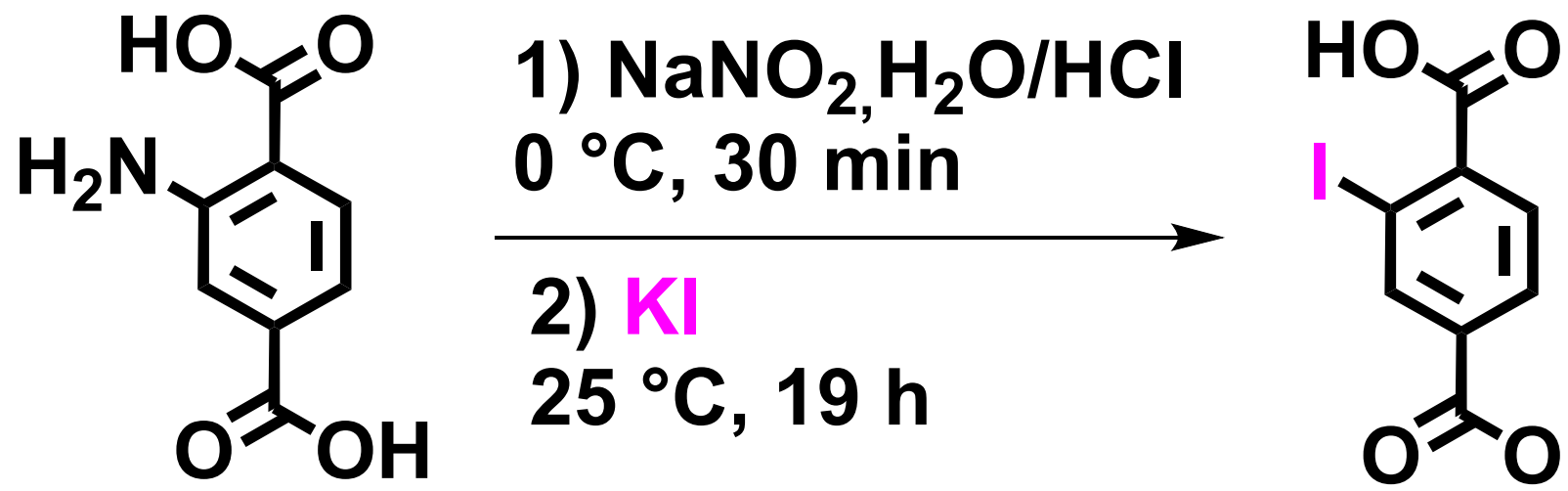

Reagent/ product	Amount, g	Temperature, °C	Time, min	Yield, %	EEIF, × 10^{-4} °C·min
TA-NH_2	1.81	25	1140	90	270.1
H_2O	153.5				
HCl	27.1				
$NaNO_2$	1.7				
KI	9.9				
$Na_2S_2O_3$	9.5				
$CHCl_3$	26.9				
TA-I	2.62				

[a] Due to the low temperature of the diazotisation stage (0 °C), the EEIF for that was not calculated, however we can't ignore the consumption of reagent, so their masses were taken into account when calculating the EEIF for the iodination stage

Section 5. PET nitration mechanism

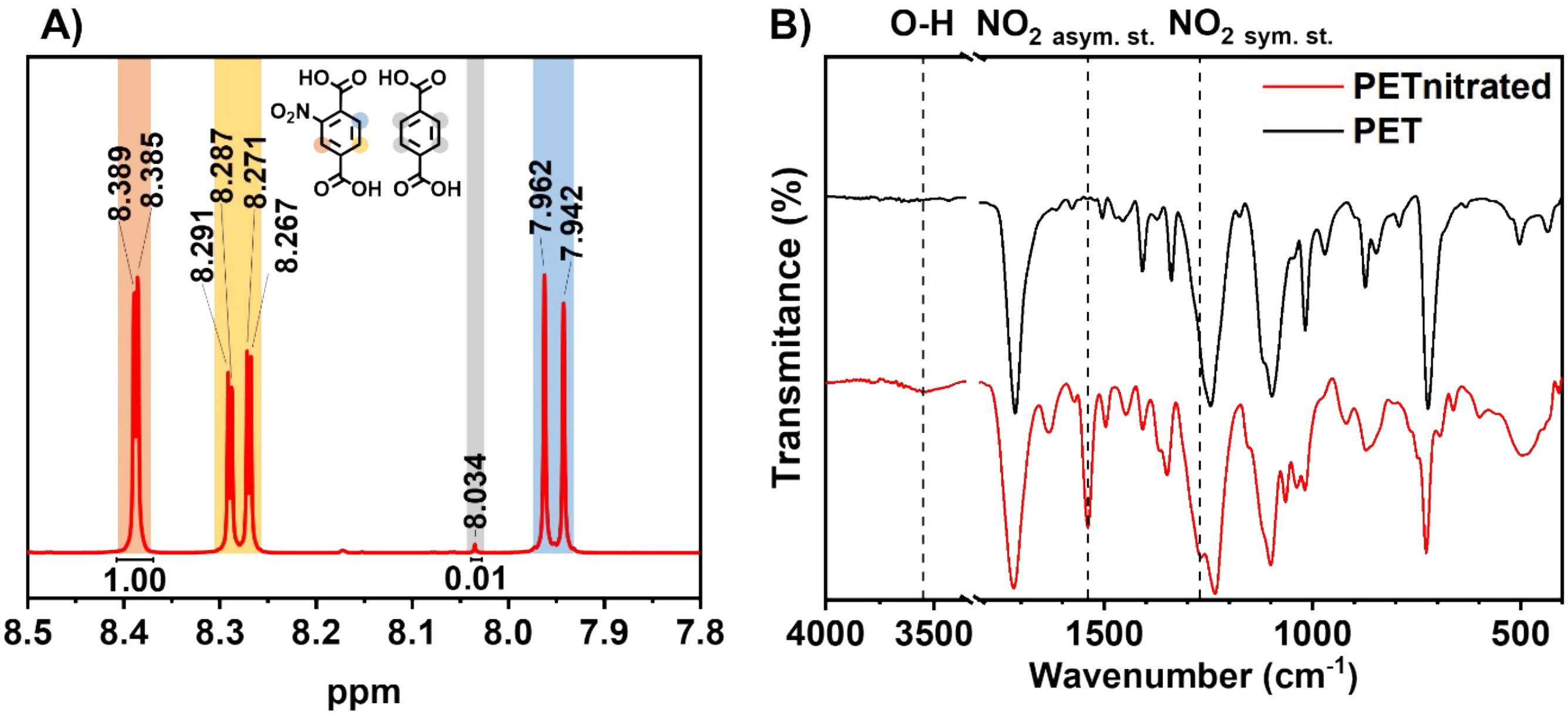

Figure S15 – Dual nitration pathway for PET upcycling: A) ^{1}H NMR spectrum of $TA\text{-}NO_2$ after TA nitration; B) FTIR spectra of wPET before and after partial nitration.

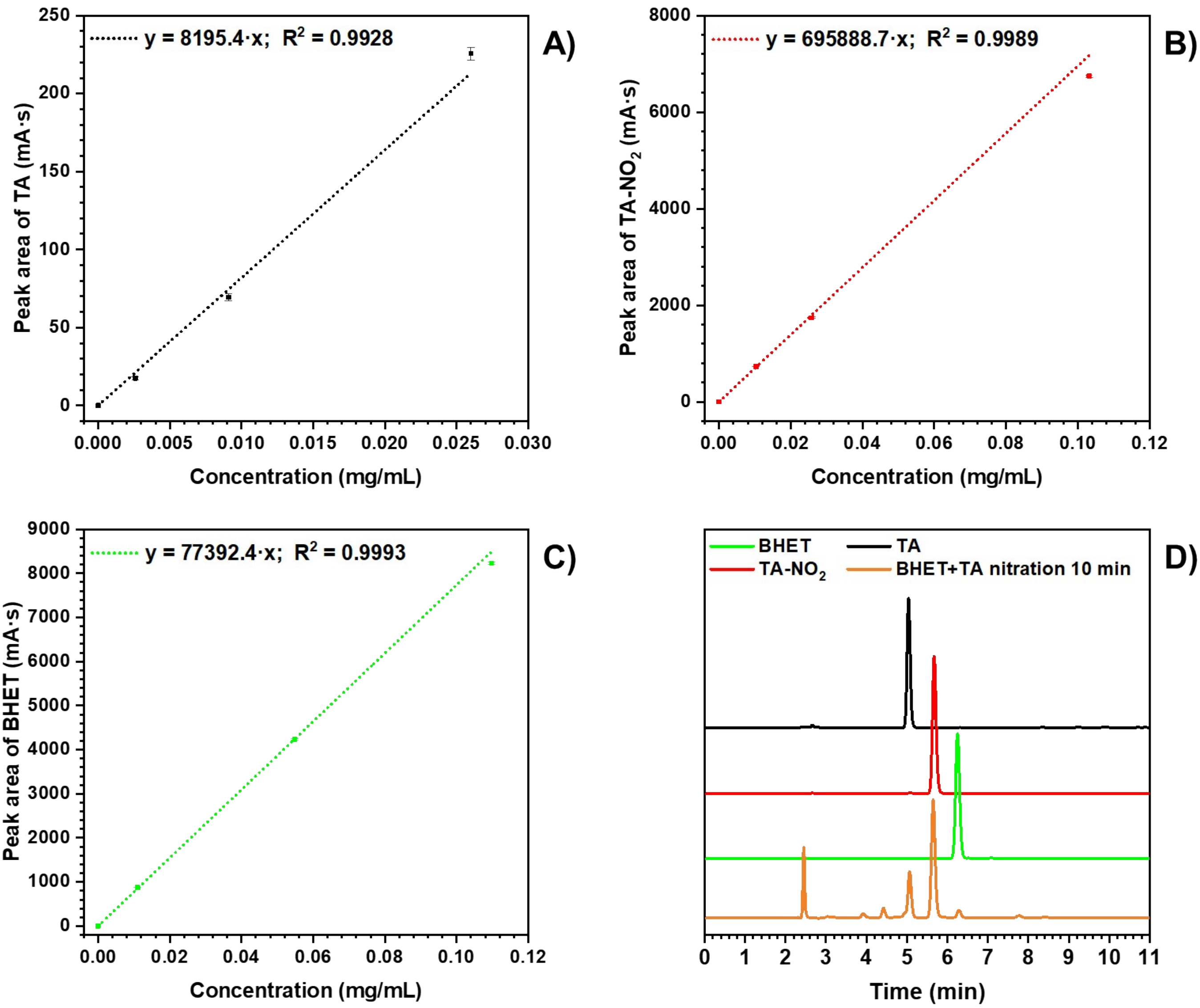

Figure S16 – HPLC calibration curve of TA (A), TA-NO2 (B) and BHET(C) and chromatograms of the same reagents and reaction mixture after 10 minutes of BHET+TA nitration in comparison with commercial reagents. The observed difference in the peak area of TA and TA-NO_2 after 10 min is associated with extinction coefficients

Section 6. Characterization of synthesized UiO-66-X

Table S4 – Analysis of XRD patters on Figure 11 B according to Scherrer (Equation S5).[9]

Sample	Peak position (2θ, °)	FWHM (°)	Crystallite size (nm)
UiO-66-NO_2 (commercial)	7.76	0.59	13.5
UiO-66-NO_2 (Food box)	7.76	0.59	13.5
UiO-66-NO_2 (Industrial Coloured)	7.43	0.42	18.9
UiO-66-NO_2 (Textile Blue)	7.45	0.34	23.8
UiO-66-I (Industrial Coloured)	7.62	0.21	25.3

$$D = \frac{K\lambda}{\beta\cos(\theta)} \times 100 \tag{S5}$$

where, D - crystallite size (nm); K- shape factor (≈ 0.9, approximately spherical, randomly oriented nanocrystals); λ - X-ray wavelength (for Cu Kα, 0.15406 nm); β - FWHM of the diffraction peak in radians; θ - Bragg angle (in radians; half of 2θ)

Table S5 - BET surface area and pore volume for synthesized UiO-66-R and literature data

PET source	TA-R	BET surface area, m^2/g	Pore volume, cm^3/g	Ref.
-	TA-$NO_{2commercial}$	977	0.528	This work
Food box	TA-NO_2	801	0.380	This work
Industrial coloured	TA-NO_2	719	0.362	This work
Textile blue	TA-NO_2	709	0.367	This work
-	TA-$NO_{2commercial}$	706	0.47-	[10]
-	TA-$NO_{2commercial}$	860	-	[1]
Industrial coloured	TA-I	782	0.362	This work
- (from DMIT*)	TA-I	897	-	[11]
- (from DMIT)	TA-I	690	-	[12]

DMIT* – dimethyl 2-iodoterephalate

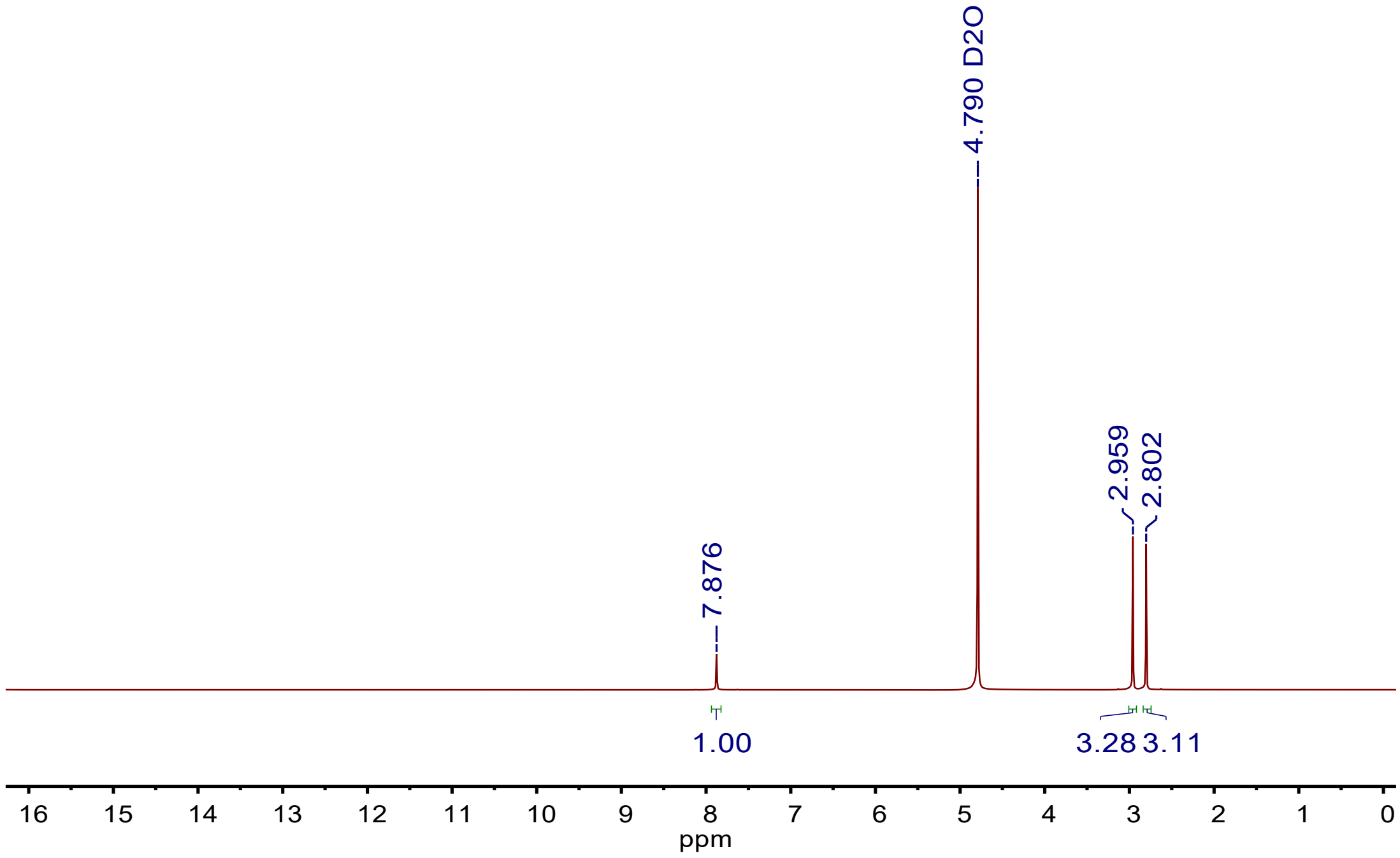

Figure S17 - 1H NMR spectrum of DMF in 1M $NaHCO_3$ D_2O

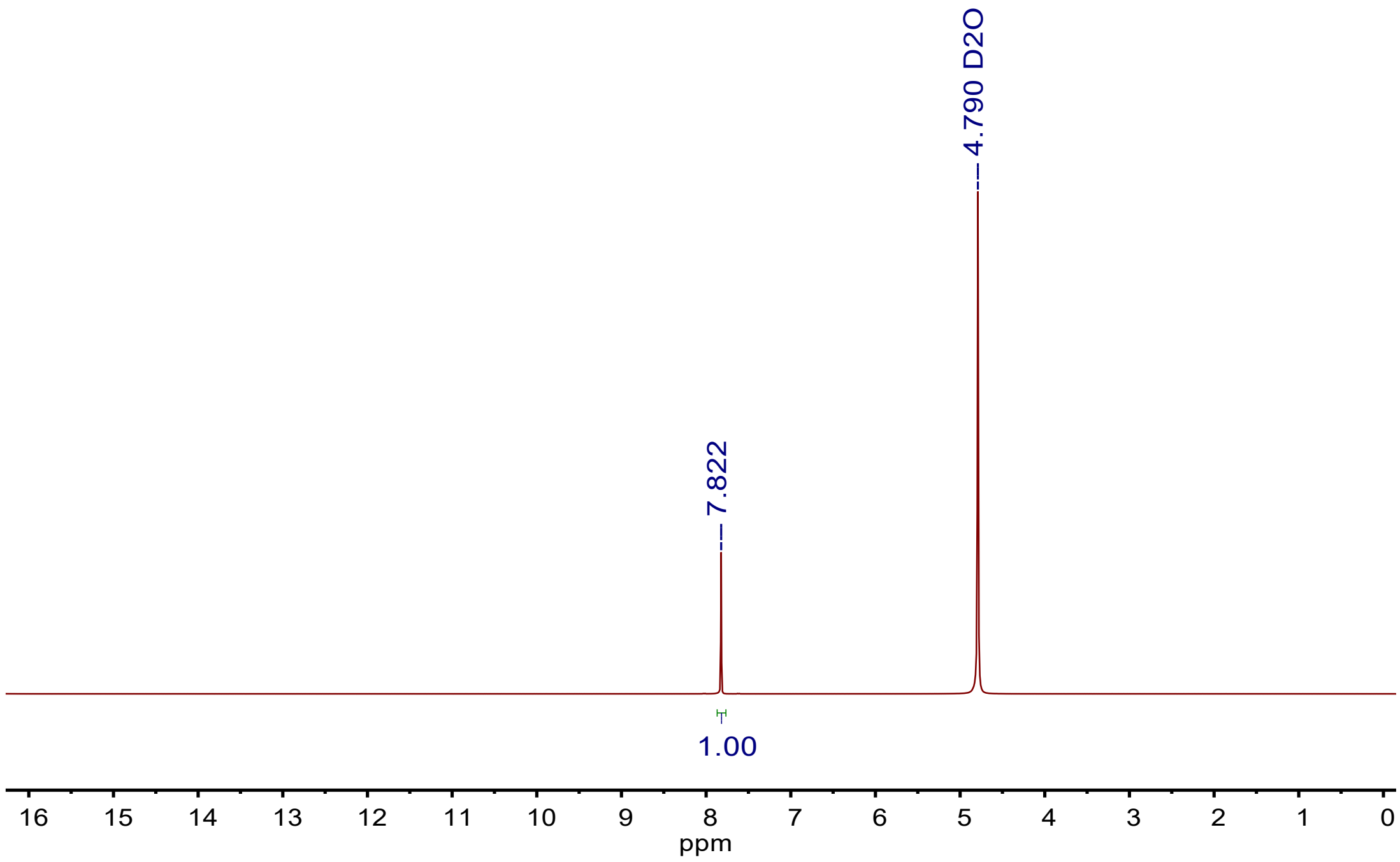

Figure S18 - 1H NMR spectrum of TA commercial in 1M $NaHCO_3$ D_2O

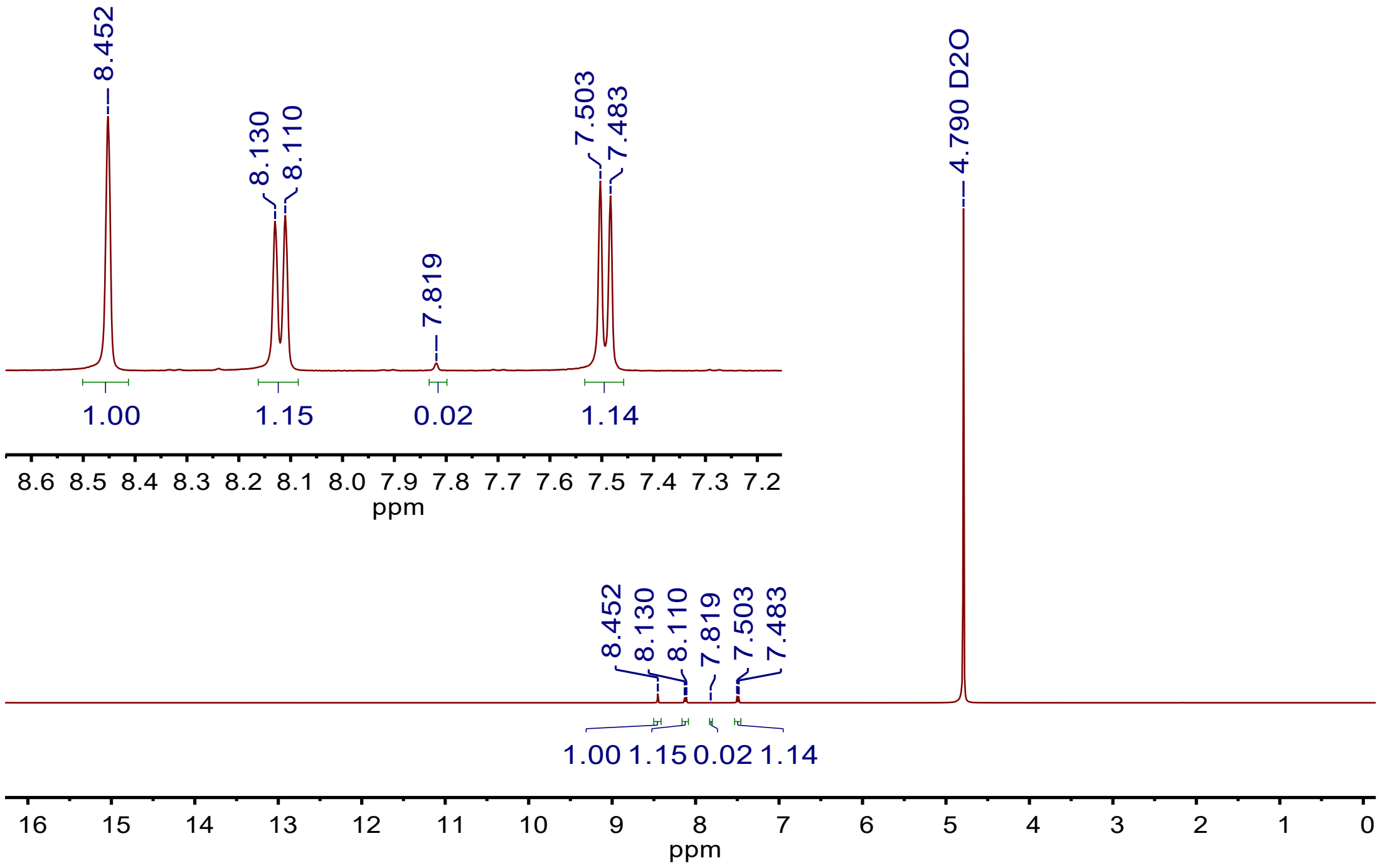

Figure S19 - 1H NMR spectrum of TA-NO_2 commercial in 1M $NaHCO_3$ D_2O

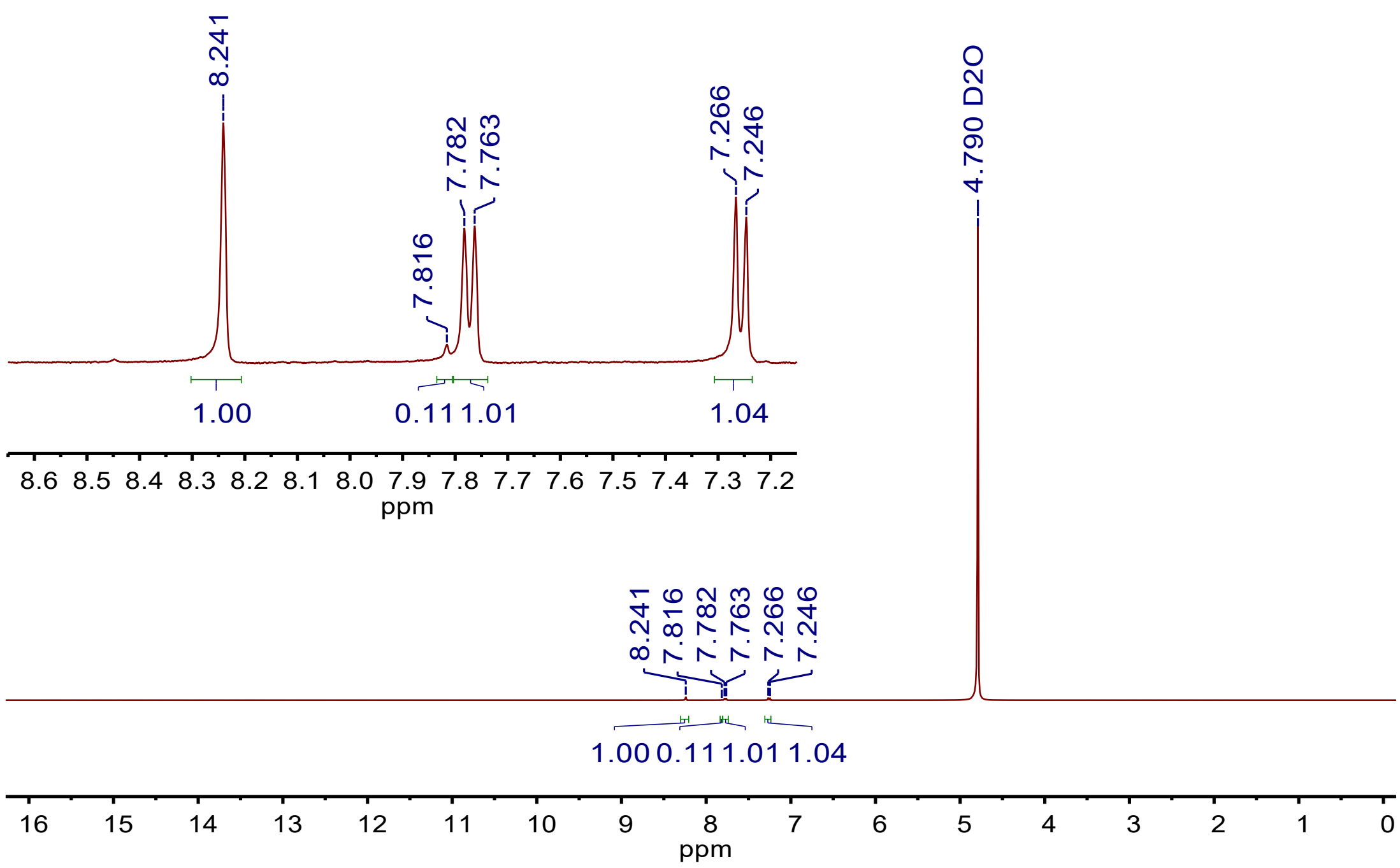

Figure S20 - 1H NMR spectrum of TA-I commercial in 1M $NaHCO_3$ D_2O

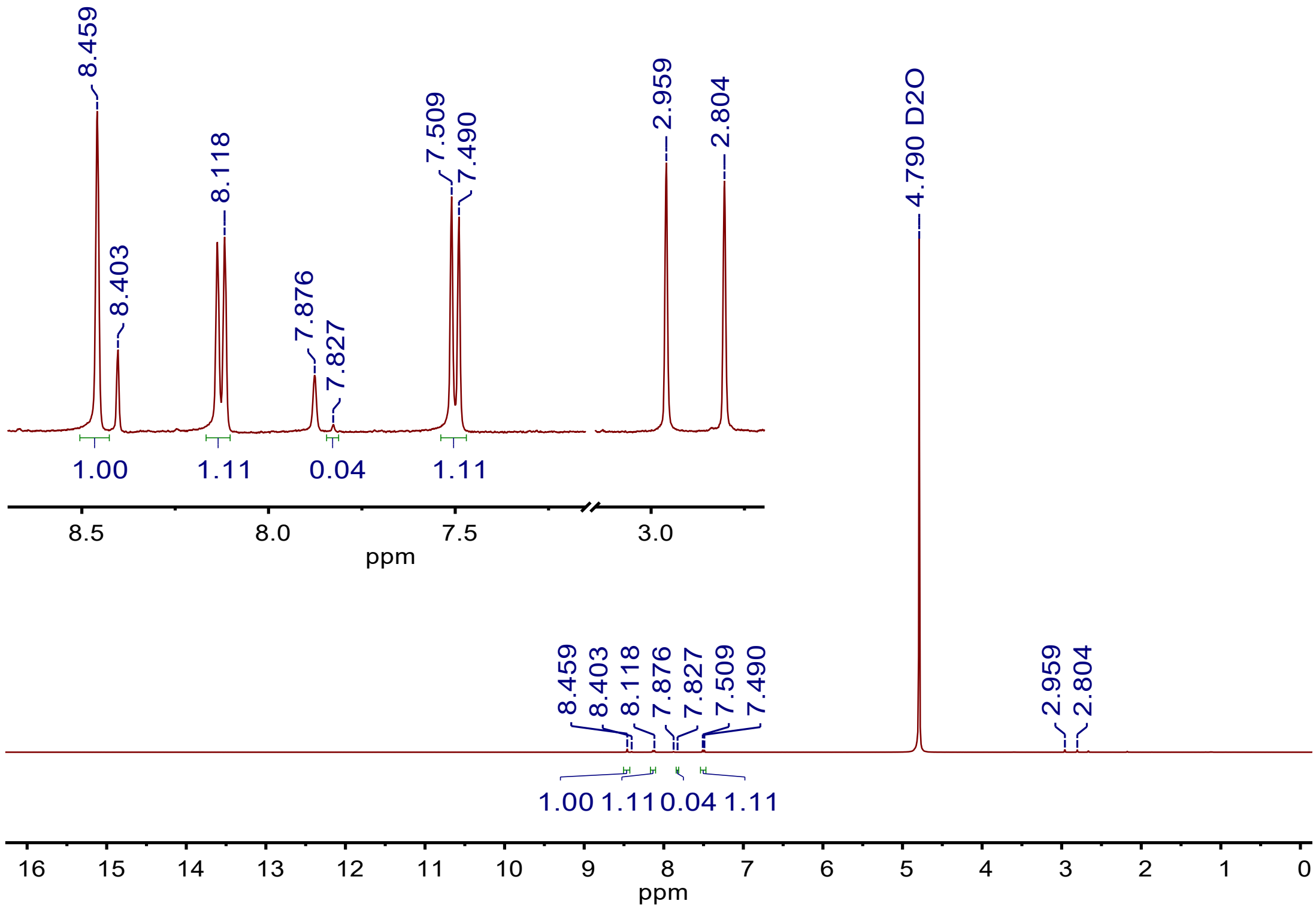

Figure S21 - 1H NMR spectrum of digested UiO-66-NO_2 commercial in 1M $NaHCO_3$ D_2O

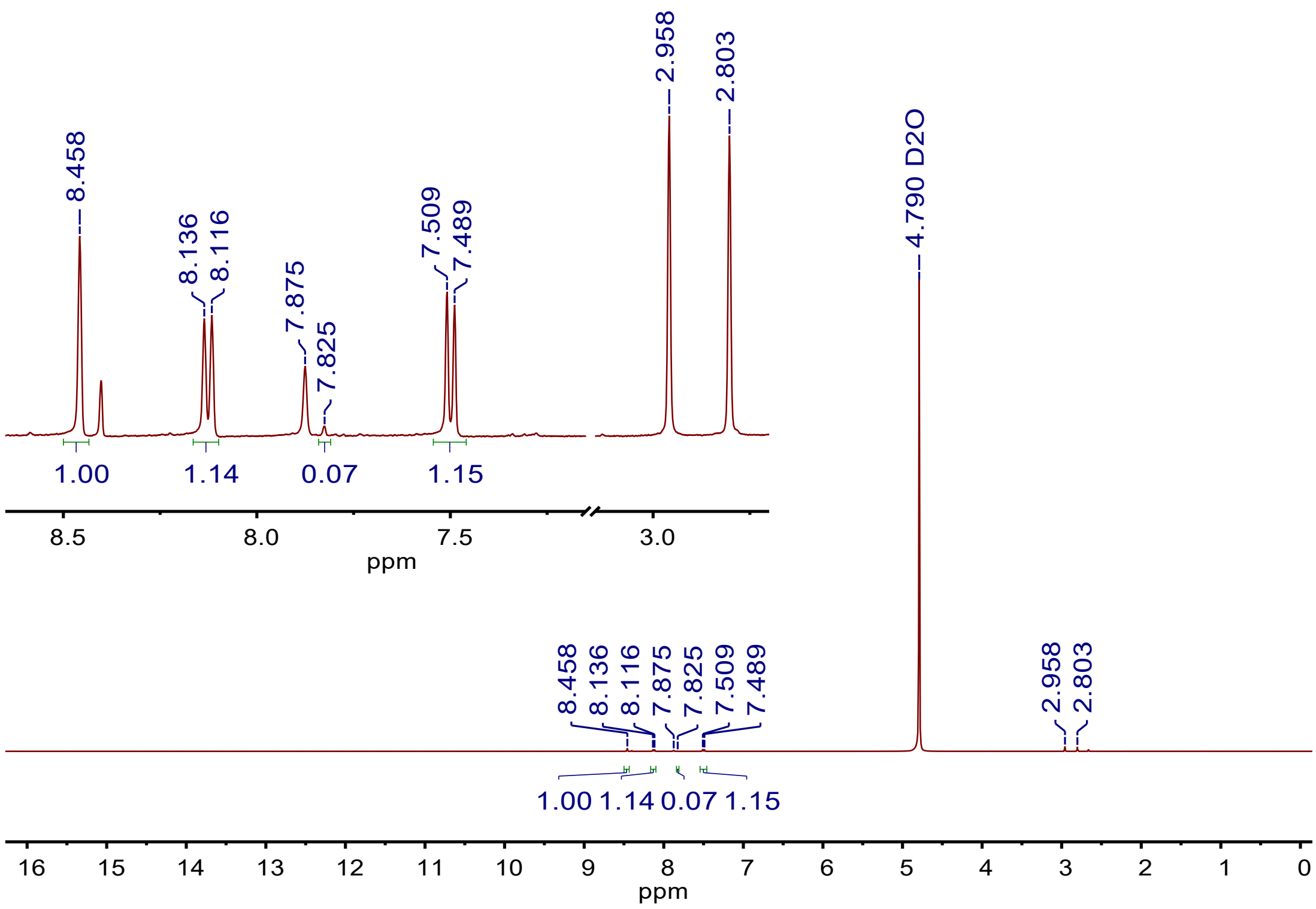

Figure S22 - 1H NMR spectrum of digested UiO-66-NO_2 Food box in 1M $NaHCO_3$ D_2O

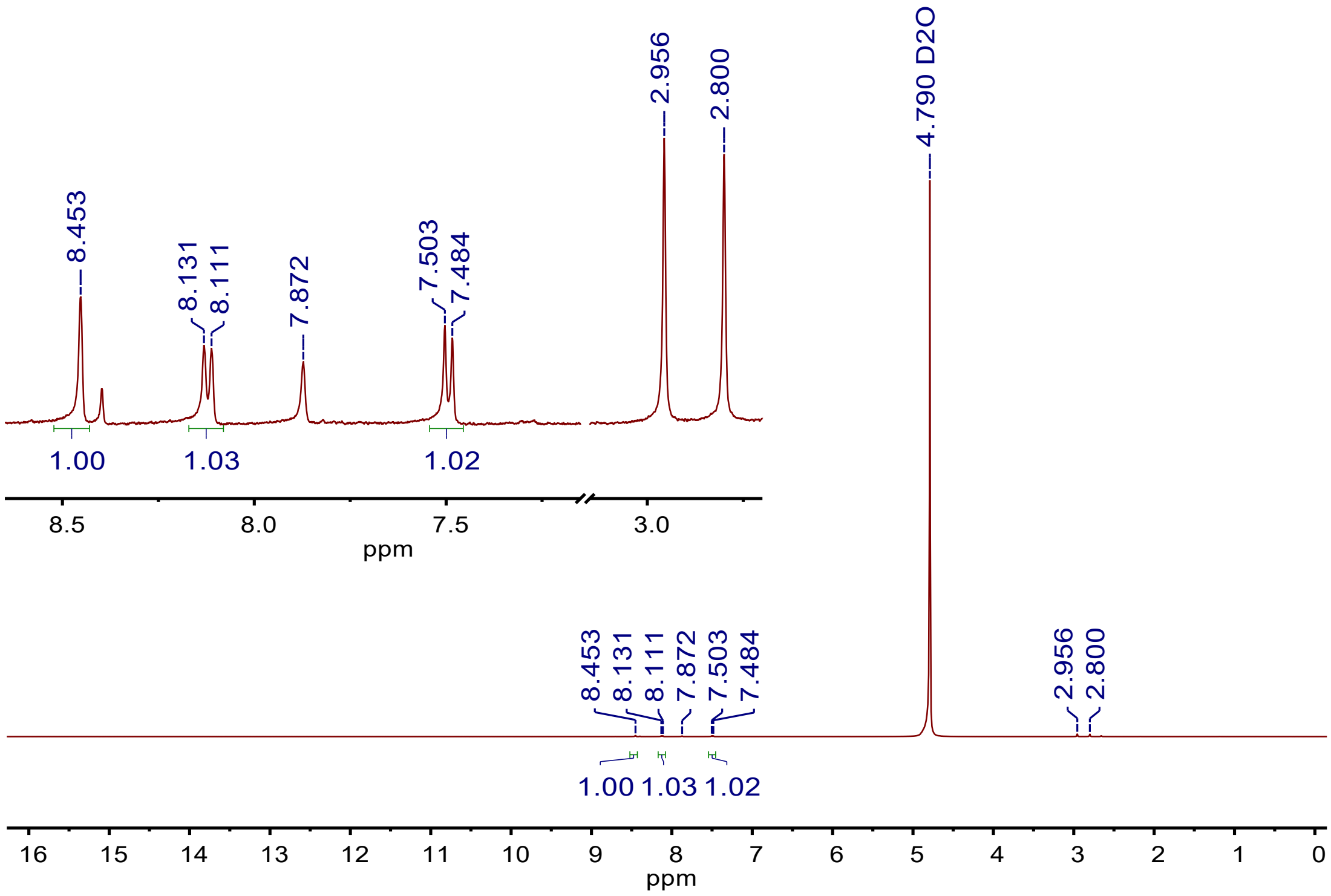

Figure S23 - ^{1}H NMR spectrum of digested UiO-66-NO_2 Industrial colored in 1M $NaHCO_3$ D_2O

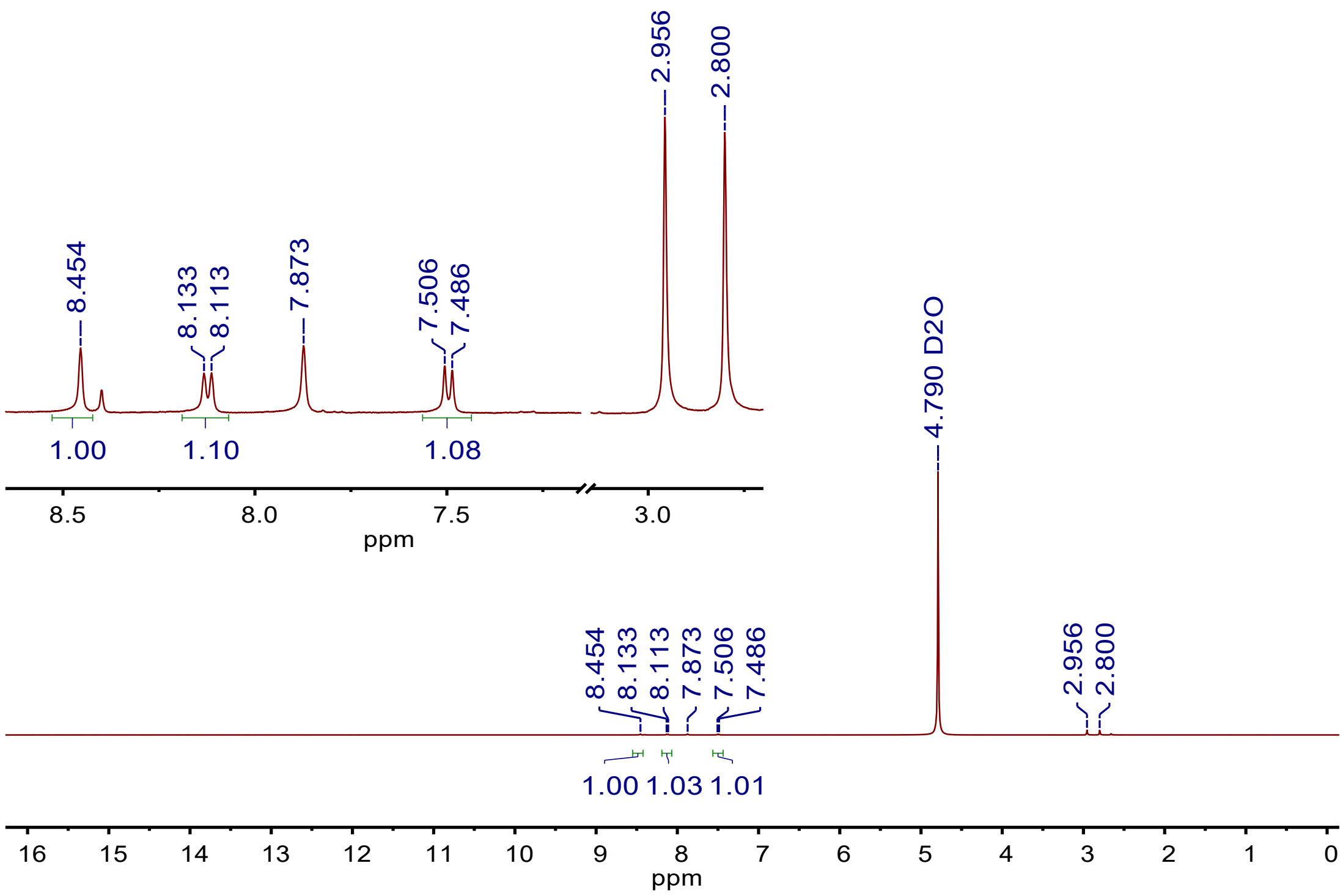

Figure S24 - 1H NMR spectrum of digested UiO-66-NO_2 Textile blue in 1M $NaHCO_3$ D_2O

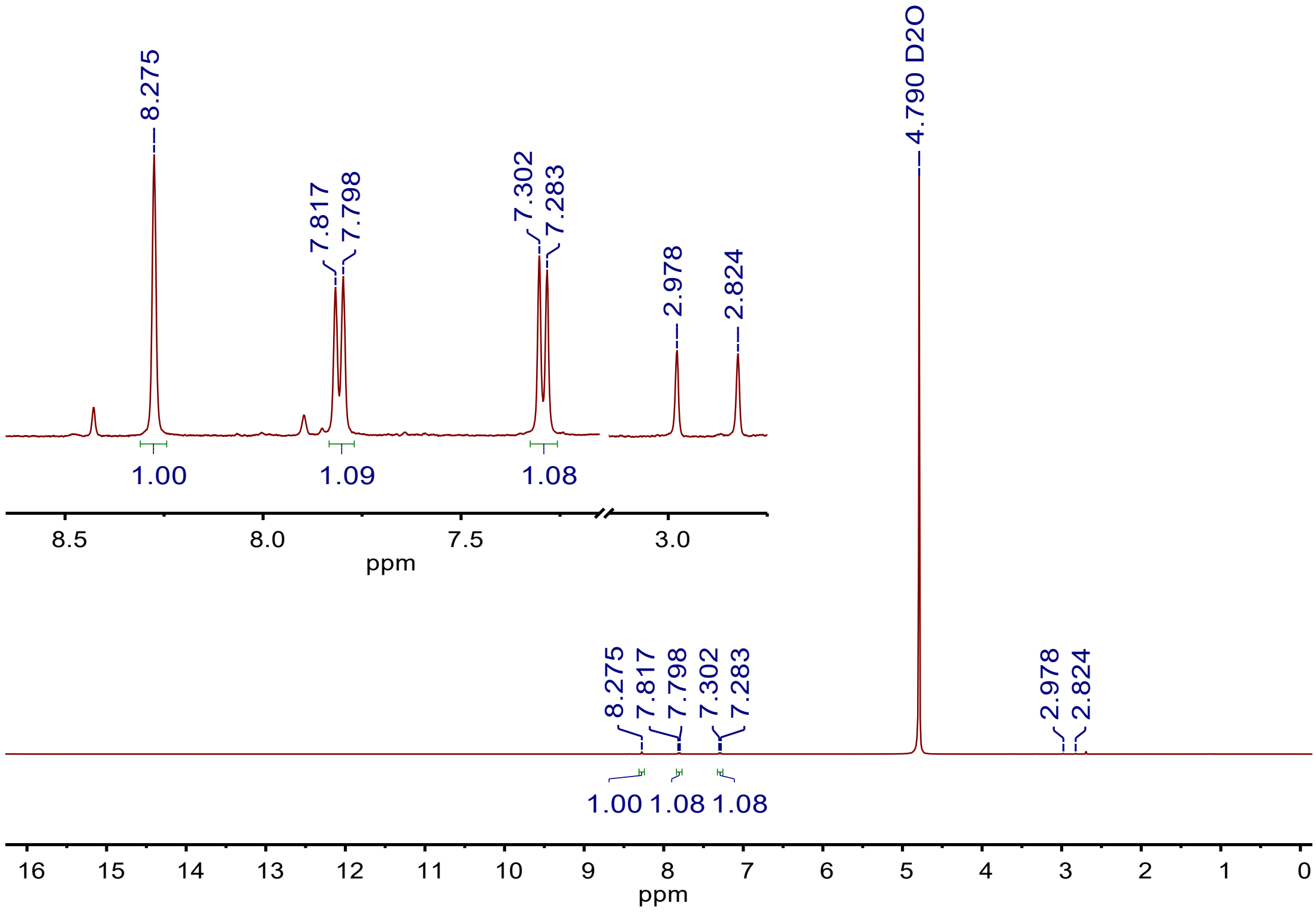

Figure S25 - 1H NMR spectrum of digested UiO-66-I commercial in 1M $NaHCO_3$ D_2O

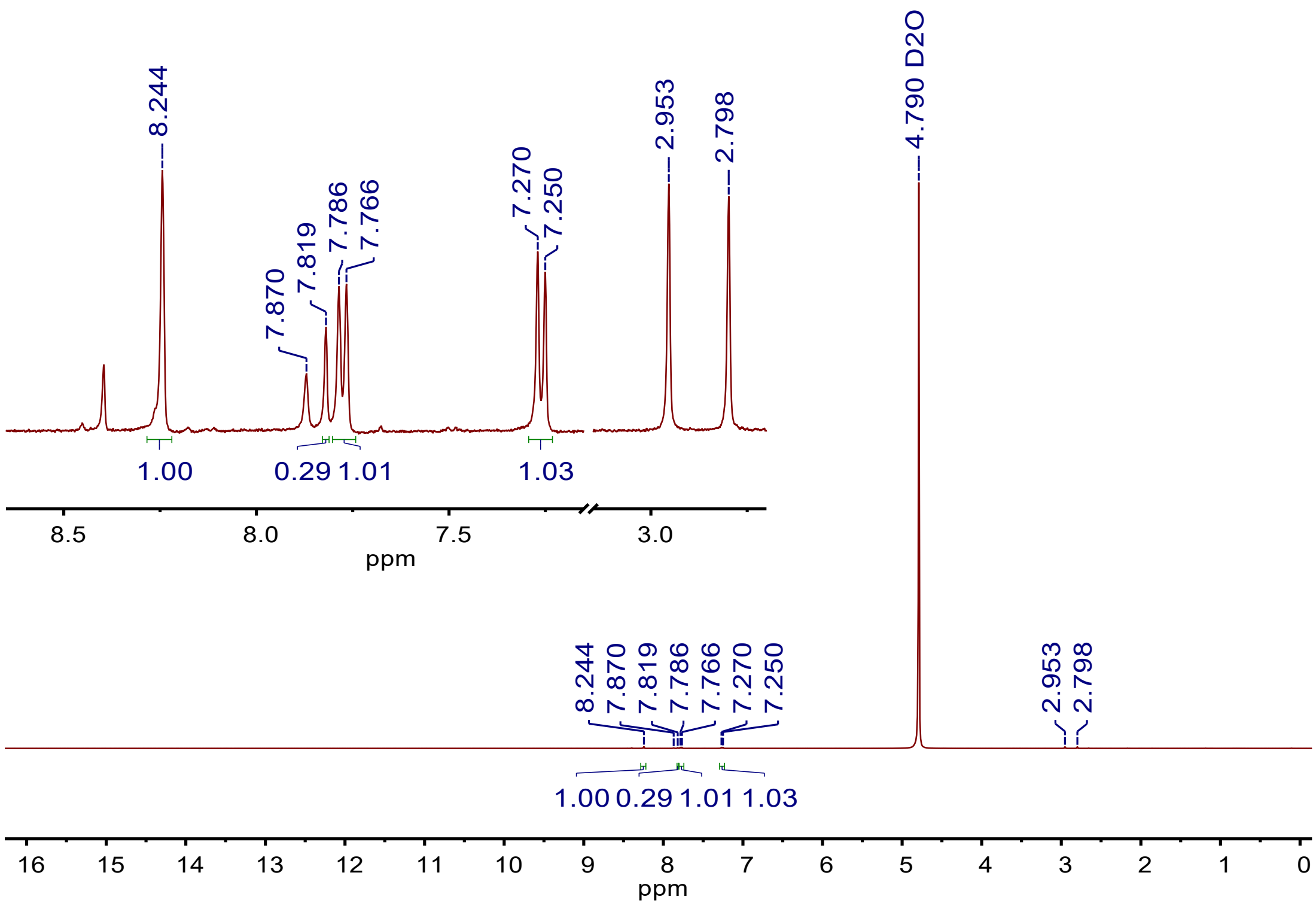

Figure S26 - 1H NMR spectrum of digested UiO-66-I Industrial colored in 1M $NaHCO_3$ D_2O

Section 7. Estimated reagent cost of PET-Derived TA-X linkers and UiO-66-X MOFs

All calculations were scaled to 1 kg of waste PET (wPET) as feedstock.

Table S6. Reagent cost for the synthesis of TA–NO_2 from 1 kg wPET

Reagent	Loading per 2 g PET	Price (€/unit)	Cost per 2 g PET (€)	Cost per 1 kg PET (€)
HNO_3 (65%)	1.8 mL	19.31 €/L	0.035	17.5
H_2SO_4 (98%)	5.4 mL	11.80 €/L	0.064	32.0
NaOH	8 g	9.87 €/kg	0.079	39.5
NaCl	4 g	5.13 €/kg	0.021	10.5
HCl	4mL	10.26 €/L	0.041	20.5
Total reagent cost	–	–	0.24	≈ 120

Yield: 1.91 g TA-NO_2 per 2 g PET → 0.956 kg TA–NO_2 per 1 kg PET Reagent cost per gram: ≈ 0.13 €/g

Table S7. Reagent cost for the synthesis of TA–I from 1 kg wPET

Reagent	Loading per 2 g PET	Price (€/unit)	Cost per 2 g PET (€)	Cost per 1 kg PET (€)
I_2	4.0 g	286 €/kg	1.144	572
$NaNO_3$	3.58 g	15.29 €/kg	0.055	27.5
H_2SO_4 (98%)	15.6 mL	11.80 €/L	0.184	92
NaOH	23 g	9.87 €/kg	0.227	114
Na_2SO_3	2 g	10.41 €/kg	0.021	10.5
HCl	6mL	10.26 €/L	0.062	30.8
Total reagent cost	–	–	1.693	≈ 846

Yield: 2.19 g TA-I per 2 g PET → 1.09 kg TA–I per 1 kg PET Reagent cost per gram: ≈ 0.78 €/g

Table S8. Cost of UiO-66-NO_2 synthesis using commercial vs PET-derived TA–NO_2

Component	Batch loading	Price	Cost per batch (€)
$ZrCl_4$	0.126 g	194 €/kg	0.024
$TA\text{-}NO_2$ (commercial)	0.158 g	0.70 €/g	0.111
$TA\text{-}NO_2$ (from PET)	0.158 g	0.13 €/g	0.016
DMF	10 mL	22.02 €/L	0.220
HCl	1 mL	10.26 €/L	0.010

Table S9. Cost of UiO-66-I synthesis using commercial vs PET-derived TA–I

Component	Batch loading	Price	Cost per batch (€)
$ZrCl_4$	0.126 g	194 €/kg	0.024
TA-I (commercial)	0.219 g	59.2 €/g	12.97
TA-I (from PET)	0.219 g	0.78 €/g	0.162
DMF	10 mL	22.02 €/L	0.220
HCl	1 mL	10.26 €/L	0.010

7.1 Catalytic productivity per unit cost

For citronellal cyclisation, Vermoortele et al.[13] report apparent rate constants k_x at 373 K:

- UiO-66 : $k_H = 0.012\ h^{-1}$
- UiO-66-NO_2: $k_{NO2} = 0.673\ h^{-1}$

Rate enhancement factor:

$$\frac{k_{NO2}}{k_H} = \frac{0.673}{0.012} \approx 56$$

We define a simple metric rate per euro of catalyst (Equation S6):

$$R_{€/g} = \frac{k}{C_{MOF}} [h^{-1} \cdot g \cdot €^{-1}] \qquad (S6)$$

Using the costs from Table S8:

(a) Commercial linker scenario

$$R_H^{comm} = \frac{0.012}{1.59} = 7.55 \times 10^{-3}$$

$$R_{NO2}^{comm} = \frac{0.673}{2.17} = 310 \times 10^{-3}$$

Relative rate per euro:

$$\frac{R_{NO2}^{comm}}{R_{H}^{comm}} = \frac{310 \times 10^{-3}}{7.55 \times 10^{-3}} \approx 41$$

(b) wPET-derived linker scenario

$$R_{H}^{PET} = \frac{0.012}{1.53} = 7.84 \times 10^{-3}$$

$$R_{NO2}^{PET} = \frac{0.673}{1.61} = 418 \times 10^{-3}$$

Relative rate per euro:

$$\frac{R_{NO2}^{PET}}{R_{H}^{PET}} = \frac{418 \times 10^{-3}}{7.84 \times 10^{-3}} \approx 53$$

Thus, linkers sourced from wPET, UiO-66-NO_2 delivers ≈50× higher catalytic activity per euro than UiO-66.

References

1 M. J. Katz, Z. J. Brown, Y. J. Colón, P. W. Siu, K. A. Scheidt, R. Q. Snurr, J. T. Hupp and O. K. Farha, *Chem. Commun.*, 2013, **49**, 9449.

2 A. E. Baumann, D. A. Burns, B. Liu and V. S. Thoi, *Commun. Chem.*, 2019, **2**, 86.

3 T. Aharen, F. Habib, I. Korobkov, T. J. Burchell, R. Guillet-Nicolas, F. Kleiz and M. Murugesu, *Dalt. Trans.*, 2013, **42**, 7795.

4 F. O. Sánchez-Varretti, G. D. García, A. J. Ramirez-Pastor and F. Romá, *J. Chem. Phys.*, DOI:10.1063/1.3139301.

5 2-Nitroterephtalic acid, https://www.carlroth.com/at/en/research-chemicals/2-nitroterephthalic-acid/p/2t24.2, (accessed 1 December 2025).

6 S. H. Sonawane, M. Anniyappan, J. Athar, S. Banerjee and A. K. Sikder, *RSC Adv.*, 2016, **6**, 8495–8502.

7 E. B. Skibo and J. H. Gilchrist, *J. Org. Chem.*, 1988, **53**, 4209–4218.

8 T. Christine, A. Tabey, T. Cornilleau, E. Fouquet and P. Hermange, *Tetrahedron*, 2019, **75**, 130765.

9 P. Scherrer, *Math. Klasse*, 1918, **2**, 98–100.

10 P. Medikonda, *Sep. Sci. Technol.*, 2025, **60**, 605–618.

11 R. T. Jerozal, J. Kim, C. Ma, T. A. Pitt, J.-H. Lee and P. J. Milner, *J. Am. Chem. Soc.*, 2025, **147**, 7127–7136.

12 J. C. Moreton, J. X. Low, K. C. Penticoff, S. M. Cohen and L. Benz, *Langmuir*, 2022, **38**, 1589–1599.

13 F. Vermoortele, M. Vandichel, B. Van de Voorde, R. Ameloot, M. Waroquier, V. Van Speybroeck and D. E. De Vos, *Angew. Chemie Int. Ed.*, 2012, **51**, 4887–4890.
